# The *Verrucomicrobia* LexA-Binding Motif: Insights into the Evolutionary Dynamics of the SOS Response

**DOI:** 10.3389/fmolb.2016.00033

**Published:** 2016-07-20

**Authors:** Ivan Erill, Susana Campoy, Sefa Kılıç, Jordi Barbé

**Affiliations:** ^1^Erill Lab, Department of Biological Sciences, University of Maryland Baltimore CountyBaltimore, MD, USA; ^2^Unitat de Microbiologia, Departament de Genètica i de Microbiologia, Universitat Autònoma de BarcelonaBarcelona, Spain

**Keywords:** SOS response, LexA regulon, DNA repair, comparative genomics, binding motif, translesion synthesis, uracil-DNA glycosylase, regulatory network evolution

## Abstract

The SOS response is the primary bacterial mechanism to address DNA damage, coordinating multiple cellular processes that include DNA repair, cell division, and translesion synthesis. In contrast to other regulatory systems, the composition of the SOS genetic network and the binding motif of its transcriptional repressor, LexA, have been shown to vary greatly across bacterial clades, making it an ideal system to study the co-evolution of transcription factors and their regulons. Leveraging comparative genomics approaches and prior knowledge on the core SOS regulon, here we define the binding motif of the *Verrucomicrobia*, a recently described phylum of emerging interest due to its association with eukaryotic hosts. Site directed mutagenesis of the *Verrucomicrobium spinosum recA* promoter confirms that LexA binds a 14 bp palindromic motif with consensus sequence TGTTC-N4-GAACA. Computational analyses suggest that recognition of this novel motif is determined primarily by changes in base-contacting residues of the third alpha helix of the LexA helix-turn-helix DNA binding motif. In conjunction with comparative genomics analysis of the LexA regulon in the *Verrucomicrobia* phylum, electrophoretic shift assays reveal that LexA binds to operators in the promoter region of DNA repair genes and a mutagenesis cassette in this organism, and identify previously unreported components of the SOS response. The identification of tandem LexA-binding sites generating instances of other LexA-binding motifs in the *lexA* gene promoter of *Verrucomicrobia* species leads us to postulate a novel mechanism for LexA-binding motif evolution. This model, based on gene duplication, successfully addresses outstanding questions in the intricate co-evolution of the LexA protein, its binding motif and the regulatory network it controls.

## Introduction

The SOS response is the primary mechanism for coordinating the response to DNA damage in *Bacteria* (Erill et al., 2007). First reported in *Escherichia coli*, (Little and Mount, 1982), the SOS response has been documented in a broad range of bacterial species (Erill et al., 2007). In *E. coli* and *Bacillus subtilis*, the SOS response has been shown to regulate between 30 and 40 genes involved in DNA repair, translesion synthesis, and cell-division arrest (Fernandez De Henestrosa et al., 2000; Walker et al., 2000; Au et al., 2005). This regulatory network is governed by the transcriptional repressor LexA, which in *E. coli* binds as a homodimer to specific sites upstream of regulated operons and blocks transcription initiation (Thliveris et al., 1991; Walker et al., 2000). Upon DNA damage, the RecA protein binds single-stranded DNA (ssDNA) fragments originating at stalled replication forks, and forms active nucleoprotein filaments capable of promoting self-cleavage of the LexA repressor (Sassanfar and Roberts, 1990). Self-cleavage of the LexA dimer leads to de-repression of target operons, which typically include the *lexA* and *recA* genes (Little, 1991), and full induction of the system (Walker et al., 2000). In recent years, the SOS response has attracted increasing interest due to its active involvement in the regulation of mobile genetic elements, such as integrative and conjugative elements (Beaber et al., 2004), pathogenicity islands (Ubeda et al., 2007), and integron integrases (Guerin et al., 2009), as well as its induction by different types of antibiotics (Beaber et al., 2004; Ubeda et al., 2005; Maiques et al., 2006).

Beyond its clinical interest, the SOS response also constitutes a unique model for the study of the evolution of transcriptional regulatory networks. In contrast with many other transcriptional regulators, the LexA repressor displays remarkably different binding motifs across multiple phyla, changing both the specificity of the dyad region recognized by each LexA monomer as well as the dyad space (Erill et al., 2007). Reported LexA-binding motifs range from short inverted repeats (GAAC-N4-GTTC) in the *Firmicutes* and *Actinobacteria* (Davis et al., 2002; Au et al., 2005), to larger palindromic motifs (CTGT-N8-ACAG) in the *Gammaproteobacteria* (Fernandez De Henestrosa et al., 2000; Erill et al., 2003) and even direct repeat motifs (GTTC-N7-GTTC) in the *Alphaproteobacteria* (Fernandez de Henestrosa et al., 1998; Erill et al., 2004). This variability in LexA-binding motifs is matched by extreme plasticity in the size and composition of the SOS regulatory network, which can regulate from 3 to 40 genes (Fernandez De Henestrosa et al., 2000; Au et al., 2005; Campoy et al., 2005) and has been shown to broadly comprise a minimal shared SOS regulon core consisting of *lexA, recA*, and a mutagenesis gene cassette (*imuA-imuB-dnaE2*; Erill et al., 2006).

Named after *Verrucomicrobium spinosum*, the *Verrucomicrobia* are a recently established bacterial phylum characterized by species with distinct wart-like morphology (Garrity and Holt, 2001) and divided in three main classes (Opitutae, Spartobacteria, and *Verrucomicrobiae*; Bergmann et al., 2011). *Verrucomicrobia* possess several unusual features, like the presence of a eukaryotic-like tubulin (Schlieper et al., 2005), but interest in this phylum has grown in recent years due mainly to metagenomics analyses revealing the association of *Verrucomicrobia* with several eukaryotic hosts (Sait et al., 2011), their prominence in many soil communities (Bergmann et al., 2011) and a significant role in the adaptability of the human gut microbiome (Dubourg et al., 2013; Liou et al., 2013). *Verrucomicrobia* are clustered with the *Planctomycetes* and the *Chlamydiae* in the *Planctomycetes*-*Verrucomicrobia*-*Chlamydiae* (PVC) super-phylum, a large and diverse phylogenetic clade of clinical and biotechnological interest in which the SOS response has not been documented (Gupta et al., 2012). The genome of the representative *Verrucomicrobia* species, *V. spinosum*, reveals the presence of orthologs for the three core constituents of the SOS response (*lexA, recA*, and the *imuA-imuB-dnaE2* operon), suggesting that the *Verrucomicrobia* have a functional SOS response. Here we combine *in silico* and *in vitro* approaches to characterize the LexA-binding motif of *Verrucomicrobia* and analyze the SOS regulatory network of this bacterial phylum. Our results illustrate the extraordinary plasticity of this transcriptional regulatory network and provide novel insights into the molecular mechanisms driving its evolution.

## Materials and methods

### Functional and taxonomical assignments

Orthology assignment of putative function for all reported genes was performed using the HHPRED web service (HHpred, RRID:SCR_010276) with default options and an *e*-value threshold of 10^−30^ on the TIGRFAM (JCVI TIGRFAMS, RRID:SCR_005493), PFAM (Pfam, RRID:SCR_004726), and COG (COG, RRID:SCR_007139) databases (Tatusov et al., 2000; Söding et al., 2005; Haft et al., 2013; Finn et al., 2016). eggNOG identifiers, categories, and descriptions were retrieved from the eggNOG database (eggNOG, RRID:SCR_002456) using HMMER (Hmmer, RRID:SCR_005305; Eddy, 2011; Powell et al., 2014). Taxonomical identification numbers (TaxID) for all species analyzed in the manuscript were retrieved from the NCBI Taxonomy server (NCBI Taxonomy, RRID:SCR_003256; Federhen, 2012).

### Ortholog detection and motif discovery

Protein sequences for *V. spinosum* DSM 4136 [TaxID: 240016] LexA [WP_009959117], RecA [WP_009965965], and ImuA [WP_009959308] were obtained from the NCBI RefSeq database [RefSeq, RRID:SCR_003496; Pruitt et al., 2012]. The promoter regions ([–250, 0] with respect to the translational start site) for orthologs of these sequences in *Verrucomicrobia* genome and metagenome assemblies were downloaded from the Integrated Microbial Genomes (IMG) resource [IMG, RRID:SCR_007733; Markowitz et al., 2014], after identifying orthologs through the IMG BLAST service using a 10^−20^ *e*-value threshold and the *V. spinosum* sequences as queries (Altschul et al., 1997). Downloaded promoter sequences were used as input for the MEME motif discovery tool on the MEME Suite server [MEME Suite—Motif-based sequence analysis tools, RRID:SCR_001783], requesting palindromic motifs between 8 and 20 bp long and otherwise default options (Bailey et al., 2006). Sequence logos were generated using the Weblogo sequence service (WEBLOGO, RRID:SCR_010236; Crooks et al., 2004).

### Analysis of α3 helix motifs

A sequence model for the *Verrucomicrobia* α3 helix of the LexA N-terminal helix-turn-helix motif was obtained through multiple sequence alignment of available LexA protein sequences for this phylum, using the information in P0A7C2 (UniProtKB, RRID:SCR_004426) on the *E. coli* LexA crystal structure (Zhang et al., 2010) to define penalty masks for CLUSTALW profile alignment mode (Thompson et al., 1994). The *Verrucomicrobia* LexA α3 helix motif was compared to previously compiled LexA α3 helix motifs for different phyla (Sanchez-Alberola et al., 2015) using the TomTom service of the MEME suite (Gupta et al., 2007). Amino acid property plots and differential analysis of the *Verrucomicrobia* LexA α3 helix motif with respect to the *Betaproteobacteria, Actinobacteria*, and *Firmicutes* α3 helix motifs were generated with the iceLogo web service (iceLogo, RRID:SCR_012137; Colaert et al., 2009).

### LexA-binding motif search and comparative genomics analysis

Experimentally validated LexA-binding motifs were downloaded from the CollecTF database (CollecTF, RRID:SCR_014405; Kiliç et al., 2014). Whole genome shotgun assemblies for *Verrucomicrobia* species with total sequence lengths larger than or equal to the smallest complete *Verrucomicrobia* genome (*Methylacidiphilum infernorum* V4, TaxID: 481448; 2,287,145 bp) were obtained from the NCBI RefSeq database (RefSeq, RRID:SCR_003496). LexA-binding motif searches on individual genomes were performed using xFITOM (xFITOM, SCR_014445) with the sequence information content (*R*_*i*_) scoring method and default parameters (Schneider, 1997; Bhargava and Erill, 2010). Comparative genomics analyses were performed with CGB, a collection of Python scripts implementing a computational pipeline for comparative genomics of regulatory transcriptional networks in bacterial genomes. The pipeline is based on previous work (Sanchez-Alberola et al., 2015) and is available under a GPL license on GitHub (http://www.github.com/erilllab/cgb). Given a set of genome assemblies, a transcription factor (TF) and its known binding motif, the pipeline first searches for binding motif instances in the promoter region of all genes (−250, +50 of TLS). Genes in directons with intergenic distance below the mean intergenic distance of each genome are considered to form operons and TF-binding sites identified in the lead operon gene are assigned accordingly to all operon members. The presence of genes with high-scoring TF-binding sites within predicted operons is used to revise operon predictions. Orthologs across all analyzed species are detected as best reciprocal BLAST hits using a 10^−20^ *e*-value threshold. The pipeline summarizes analysis results using a heatmap with species clustered using a distance-based TF tree and a color scheme indicating the presence/absence of orthologs and the score of detected TF-binding sites in the corresponding operon.

### Bacterial strains and culture conditions

*E. coli* (DH5α and BL21; Thermo Fisher Scientific, RRID:SCR_013270) and *V. spinosum* DSM 4136 strains were grown at 37°C in LB (Green and Sambrook, 2012) and at 30°C in M13 media [DSMZ 607; German Collection of Microorganisms and Cell Cultures, RRID:SCR_001711], respectively. Antibiotics were added to the cultures at reported concentrations (Green and Sambrook, 2012).

### Oligonucleotides and DNA techniques

Plasmid isolation, restriction digestion, DNA ligation, transformation, DNA extraction, and PCR were carried out using standard protocols, as described elsewhere (Green and Sambrook, 2012). Restriction enzymes, T4 DNA ligase, DNA polymerase, and the DIG-DNA labeling and detection kit were from Roche (Roche NimbleGen, RRID:SCR_008571). The oligonucleotides used for this work are listed in Supplementary Material 1 and were purchased from Invitrogen (Molecular Probes, RRID:SCR_013318). Mutants of the *V. spinosum recA* promoter (VSP_RS32310) were obtained using oligonucleotides carrying designed substitutions (Supplementary Material 1). The DNA sequence of generated fragments was verified by sequencing (Macrogen, RRID:SCR_014454).

### Protein purification and electrophoresis mobility shift assays

*V. spinosum* DSM 4136 DNA was extracted from phosphate buffered (50 mM) saline (pH 8.0)-washed pellets containing cells using the easy-DNA^TM^ DNA isolation kit (Molecular Probes, RRID:SCR_013318). The *V. spinosum* DSM 4136 was amplified using suitable primers (Supplementary Material 1) and cloned into a pET15b vector (Millipore, RRID:SCR_008983). The *O. terrae* PB90-1 lexA was obtained by chemical synthesis (GeneArt; Thermo Fisher Scientific, RRID:SCR_013270) and cloned into a pET15b vector. Overexpression and purification of the *V. spinosum* DSM 4136 and *O. terrae* PB90-1 and *B. subtilis* LexA proteins was performed as described previously for other LexA proteins (Cambray et al., 2011; Cornish et al., 2014). DNA probes for electro-mobility shift assays (EMSA) were generated using two complementary synthetic oligonucleotides centered on the target LexA-binding sites (Supplementary Material 1). The dsDNA synthetic fragments were ligated into pGEMT vector (Roche NimbleGen, RRID:SCR_008571) and transformed into *E. coli* DH5α (Thermo Fisher Scientific, RRID:SCR_013270). In all cases the plasmids were confirmed by sequencing and DNA probes were obtained by PCR using M13 forward and reverse digoxigenin-labeled oligos (Supplementary Material 1). EMSAs were performed as described previously (Sanchez-Alberola et al., 2012), using 20 ng of each digoxigenin-marked DNA probe in the binding mixture and adding the corresponding LexA protein (from 80 to 400 nM). Samples were loaded onto 6% non-denaturing Tris-glycine polyacrylamide gels and digoxigenin-labeled DNA-protein complexes were detected using the manufacturer's protocol (Roche NimbleGen, RRID:SCR_008571).

## Results

### LexA targets a novel LexA-binding motif in the *Verrucomicrobia*

**Figure 1:**
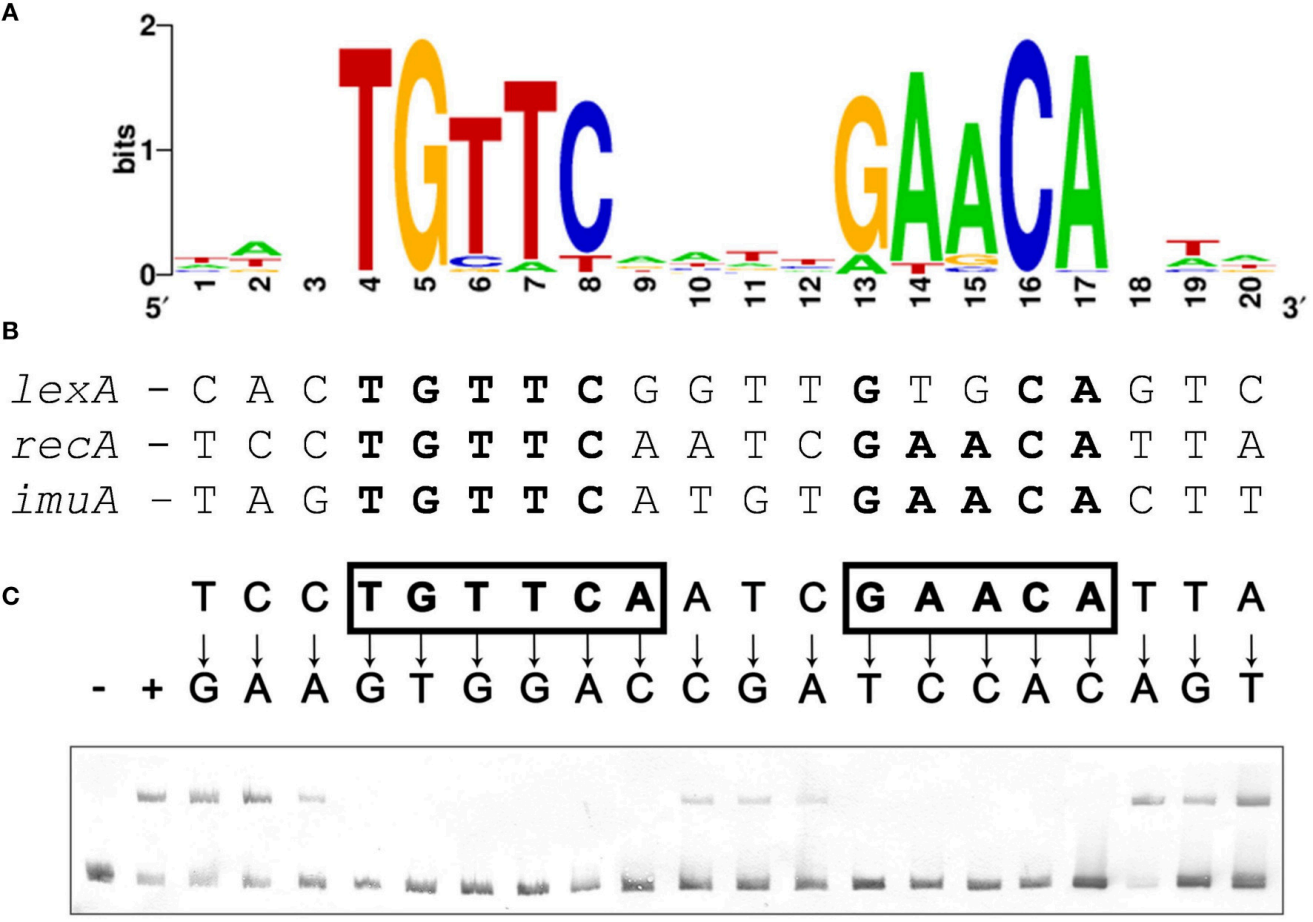
**(A)** Sequence logo of the palindromic motif identified by MEME in the promoter region of *Verrucomicrobia lexA, recA*, and *imuA* genes. **(B)** Computationally identified LexA-binding sites matching the *Verrucomicrobia* LexA-binding motif in the promoter region of *V. spinosum lexA, recA*, and *imuA-imuB-dnaE2* operons. Bases matching the motif consensus are highlighted in bold typeface. **(C)** Electro-mobility shift assays on wild-type and single-nucleotide mutation-containing fragments of the *V. spinosum recA* promoter using *V. spinosum* LexA (80 nM). The “+” and “−” symbols denote, respectively, lanes for the negative control (no LexA protein) and the wild-type *recA* promoter fragment. For all other lanes, arrows designate the introduced single-nucleotide mutations. Positions on which single-nucleotide mutations abolish binding are shown in bold typeface and boxed.

The presence of core SOS response operons (*lexA* [VSP_RS04780], *recA* [VSP_RS32310], and *imuA-imuB-dnaE2* [VSP_RS05590-VSP_RS05595-VSP_RS05600]) in the genome of the representative *Verrucomicrobia* species *V. spinosum* DSM 4136 indicates that this phylum might possess a functional LexA regulatory network. However, computational searches using known LexA-binding motifs did not yield putative LexA-binding sites upstream of any SOS related genes in *V. spinosum*. Taking advantage of the availability of multiple genome and metagenome assemblies for the *Verrucomicrobia* phylum, we compiled 116 promoter sequences from 59 different assemblies corresponding to orthologs of the *V. spinosum* DSM 4136 *lexA, recA*, and *imuA* genes through the JGI-IMG service (Supplementary Material 2). We then used MEME to identify overrepresented motifs in these sequences. The most significant motif identified by MEME (Figure 1A) is a 14 bp palindromic motif with consensus sequence (TGTTC-N4-GAACA). This motif was identified in the promoter region of 27 *lexA* genes, 25 *recA* genes, and 3 *imuA* genes, corresponding to 36 different genome and metagenome assemblies and spanning all three major groups of *Verrucomicrobia* (Supplementary Material 3). A computational search also identified instances of this motif in the promoter sequences of the *V. spinosum* DSM 4136 *lexA, recA*, and *imuA-imuB-dnaE2* operons (Figure 1B). The TGTTC-N4-GAACA motif is reminiscent of the LexA-binding motif (GAAC-N4-GTTC) previously reported in the *Firmicutes, Actinobacteria*, and Gallionellales (Davis et al., 2002; Au et al., 2005; Sanchez-Alberola et al., 2015). Together with its structural similarity to previously reported LexA-binding motifs, the presence of this motif in the promoter region of multiple orthologs for three core components of the SOS response strongly suggested that the identified TGTTC-N4-GAACA motif is the LexA-binding motif of the *Verrucomicrobia*.

To validate that the palindromic motif identified *in silico* was the LexA-binding motif of *V. spinosum*, we purified the *V. spinosum* DSM 4136 LexA protein [WP_009959117] and performed electro-mobility shift assays (EMSA) with wild-type and mutant versions of the *V. spinosum* DSM 4136 *recA* promoter containing single-nucleotide substitutions at each position of the predicted LexA-binding motif. The results of this site-directed mutagenesis analysis (Figure 1C) are in broad agreement with the motif predicted *in silico*, confirming that *V. spinosum* LexA targets a spaced dyad motif with consensus sequence TGTTC-N4-GAACA. Single-nucleotide mutations to the bases of the inverted repeat regions (TGTTC and GAACA) of the *V. spinosum* LexA-binding motif systematically abolish LexA binding in the *recA* promoter context, indicating that these conserved elements likely correspond to the monomer binding site and are therefore essential for LexA binding activity (Groban et al., 2005). In contrast, the 4 bp spacer region and 3 bp flanking regions tolerate single-nucleotide mutations, suggesting that they are predominantly involved in indirect readout and DNA bending (Zhang et al., 2010).

**Figure 2:**
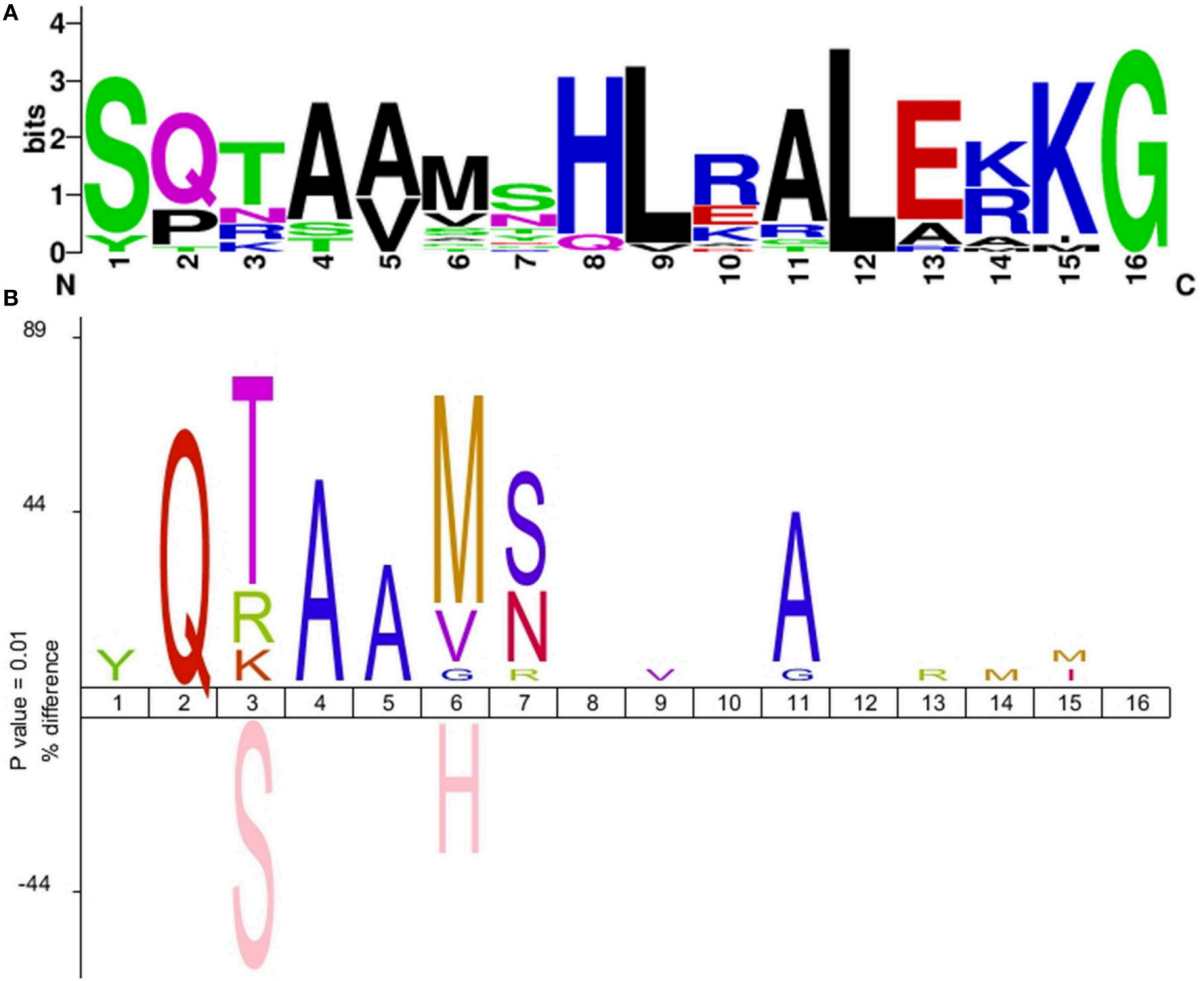
**(A)** Sequence logo of the *Verrucomicrobia* LexA α3 helix motif. **(B)** IceLogo depicting the difference in amino acid frequency at each position of the LexA α3 helix between the multiple sequence alignment of *Verrucomicrobia* LexA protein sequences and a reference set of *Betaproteobacteria, Actinobacteria*, and *Firmicutes* LexA α3 helix sequences. The upper part of the plot shows residues overrepresented in the *Verrucomicrobia* LexA α3 helix; the bottom part shows residues overrepresented in the reference set. Only differences with significant *z*-score under a confidence interval of 0.01 are shown.

Previous work has established that the α3 helix of the N-terminal helix-turn-helix motif is responsible for the majority of the specific contacts with monomer binding sites of LexA-binding motifs (Oertel-Buchheit et al., 1990; Ottleben et al., 1991; Thliveris and Mount, 1992; Groban et al., 2005; Zhang et al., 2010). Comparison of the α3 helix sequence in *Verrucomicrobia* LexA proteins with previously reported LexA α3 helix motifs (Sanchez-Alberola et al., 2015) shows that the *Verrucomicrobia* LexA α3 helix is most closely related to those of the *Betaproteobacteria, Firmicutes*, and *Actinobacteria*. As shown in Figure 2, the majority of the changes observed in the α3 helix of *Verrucomicrobia* localize to the N-terminal part of the helix, affecting residues that change sequence specificity through direct readout, but that are not essential for DNA bending and structural motif recognition (Oertel-Buchheit et al., 1990; Thliveris et al., 1991; Thliveris and Mount, 1992; Groban et al., 2005; Zhang et al., 2010). Furthermore, the overall distribution of hydrogen donors and hydrophobic residues is preserved across the entire α3 helix (Supplementary Material 4). These observations suggest that the structural similarities between *Firmicutes, Actinobacteria, Betaproteobacteria*, and *Verrucomicrobia* LexA-binding motifs are the result of an evolutionary process in the LexA DNA-binding motif that has modified the specific readout of monomer sites without altering the recognition of the overall motif structure.

### The *Verrucomicrobia* LexA protein targets tandem binding sites in *LexA* promoters

**Figure 3:**
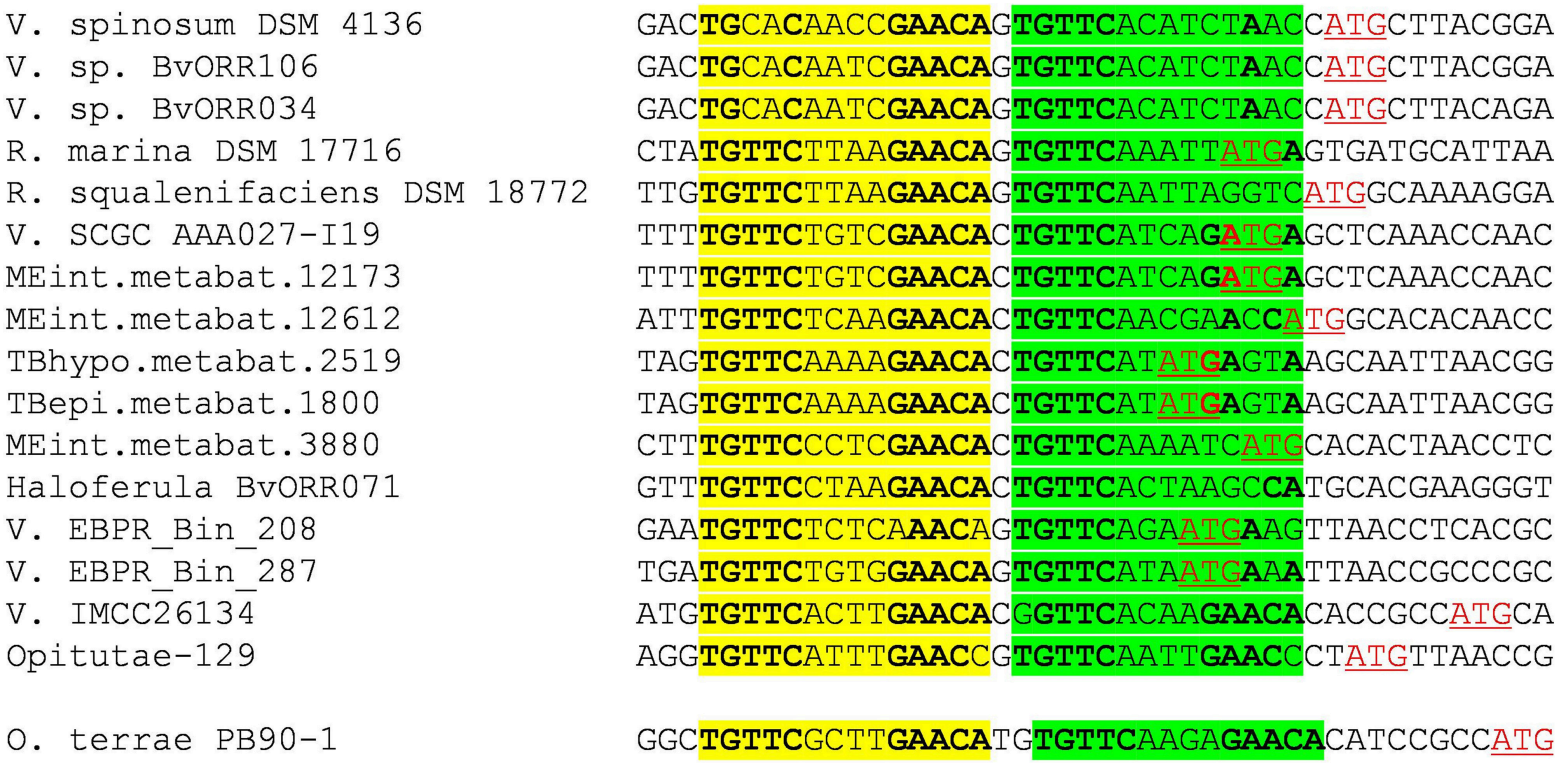
**Multiple sequence alignment of *Verrucomicrobia lexA* promoter sequences displaying the tandem LexA-binding site arrangement observed in *V. spinosum***. The primary LexA-binding site is highlighted in yellow, and the secondary one in green. Bases matching the LexA-binding motif consensus are in bold typeface. Predicted translation start sites are shown in red and underlined.

**Figure 4:**
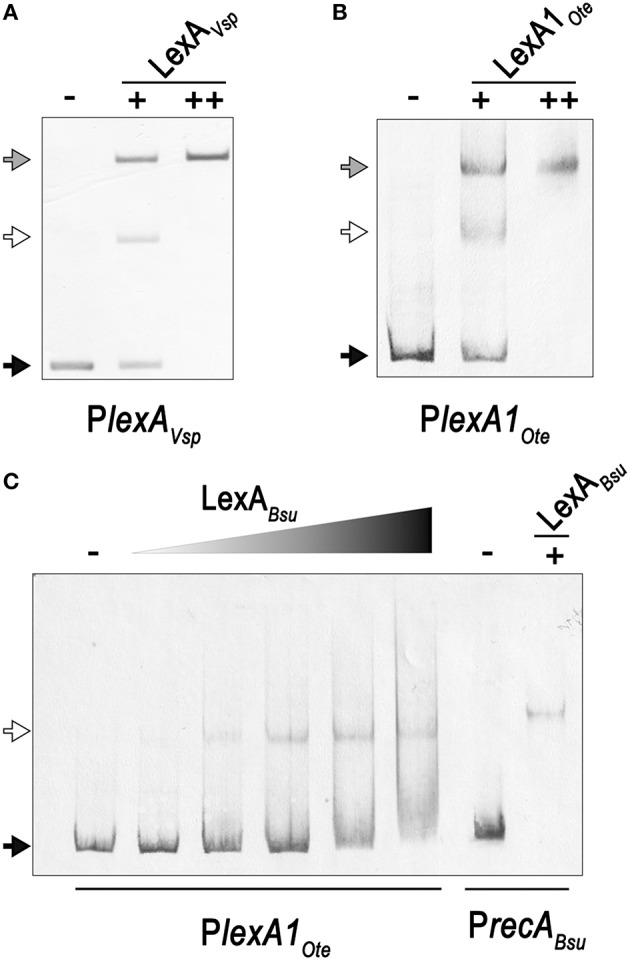
**(A)** Electro-mobility shift assays on the *V. spinosum lexA* (VSP_RS04780) promoter using increasing concentrations of purified *V. spinosum* LexA protein (WP_009959117). **(B)** Electro-mobility shift assays on the *O. terrae lexA* (OTER_RS20480) promoter using increasing concentrations of purified *O. terrae* LexA protein (WP_012376858). **(C)** Electro-mobility shift assays on the *O. terrae lexA* (OTER_RS20480) promoter using increasing concentrations (80, 120, 160, 200, 400 nM) of the *B.subtilis* LexA protein (WP_003238209). A *B. subtilis recA* (BSU16940) promoter probe was used as a positive binding control. In all panels, the “−” symbol denotes absence of protein, “+” and “++” the addition of 80 or 400 nM, respectively, of the corresponding LexA in the binding mixture. A black arrow designates unbound DNA, a white arrow indicates the retardation band created by LexA binding DNA a single LexA-binding site, and a gray arrow denotes the retardation band generated by LexA binding two LexA-binding sites.

Close inspection of the *V. spinosum lexA* promoter reveals a poorly conserved LexA-binding site immediately downstream (1 bp) of the putative LexA-binding site identified *in silico* (Figure 3). To confirm that both these putative motif instances are involved in LexA binding, we performed EMSA with purified *V. spinosum* LexA protein on the *lexA* promoter. The results shown in Figure 4A revealed the distinct formation of two retardation bands on the *lexA* promoter at low protein concentrations, corresponding to LexA binding at either one or the two LexA-binding sites identified in the *lexA* promoter. Further, increasing protein concentration resulted in a single retardation band corresponding to LexA recognizing both LexA-binding sites. Taken together, these results indicate that the two identified LexA-binding sites in the promoter region of the *V. spinosum lexA* gene are bound cooperatively by LexA. A systematic analysis of the promoter regions of *V. spinosum lexA* gene orthologs in the *Verrucomicrobia* revealed that more than half of the *lexA* ortholog promoters with predicted LexA-binding sites display similar tandem site configurations (Supplementary Material 5). Most of these tandem arrangements involve a conserved TGTTC-N4-GAACA motif instance followed by a degenerate site in which only the first TGTTC element is conserved, but a tandem site arrangement with both conserved sites can be observed in at least two species (Figure 3).

In *Opitutus terrae* PB90-1, there are two fully conserved *Verrucomicrobia* LexA-binding motifs in the promoter region of a putative *lexA-imuA-imuB-dnaE2* operon [OTER_RS20480-OTER_RS20475-OTER_RS20470-OTER_RS20465] separated by 2 bp. This arrangement generates an instance of the canonical GAAC-N4-GTTC LexA-binding motif of *Firmicutes* and *Actinobacteria*. Using purified *O. terrae* and *B. subtilis* LexA proteins, we performed EMSA to validate the functionality of this tandem arrangement in *O. terrae* (Figure 4). EMSA with *O. terrae* LexA [WP_012376858] reveals two retardation bands at low protein concentration, confirming that this protein also binds to both *Verrucomicrobia* LexA target sites in the *lexA-imuA-imuB-dnaE2* promoter (Figure 4B). Mobility assays with incremental concentrations of *B. subtilis* LexA [WP_003238209] show that *B. subtilis* LexA binds a unique element in the *O. terrae lexA-imuA-imuB-dnaE2* promoter, yielding a single retardation band similar to the one observed on the *B. subtilis recA* [BSU16940] promoter (Figure 4C). These results suggest that *B. subtilis* LexA binds the *Firmicutes*-like LexA-binding motif instance generated by the tandem arrangement of *Verrucomicrobia* LexA-binding sites.

### The core *Verrucomicrobia* LexA regulon comprises three operons involved in DNA repair and mutagenesis

**Figure 5:**
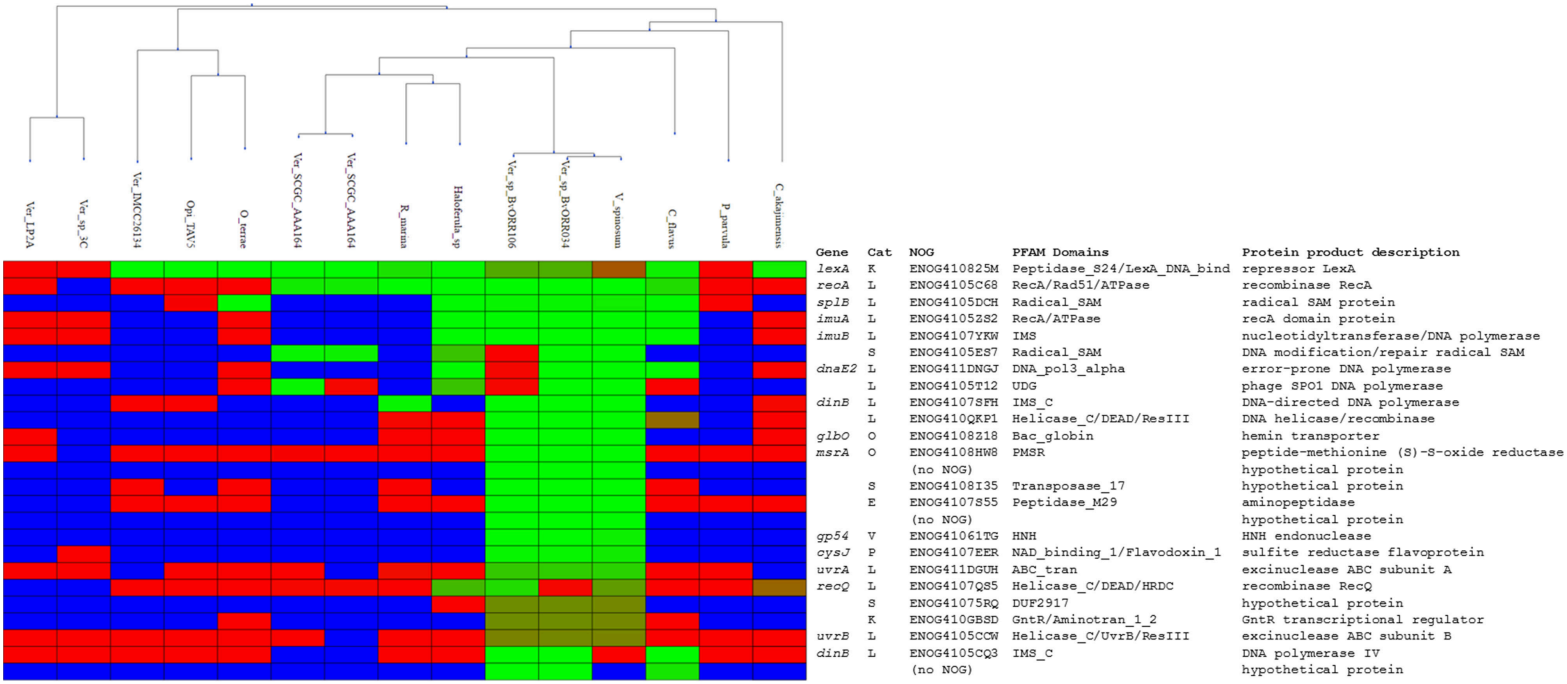
**Heatmap rendering of the comparative genomics analysis results**. Organisms (columns) are clustered according the phylogenetic proximity estimated from a LexA protein multiple sequence alignment. Each row corresponds to an ortholog group, with the commonly-used gene name, the eggNOG category, identifier, conserved PFAM domains, and protein product description shown on the right. For each species and orthologous group, evidence of regulation is shown in green-red shades. Light green shading indicates promoters associated with the ortholog with sites close or equal to the maximum site score detected in the analysis. Red shading denotes promoters in which the best scoring site has a score close or equal to the minimum score observed in the analysis. Blue shading indicates that no orthologous sequences have been identified in a given species for a particular ortholog group.

Having established the LexA-binding motif of the *Verrucomicrobia*, we performed a comparative genomics analysis of the LexA regulon in this phylum. We compiled 15 whole-genome shotgun assemblies for members of all the major classes of *Verrucomicrobia* (*Opitutae, Spartobacteria*, and *Verrucomicrobiae*) presenting a *V. spinosum* LexA homolog and searched for putative LexA-binding sites in the promoter region [−250, +50] of predicted operons. The results of the comparative genomics analysis (Figure 5; Supplementary Materials 6, 7) reveal a core LexA regulon present in all classes of the *Verrucromicrobia* phylum and composed of three operons: *lexA, splB*, and *imuA-imuB-dnaE2*. The *lexA* gene displays high-scoring sites in all the analyzed species, except for *Verrucomicrobium* sp. 3C (TaxID: 1134055), *Verrucomicrobia bacterium* LP2A (TaxID: 478741), and *Pedosphaera parvula* Ellin514 (TaxID: 320771). The LexA proteins of these species display significant changes to the α3 helix of the LexA DNA-binding domain, suggesting that they may target a divergent LexA-binding motif. Consistent with this result, the genomes of these organisms do not reveal any instance of the *Verrucomicrobia* LexA-binding motif in the promoter regions of previously documented SOS genes (Erill et al., 2007). The promoter region of the *splB* gene shows evidence of LexA regulation in several *Verrucomicrobiae*, one *Opitutaceae* (*O. terrae*) and the only available assembly of a *Spartobacteria* species (*Chthoniobacter flavus* Ellin428; TaxID: 497964). The product of the *splB* gene contains a radical SAM domain (PFAM04055) and has homology to COG1533 (ENOG4105DCH), classified as a DNA repair photolyase. Members of this orthologous group have been reported to be regulated by LexA in the *Actinobacteria*, the *Gammaproteobacteria*, the *Betaproteobacteria*, and the *Alphaproteobacteria* (Davis et al., 2002; Cirz et al., 2006; Sanchez-Alberola et al., 2012, 2015; Ulrich et al., 2013), suggesting that it may be a previously unrecognized core component of the SOS response. Lastly, the promoter region of the *imuA-imuB-dnaE2* operon presents *Verrucomicrobia* LexA-binding motif instances in *C. flavus* and the same *Verrucomicrobiae* species as *splB*. As noted above, *O. terrae* presents a putative *lexA-imuA-imuB-dnaE2* with verified *O. terrae* LexA binding in its promoter region (Figure 4). Even though the intergenic distance between *lexA* and *imuA* is larger than the genomic average for this species (264 bp), the prevalence of *lexA-imuA-imuB-dnaE2* arrangements across the *Bacteria* domain suggests that this direction constitutes a functional operon in *O. terrae* (Erill et al., 2006).

### The *Verrucomicrobia* LexA regulon is highly variable and incorporates novel functions

The results of the comparative genomics analysis reveal remarkable variation in the size and composition of the inferred LexA regulon. In the *Verrucomicrobia*e, the predicted regulon ranges from one operon (*Verrucomicrobia bacterium* IMCC26134; TaxID: 1637999) to over 14 (*V. spinosum*), with several species displaying intermediate sizes [2 operons in *Rubritalea marina* DSM 17716 (TaxID: 1123070) or 5 in *Haloferula* sp. BvORR071 (TaxID: 1396141)]. The only available Spartobacteria representative (*C. flavus*) shows a moderate regulon size (5 operons). In contrast, the LexA regulon appears to have shrunk noticeably in the *Opitutaceae*, where it encompasses at the most two operons. Two members of this family [*O. terrae* and *Opitutaceae bacterium* TAV5 (TaxID: 794903)] present a duplication of the *lexA* gene. The products of the two *Opitutaceae* TAV5 *lexA* genes (OPIT5_RS22040 and OPIT5_RS25725) present 91% identity and their promoter regions contain almost identical LexA-binding sites. The *lexA* genes in *O. terrae* (OTER_RS20480 and OTER_RS11645) have diverged substantially (42% protein sequence identity) and only the promoter of the *lexA1* gene (OTER_RS20480) presents *Verrucomicrobia* LexA-binding motif instances, following the tandem arrangement discussed above (Figure 3).

The overall composition of the inferred *Verrucomicrobia* LexA regulon is in broad agreement with experimental and computational descriptions of the SOS regulatory network in other phyla (Erill et al., 2007; Sanchez-Alberola et al., 2012, 2015). Beyond the core regulon described above (*lexA, splB, imuA-imuB-dnaE2*), the *Verrucomicrobia* LexA regulon encompasses genes coding for the recombination protein RecA (COG0468; ENOG4105C68), the excinuclease ABC subunits A (COG0178; ENOG411DGUH) and B (COG0556; ENOG4105CCW), two DNA helicase RecQ homologs (COG0514; ENOG4107QS5 and ENOG410QKP1) and two homologs of the error-prone DNA polymerase IV (COG0389; ENOG4105CCW and ENOG4105CQ3). In addition to these previously established SOS genes, the *Verrucomicrobia* LexA regulon shows evidence of regulation for an operon encoding proteins matching the TIGR03916 (ENOG4105ES7) and TIGR03915 (ENOG4105T12) models. These models are present in about 20% of sequenced bacterial genomes, arranged always in operon configuration, and are thought to constitute a DNA base excision repair system involving a uracil-DNA glycosylase (UDG) domain that is conserved in all *Verrucomicrobia* TIGR03915-matching homologs.

**Figure 6:**
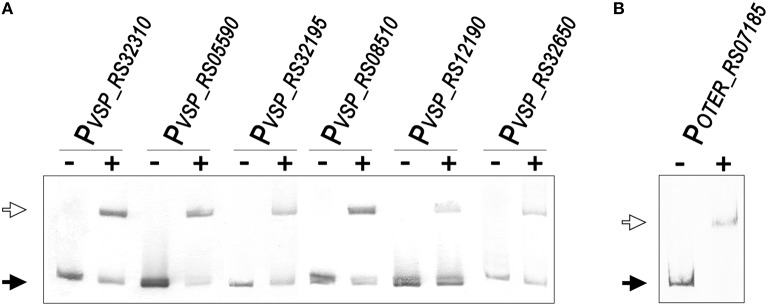
**(A)** Electro-mobility shift assays using purified *V. spinosum* LexA protein (WP_009959117) on the promoter region of predicted LexA-regulated genes. **(B)** Electro-mobility shift assays on the *O. terrae splB* promoter (OTER_RS07185) using purified *O. terrae* LexA protein (WP_012376858). On both panels, the “−” and “+” symbols denote the absence or presence (80 nM), respectively, of the corresponding protein. A black arrow designates unbound DNA and a white arrow indicates the retardation band created by LexA binding a single LexA-binding site.

To validate the predictions of the comparative genomics approach and further establish the LexA regulon of the *Verrucomicrobia*, we performed EMSA with purified *V. spinosum* and *O. terrae* proteins on the promoter region of several genes with predicted LexA-binding sites in these organisms and evidence of regulation in at least three different genomes. The results, shown in Figure 6, confirm that LexA binds to the promoter region of the *splB* gene in *O. terrae* (OTER_RS07185) and *V. spinosum* (VSP_RS12190). *V. spinosum* LexA also binds the *imuA-imuB-dnaE2* operon promoter, the promoters of genes coding for DNA polymerase IV (VSP_RS08510) and RecQ (VSP_RS32195) homologs, and the *recA* (VSP_RS04780) and *uvrA* (VSP_RS32650) promoters. Together with the comparative genomics analysis, these results confirm the existence of a conserved core LexA-regulon in the *Verrucomicrobia* and demonstrate that, in some *Verrucomicrobia* species, LexA controls a network of similar size and function to those reported in well-studied bacterial phyla, using a novel LexA-binding motif.

## Discussion

This work reports the combined use of *in silico* and *in vitro* techniques to characterize a novel binding motif for the SOS transcriptional repressor LexA in the *Verrucomicrobia*, and its use to define the LexA regulon in this bacterial phylum of emerging interest. The results provide further context to illustrate the complex evolutionary history of the SOS response, and put to the fore the plasticity and versatility of this transcriptional system.

### Variability and core elements of the SOS regulatory network

Phylogenetic and protein signature analyses have firmly established the *Verrucomicrobia* as one of the major phyla in the PVC super-phylum, an ancient bacterial group estimated to have diverged from other bacterial clades almost two billion years ago (Gupta et al., 2012; Kamneva et al., 2012; Lagkouvardos et al., 2014). The analysis of the LexA regulon performed in this work hence provides for the first time insights into the organization of a complex transcriptional system in this large bacterial clade. Our results show evidence of a functional LexA protein targeting the same LexA-binding motif in all three major *Verrucomicrobia* classes (*Opitutae, Spartobacteria*, and *Verrucomicrobia*e), suggesting that a functional LexA regulatory network was present in the ancestor of the *Verrucomicrobia* (Figure 1). However, the *Verrucomicrobia* also display substantial heterogeneity in the size of their predicted LexA regulons (Figure 5). Some families, such as the *Methylacidiphilaceae* do not present LexA homologs, while many members of the Verrucomicrobiales display small (1–3 operon) regulons, a setup that appears to be the rule in the Opitutales. Small SOS regulatory networks have been experimentally reported for several species, but mostly in association with drastic changes in the LexA-binding motif (Jara et al., 2003; Campoy et al., 2005; Mazon et al., 2006). In these instances, the LexA regulon is typically constrained to the regulation of translesion synthesis polymerases (Erill et al., 2007). Conversely, moderately large (10–40 genes) LexA regulons incorporating several DNA repair pathways have been documented in the *Gammaproteobacteria*, the *Betaproteobacteria*, the *Actinobacteria*, and the *Firmicutes* (Fernandez De Henestrosa et al., 2000; Davis et al., 2002; Au et al., 2005; Ulrich et al., 2013; Sanchez-Alberola et al., 2015). These findings have substantiated the notion that translesion synthesis is the primordial function of the SOS response, and the identification of a translesion synthesis operon (*imuA-imuB-dnaE2*) in the core LexA regulon of the *Verrucomicrobia* confirms the ancestral role of this mechanism in the SOS response. Nonetheless, the presence of a putative photolyase (*splB*) in the core *Verrucomicrobia* LexA regulon, with documented LexA-regulated orthologs in several bacterial clades (Davis et al., 2002; Cirz et al., 2006; Sanchez-Alberola et al., 2012, 2015; Ulrich et al., 2013), suggests that photoreactivation might have played an essential DNA repair role in the primordial SOS response.

Beyond the presence of a putative photolyase, the SOS response of the *Verrucomicrobia* presents several interesting differences with the canonical SOS response of *E. coli* and *B. subtilis*. The *Opitutae*, for instance, show a consistent absence of LexA regulation for the *recA* gene. The lack of *recA* regulation by LexA has been reported in several bacterial groups, such as the *Acidobacteria* and the *Deltaproteobacteria* (Jara et al., 2003; Campoy et al., 2005; Mazon et al., 2006). Loss of *recA* regulation is often associated with small LexA regulons and *lexA* gene duplication, which are both features of the *Opitutae* LexA network inferred in this work. Another distinct feature of the *Verrucomicrobia* LexA regulon is the regulation of multiple RecQ homologs (ENOG4107QS5 and ENOG410QKP1; Figure 5). One of these RecQ homologs (ENOG4107QS5) shares functional domains with *B. subtilis* RecS and RecQ proteins and therefore likely fulfills similar repair functions. The other RecQ homolog (ENOG410QKP1) lacks DNA-binding HRDC (Helicase and RNase D C-terminal) and RecQ-C-terminal (RQC) domains and presents weaker evidence of homology with *B. subtilis* RecS and RecQ proteins (Fernández et al., 1998). RecQ helicases are involved in the initiation and reversal of recombination and are known to act in concert with the product of SOS genes (*recA, ssb*), and to facilitate the onset of the SOS response (Heyer, 2004; Nakayama, 2005). Although SOS regulation of RecQ homologs has not been documented to date, LexA regulation of other DNA helicases (UvrD, PcrA, DinG) is a well-established feature of the SOS response in several organisms (Fernandez De Henestrosa et al., 2000; Au et al., 2005; Abella et al., 2007). These helicases do not appear to be regulated in the *Verrucomicrobia*, suggesting that the putative LexA regulation of RecQ homologs might be fulfilling a complementary role in this phylum. Our analysis also provides evidence of LexA regulation for an operon encoding radical SAM and uracil-DNA glycosylase domain-containing proteins (ENOG4105ES7 and ENOG4105T12; Figure 5), presumed to function as a DNA base excision repair system. SOS-regulated error-prone polymerases have been shown to have poor sugar discrimination, leading to the frequent misincorporation of ribonucleotides (Schroeder et al., 2015). Misincorporated ribonucleotides are usually removed by RNase HII-mediated ribonucleotide excision repair, but SOS-regulated nucleotide excision repair has also been shown to address ribonucleotide incorporation (Vaisman et al., 2013). The presence of putative LexA-regulated translesion synthesis polymerases in the *Verrucomicrobia* (Figures 5, 6) hence suggests that the regulation of this base excision repair operon by LexA may play a role in addressing uracil misincorporation resulting from SOS induction in this phylum.

### A tandem model for the evolution of the LexA-binding motif

**Figure 7:**
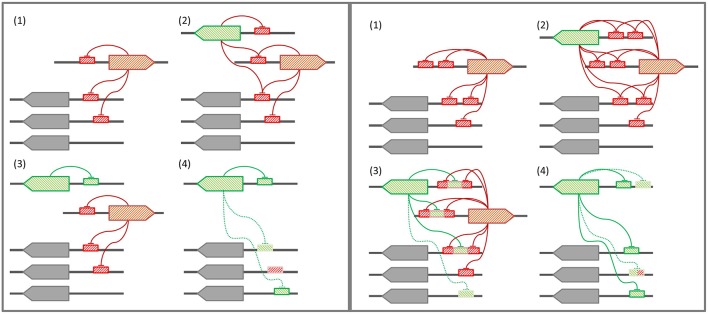
**(Left panel) Conventional model for LexA-binding motif evolution. (1)** The regulon is under control of the primary LexA, which represses itself and other genes. **(2)** A *lexA* gene duplication takes place. **(3)** The secondary LexA protein diverges, targeting a novel LexA-binding motif for self-regulation. **(4)** Upon deletion of the primary LexA, convergent evolution drives the uptake of the former regulon by the secondary LexA. **(Right panel)** Tandem site-based model for LexA-binding motif evolution. **(1)** The regulon is under control of the primary LexA, which represses itself via tandem LexA-binding sites. **(2)** A *lexA* gene duplication takes place. **(3)** The secondary LexA protein diverges, targeting the sites created by the tandem site arrangement in the promoter region of primary LexA target genes**. (4)** Upon deletion of the primary LexA, the secondary LexA is already in control of the core regulon, and leverages half-site affinity in remaining regulon genes to take over control of the former regulon.

In those bacterial phyla where the SOS response has been experimentally documented, the LexA-binding motif shows evidence of high conservation, punctuated by periods of rapid divergence and further stabilization (Erill et al., 2007). In the *Firmicutes* and the *Actinobacteria*, LexA targets a conserved GAAC-N4-GTTC LexA-binding motif that is monophyletic for both clades (Cornish et al., 2012), and variations of this motif are also seen in other bacterial groups, such as the *Cyanobacteria* or the Chloroflexi (Fernandez de Henestrosa et al., 2002; Mazon et al., 2004b). In the *Proteobacteria*, however, LexA shows an extraordinary diversity of binding motifs (Erill et al., 2007). In *Proteobacteria* classes with abundant sequence information (*Alphaproteobacteria, Betaproteobacteria*, and *Gammaproteobacteria*), the LexA-binding motif has been found to be extremely well-conserved, but exceptions to the canonical LexA-binding motif of *Gammaproteobacteria* and *Betaproteobacteria* have been reported in several subgroups (Campoy et al., 2002; Abella et al., 2007; Sanchez-Alberola et al., 2015). These exceptions are associated with duplications of the *lexA* gene, suggesting a model for LexA-binding motif evolution (Figure 7) in which *lexA* duplication leads to progressive divergence in the LexA-binding motif of the duplicated *lexA*, until the primary *lexA* gene is deleted and the divergent LexA takes control of the regulon (Abella et al., 2007; Yang et al., 2008; Sanchez-Alberola et al., 2015). While this model provides a causal mechanism for LexA-motif divergence, it does not address how a divergent LexA can swiftly take control over a regulatory network defined, up to the deletion event, by LexA-binding sites matching the primary LexA-binding motif. Furthermore, the model does not provide a mechanistic explanation for the recurrence of very similar LexA-binding motifs in distantly related bacterial clades, such as the *Firmicutes* and the Gallionellales, recognized through seemingly unrelated LexA α3 helices (Sanchez-Alberola et al., 2015).

Many bacterial transcription factors bind cooperatively to tandem sites (Barnard et al., 2004). The existence of tandem sites for LexA was first reported in the promoter region of the *E. coli lexA* gene (Brent, 1982) and then shown to be a common feature of *lexA* genes in the *Gammaproteobacteria* (Garriga et al., 1992), the *Betaproteobacteria*, and *Alphaproteobacteria* (Sanchez-Alberola et al., 2012), and the *Firmicutes* and *Actinobacteria* (Cornish et al., 2012). These arrangements feature highly conserved and spatially close tandem sites (1–10 bp apart). Tandem LexA-binding sites have also been experimentally reported for other SOS genes, such as the *ydjM* gene of *E. coli* (Fernandez De Henestrosa et al., 2000) or the *umuDC-*like operon (*yqjW-yqzH*) of *B. subtilis* (Au et al., 2005). Furthermore, the use of cooperative LexA-binding to enhance repression has been experimentally demonstrated for several colicin genes, which display a tandem arrangement with a strong and a weak LexA-binding site overlapping at their terminal positions (Gillor et al., 2008). In the *Verrucomicrobia*, there is evidence of a recent *lexA* duplication in the *Opitutaceae* and tandem LexA-binding sites separated by short distances appear to be a conserved feature of the *lexA* promoter (Figure 3). The ability of the *Verrucomicrobia* LexA to cooperatively bind degenerate sites and the fact that at least in one of these species the tandem arrangement generates a functional *B. subtilis* LexA-binding motif (Figure 4) suggest that tandem site arrangements can yield a simple mechanistic process for the evolution of LexA-binding motifs.

In the tandem site model (Figure 7), LexA binds consecutive sites in its own promoter and in the promoter of key SOS genes that need to be tightly regulated (Gillor et al., 2008). Upon *lexA* duplication, the site generated by the tandem arrangement provides the secondary LexA with a conserved target for motif divergence. This allows the secondary LexA to maintain cross-regulation with the primary LexA and a subset of its regulon, while incorporating novel elements to its network. After deletion of the primary *lexA* gene, the secondary LexA is hence already in control of a core LexA regulon, and can rapidly evolve sites on other target genes by exploiting its partial overlap, and presumable weak binding affinity, with primary *lexA* sites. The tandem site model therefore provides a conservative mechanism for the evolution of LexA-binding motifs that is capable of addressing outstanding questions regarding the complex evolutionary history of the SOS response. On the one hand, the conservative nature of the model provides a natural explanation for the persistence of conserved SOS response networks under divergent LexA-binding motifs, without the need for a strong selective process driving convergent evolution of similar networks (Sanchez-Alberola et al., 2015). On the other hand, the implicit reuse of LexA monomer-binding sites in the tandem model helps explain the observation of many LexA-binding motifs involving the rearrangement of similar monomer binding sites on different motif structures (Mazon et al., 2004a; Sanchez-Alberola et al., 2015).

Several lines of evidence provide indirect support for a tandem site-based model of LexA-binding motif evolution. As mentioned above, the prevalence of such arrangements in the promoter of *lexA* and other SOS genes has been documented in several bacterial clades. Furthermore, *lexA* duplications targeting identical and divergent motifs have also been experimentally reported, and cross-regulation between duplicated *lexA* genes has been demonstrated in these systems (Jara et al., 2003; Abella et al., 2007; Yang et al., 2008). Lastly, previous work has shown that LexA can bind to degenerate sites that partially match other LexA-binding motifs, indicating that transitional stages of LexA divergence in which the secondary LexA could partially bind the original and tandem-generated motifs are possible (Mazon et al., 2004a). Due to its broad distribution in several phyla, the *Firmicutes* and *Actinobacteria* LexA-binding motif has long been assumed to represent the ancestral motif of LexA. The mirror image relationship between *Firmicutes* and *Verrucomicrobia* LexA-binding motifs, and the generation of functional *B. subtilis* LexA-binding sites from tandem *Verrucomicrobia* LexA-binding sites, hence suggest that the *Verrucomicrobia* LexA-binding motif might have originated after the duplication of a *lexA* gene targeting a tandem arrangement of *Firmicutes*-like LexA-binding sites in a common ancestor of these lineages. The analysis of the α3 helix of the *Verrucomicrobia* LexA DNA-binding domain (Figure 2) supports this hypothesis, revealing overall conservation of amino acid properties and a substitution pattern consistent with changes in the specific readout of monomer sites, but not in overall motif recognition, as expected in the tandem site evolution model.

## Conclusions

By combining *in silico* and *in vitro* methods, in this work we have characterized a novel LexA-binding motif in the *Verrucomicrobia*. Using this motif, which presents structural similarities with LexA-binding motifs previously described in other phyla, we performed a comparative genomics analysis of the LexA regulon in this understudied phylum. Our computational analysis, validated through *in vitro* assays, revealed significant variability in the size and composition of the LexA regulatory network of this phylum, and identified novel core and ancillary components of the SOS response. The characterization of the *Verrucomicrobia* LexA-binding motif and regulon also allowed us to postulate for the first time a model for LexA-binding motif evolution that satisfactorily addresses open questions in the evolution of this system via gene duplication events. Future biochemical and genetic experiments, such as determining the conformation of LexA in solution and analyzing expression patterns in mutants for core SOS genes, should provide a more comprehensive characterization of the *Verrucomicrobia* SOS response and its evolution.

## Author contributions

IE and SK performed the *in silico* analyses. SK developed scripts for comparative genomics. SC performed the *in vitro* analyses. IE, SC, and JB discussed the findings and interpreted the results. IE and JB conceived the experiment and coordinated the research. IE drafted the manuscript.

## Funding

This work was supported by Spanish Ministry of Science and Innovation (BFU2011-23478) and Generalitat de Catalunya (2014SGR572) awards to JB and by a U.S. National Science Foundation (MCB-1158056) award to IE.

### Conflict of interest statement

The authors declare that the research was conducted in the absence of any commercial or financial relationships that could be construed as a potential conflict of interest.
