# Supplementary material for: The Verrucomicrobia LexA-Binding Motif: Insights into the Evolutionary Dynamics of the SOS Response
Source: Front Mol Biosci. 2016 Jul 20;3:33. doi: 10.3389/fmolb.2016.00033 (PMC4951493; doi:10.3389/fmolb.2016.00033)
Supplement: Supplementary file 1 [file Table1.DOCX]

Supplementary Material

The Verrucomicrobia LexA-binding Motif: Insights into the Evolutionary Dynamics of the SOS Response

Ivan Erill^1^, Susana Campoy^2^, Sefa Kılıç^1^ and Jordi Barbé^2*^

*** Correspondence:** Jordi Barbé, jordi.barbe@uab.cat

Supplementary material 2 – Table S1 – Oligonucleotides used in this work. The table lists primers used to amplify *lexA* genes and to obtain all the promoter probes.

| **Oligonucleotides used in this work** | | |
| --- | --- | --- |
| **Name** | **Application** | **Sequence (5’→3’)** |
| NdelexAVsp | Upper primer for cloning the *V.spinosum* *lexA* gen in pET15b overexpression vector. | *CATATG*CTTACGGAGCGGCAAC |
| BamlexAVsp | Lower primer for cloning the *V.spinosum* *lexA* gen in pET15b overexpression vector | *GGATCC*CTACCGTCCGTTGTAG |
| VSRS32310F | Synthetic oligo to obtain the *V. spinosum recA* promoter EMSA probe. | CCTCCAGTGCAGAAACATTTCTCAAAATAGGAGTTGCAACTCCTGTTCAATCGAACATTATCGCTCTCAACTGTTCACCAAACTCTAATACCGACACCGA |
| VSRS32310R | Synthetic oligo to obtain the *V. spinosum recA* promoter EMSA probe. | CGGTGTCGGTATTAGAGTTTGGTGAACAGTTGAGAGCGATAATGTTCGATTGAACAGGAGTTGCAACTCCTATTTTGAGAAATGTTTCTGCACTGGAGGA |
| Mut60TrecV | Synthetic oligo to obtain a mutant *V. spinosum recA* promoter EMSA probe. | CCTCCAGTGCAGAAACATTTCTCAAAATAGGAGTTGCAACGCCTGTTCAATCGAACATTATCGCTCTCAACTGTTCACCAAACTCTAATACCGACACCGA |
| Mut60TrecV | Synthetic oligo to obtain a mutant *V. spinosum recA* promoter EMSA probe. | CGGTGTCGGTATTAGAGTTTGGTGAACAGTTGAGAGCGATAATGTTCGATTGAACAGGCGTTGCAACTCCTATTTTGAGAAATGTTTCTGCACTGGAGGA |
| Mut59CrecV | Synthetic oligo to obtain a mutant *V. spinosum recA* promoter EMSA probe. | CCTCCAGTGCAGAAACATTTCTCAAAATAGGAGTTGCAACTACTGTTCAATCGAACATTATCGCTCTCAACTGTTCACCAAACTCTAATACCGACACCGA |
| Mut59CrecV | Synthetic oligo to obtain a mutant *V. spinosum recA* promoter EMSA probe. | CGGTGTCGGTATTAGAGTTTGGTGAACAGTTGAGAGCGATAATGTTCGATTGAACAGTAGTTGCAACTCCTATTTTGAGAAATGTTTCTGCACTGGAGGA |
| Mut58ArecV | Synthetic oligo to obtain a mutant *V. spinosum recA* promoter EMSA probe. | CCTCCAGTGCAGAAACATTTCTCAAAATAGGAGTTGCAACTCATGTTCAATCGAACATTATCGCTCTCAACTGTTCACCAAACTCTAATACCGACACCGA |
| Mut58ArecV | Synthetic oligo to obtain a mutant *V. spinosum recA* promoter EMSA probe. | CGGTGTCGGTATTAGAGTTTGGTGAACAGTTGAGAGCGATAATGTTCGATTGAACATGAGTTGCAACTCCTATTTTGAGAAATGTTTCTGCACTGGAGGA |
| Mut57GrecV | Synthetic oligo to obtain a mutant *V. spinosum recA* promoter EMSA probe. | CCTCCAGTGCAGAAACATTTCTCAAAATAGGAGTTGCAACTCCGGTTCAATCGAACATTATCGCTCTCAACTGTTCACCAAACTCTAATACCGACACCGA |
| Mut57GrecV | Synthetic oligo to obtain a mutant *V. spinosum recA* promoter EMSA probe. | CGGTGTCGGTATTAGAGTTTGGTGAACAGTTGAGAGCGATAATGTTCGATTGAACCGGAGTTGCAACTCCTATTTTGAGAAATGTTTCTGCACTGGAGGA |
| Mut56TrecV | Synthetic oligo to obtain a mutant *V. spinosum recA* promoter EMSA probe. | CCTCCAGTGCAGAAACATTTCTCAAAATAGGAGTTGCAACTCCTTTTCAATCGAACATTATCGCTCTCAACTGTTCACCAAACTCTAATACCGACACCGA |
| Mut56TrecV | Synthetic oligo to obtain a mutant *V. spinosum recA* promoter EMSA probe. | CGGTGTCGGTATTAGAGTTTGGTGAACAGTTGAGAGCGATAATGTTCGATTGAAAAGGAGTTGCAACTCCTATTTTGAGAAATGTTTCTGCACTGGAGGA |
| Mut55GrecV | Synthetic oligo to obtain a mutant *V. spinosum recA* promoter EMSA probe. | CCTCCAGTGCAGAAACATTTCTCAAAATAGGAGTTGCAACTCCTGGTCAATCGAACATTATCGCTCTCAACTGTTCACCAAACTCTAATACCGACACCGA |
| Mut55GrecV | Synthetic oligo to obtain a mutant *V. spinosum recA* promoter EMSA probe. | CGGTGTCGGTATTAGAGTTTGGTGAACAGTTGAGAGCGATAATGTTCGATTGACCAGGAGTTGCAACTCCTATTTTGAGAAATGTTTCTGCACTGGAGGA |
| Mut54GrecV | Synthetic oligo to obtain a mutant *V. spinosum recA* promoter EMSA probe. | CCTCCAGTGCAGAAACATTTCTCAAAATAGGAGTTGCAACTCCTGTGCAATCGAACATTATCGCTCTCAACTGTTCACCAAACTCTAATACCGACACCGA |
| Mut54GrecV | Synthetic oligo to obtain a mutant *V. spinosum recA* promoter EMSA probe. | CGGTGTCGGTATTAGAGTTTGGTGAACAGTTGAGAGCGATAATGTTCGATTGCACAGGAGTTGCAACTCCTATTTTGAGAAATGTTTCTGCACTGGAGGA |
| Mut53ArecV | Synthetic oligo to obtain a mutant *V. spinosum recA* promoter EMSA probe. | CCTCCAGTGCAGAAACATTTCTCAAAATAGGAGTTGCAACTCCTGTTAAATCGAACATTATCGCTCTCAACTGTTCACCAAACTCTAATACCGACACCGA |
| Mut53ArecV | Synthetic oligo to obtain a mutant *V. spinosum recA* promoter EMSA probe. | CGGTGTCGGTATTAGAGTTTGGTGAACAGTTGAGAGCGATAATGTTCGATTTAACAGGAGTTGCAACTCCTATTTTGAGAAATGTTTCTGCACTGGAGGA |
| Mut52CrecV | Synthetic oligo to obtain a mutant *V. spinosum recA* promoter EMSA probe. | CCTCCAGTGCAGAAACATTTCTCAAAATAGGAGTTGCAACTCCTGTTCCATCGAACATTATCGCTCTCAACTGTTCACCAAACTCTAATACCGACACCGA |
| Mut52CrecV | Synthetic oligo to obtain a mutant *V. spinosum recA* promoter EMSA probe. | CGGTGTCGGTATTAGAGTTTGGTGAACAGTTGAGAGCGATAATGTTCGATGGAACAGGAGTTGCAACTCCTATTTTGAGAAATGTTTCTGCACTGGAGGA |
| Mut51CrecV | Synthetic oligo to obtain a mutant *V. spinosum recA* promoter EMSA probe. | CCTCCAGTGCAGAAACATTTCTCAAAATAGGAGTTGCAACTCCTGTTCACTCGAACATTATCGCTCTCAACTGTTCACCAAACTCTAATACCGACACCGA |
| Mut51CrecV | Synthetic oligo to obtain a mutant *V. spinosum recA* promoter EMSA probe. | CGGTGTCGGTATTAGAGTTTGGTGAACAGTTGAGAGCGATAATGTTCGAGTGAACAGGAGTTGCAACTCCTATTTTGAGAAATGTTTCTGCACTGGAGGA |
| Mut50GrecV | Synthetic oligo to obtain a mutant *V. spinosum recA* promoter EMSA probe. | CCTCCAGTGCAGAAACATTTCTCAAAATAGGAGTTGCAACTCCTGTTCAAGCGAACATTATCGCTCTCAACTGTTCACCAAACTCTAATACCGACACCGA |
| Mut50GrecV | Synthetic oligo to obtain a mutant *V. spinosum recA* promoter EMSA probe. | CGGTGTCGGTATTAGAGTTTGGTGAACAGTTGAGAGCGATAATGTTCGCTTGAACAGGAGTTGCAACTCCTATTTTGAGAAATGTTTCTGCACTGGAGGA |
| Mut49ArecV | Synthetic oligo to obtain a mutant *V. spinosum recA* promoter EMSA probe. | CCTCCAGTGCAGAAACATTTCTCAAAATAGGAGTTGCAACTCCTGTTCAATAGAACATTATCGCTCTCAACTGTTCACCAAACTCTAATACCGACACCGA |
| Mut49ArecV | Synthetic oligo to obtain a mutant *V. spinosum recA* promoter EMSA probe. | CGGTGTCGGTATTAGAGTTTGGTGAACAGTTGAGAGCGATAATGTTCTATTGAACAGGAGTTGCAACTCCTATTTTGAGAAATGTTTCTGCACTGGAGGA |
| Mut48TrecV | Synthetic oligo to obtain a mutant *V. spinosum recA* promoter EMSA probe. | CCTCCAGTGCAGAAACATTTCTCAAAATAGGAGTTGCAACTCCTGTTCAATCTAACATTATCGCTCTCAACTGTTCACCAAACTCTAATACCGACACCGA |
| Mut48TrecV | Synthetic oligo to obtain a mutant *V. spinosum recA* promoter EMSA probe. | CGGTGTCGGTATTAGAGTTTGGTGAACAGTTGAGAGCGATAATGTTAGATTGAACAGGAGTTGCAACTCCTATTTTGAGAAATGTTTCTGCACTGGAGGA |
| Mut47CrecV | Synthetic oligo to obtain a mutant *V. spinosum recA* promoter EMSA probe. | CCTCCAGTGCAGAAACATTTCTCAAAATAGGAGTTGCAACTCCTGTTCAATCGCACATTATCGCTCTCAACTGTTCACCAAACTCTAATACCGACACCGA |
| Mut47CrecV | Synthetic oligo to obtain a mutant *V. spinosum recA* promoter EMSA probe. | CGGTGTCGGTATTAGAGTTTGGTGAACAGTTGAGAGCGATAATGTGCGATTGAACAGGAGTTGCAACTCCTATTTTGAGAAATGTTTCTGCACTGGAGGA |
| Mut46CrecV | Synthetic oligo to obtain a mutant *V. spinosum recA* promoter EMSA probe. | CCTCCAGTGCAGAAACATTTCTCAAAATAGGAGTTGCAACTCCTGTTCAATCGAACCATTATCGCTCTCAACTGTTCACCAAACTCTAATACCGACACCGA |
| Mut46CrecV | Synthetic oligo to obtain a mutant *V. spinosum recA* promoter EMSA probe. | CGGTGTCGGTATTAGAGTTTGGTGAACAGTTGAGAGCGATAATGGTCGATTGAACAGGAGTTGCAACTCCTATTTTGAGAAATGTTTCTGCACTGGAGGA |
| Mut45ArecV | Synthetic oligo to obtain a mutant *V. spinosum recA* promoter EMSA probe. | CCTCCAGTGCAGAAACATTTCTCAAAATAGGAGTTGCAACTCCTGTTCAATCGAAAATTATCGCTCTCAACTGTTCACCAAACTCTAATACCGACACCGA |
| Mut45ArecV | Synthetic oligo to obtain a mutant *V. spinosum recA* promoter EMSA probe. | CGGTGTCGGTATTAGAGTTTGGTGAACAGTTGAGAGCGATAATTTTCGATTGAACAGGAGTTGCAACTCCTATTTTGAGAAATGTTTCTGCACTGGAGGA |
| Mut44CrecV | Synthetic oligo to obtain a mutant *V. spinosum recA* promoter EMSA probe. | CCTCCAGTGCAGAAACATTTCTCAAAATAGGAGTTGCAACTCCTGTTCAATCGAACCTTATCGCTCTCAACTGTTCACCAAACTCTAATACCGACACCGA |
| Mut44CrecV | Synthetic oligo to obtain a mutant *V. spinosum recA* promoter EMSA probe. | CGGTGTCGGTATTAGAGTTTGGTGAACAGTTGAGAGCGATAAGGTTCGATTGAACAGGAGTTGCAACTCCTATTTTGAGAAATGTTTCTGCACTGGAGGA |
| Mut43ArecV | Synthetic oligo to obtain a mutant *V. spinosum recA* promoter EMSA probe. | CCTCCAGTGCAGAAACATTTCTCAAAATAGGAGTTGCAACTCTTGTTCAATCGAACAATATCGCTCTCAACTGTTCACCAAACTCTAATACCGACACCGA |
| Mut43ArecV | Synthetic oligo to obtain a mutant *V. spinosum recA* promoter EMSA probe. | CGGTGTCGGTATTAGAGTTTGGTGAACAGTTGAGAGCGATAATGTTCGATTGAACAGGAGTTGCAACTCCTATTTTGAGAAATGTTTCTGCACTGGAGGA |
| Mut42TrecV | Synthetic oligo to obtain a mutant *V. spinosum recA* promoter EMSA probe. | CCTCCAGTGCAGAAACATTTCTCAAAATAGGAGTTGCAACTCCTGTTCAATCGAACATGATCGCTCTCAACTGTTCACCAAACTCTAATACCGACACCGA |
| Mut42TrecV | Synthetic oligo to obtain a mutant *V. spinosum recA* promoter EMSA probe. | CGGTGTCGGTATTAGAGTTTGGTGAACAGTTGAGAGCGATCATGTTCGATTGAACAGGAGTTGCAACTCCTATTTTGAGAAATGTTTCTGCACTGGAGGA |
| Mut41TrecV | Synthetic oligo to obtain a mutant *V. spinosum recA* promoter EMSA probe. | CCTCCAGTGCAGAAACATTTCTCAAAATAGGAGTTGCAACTCCTGTTCAATCGAACATTTTCGCTCTCAACTGTTCACCAAACTCTAATACCGACACCGA |
| Mut41TrecV | Synthetic oligo to obtain a mutant *V. spinosum recA* promoter EMSA probe. | CGGTGTCGGTATTAGAGTTTGGTGAACAGTTGAGAGCGAAAATGTTCGATTGAACAGGAGTTGCAACTCCTATTTTGAGAAATGTTTCTGCACTGGAGGA |
| PLexAVspF | Synthetic oligo to obtain the *V. spinosum lexA* promoter EMSA probe. | agcagaaaacacgaatggccaaaaaactcttgactgcacaaccgaacagtgttcacatctaaccatgcttacggagcggcaacaagagctactggattTA |
| PLexAVspR | Synthetic oligo to obtain the *V. spinosum lexA* promoter EMSA probe. | AAATCCAGTAGCTCTTGTTGCCGCTCCGTAAGCATGGTTAGATGTGAACACTGTTCGGTTGTGCAGTCAAGAGTTTTTTGGCCATTCGTGTTTTCTGCTA |
| LexA1OteSF | Synthetic oligo to obtain the *O.terrae lexA1* promoter EMSA probe. | TCGTCAGCCGGAGCTTGCCAAGCGGGCGGCCCGGCTGTTCGCTTGAACATGTGTTCAAGAGAACACATCCGCCATGCTCACCGAAAAACAGGAAGCCATA |
| LexA1OteSR | Synthetic oligo to obtain the *O.terrae lexA1* promoter EMSA probe. | ATGGCTTCCTGTTTTTCGGTGAGCATGGCGGATGTGTTCTCTTGAACACATGTTCAAGCGAACAGCCGGGCCGCCCGCTTGGCAAGCTCCGGCTGACGAA |
| VSRS32195F | Synthetic oligo to obtain the VSP_RS32195 promoter EMSA probe. | gagccagacaaacttgtggacaattccactcttcgcatcgatctgttcttttgaacacaggaagaaaaaacctcgatggtggacacattctatacccggA |
| VSRS32195R | Synthetic oligo to obtain the VSP_RS32195 promoter EMSA probe. | ccgggtatagaatgtgtccaccatcgaggttttttcttcctgtgttcaaaagaacagatcgatgcgaagagtggaattgtccacaagtttgtctggctcA |
| VSRS05590F | Synthetic oligo to obtain the *VSP_RS05590* promoter EMSA probe. | ATCCTTCCCGGGCTTGCCAAAGGGCATCTTTTAAAGAAAATAGTGTTCATGTGAACACTTTGCTTTTGCCTGCCAATGTCATCGCCTTCCGGGAACTCTA |
| VSRS05590R | Synthetic oligo to obtain the *VSP_RS05590* promoter EMSA probe. | AGAGTTCCCGGAAGGCGATGACATTGGCAGGCAAAAGCAAAGTGTTCACATGAACACTATTTTCTTTAAAAGATGCCCTTTGGCAAGCCCGGGAAGGATA |
| VSRS08510F | Synthetic oligo to obtain the *VSP_RS08510* promoter EMSA probe. | ttataaagggacagacactttattgatgcaaatgcaattttaaatgttcttgtgaacagtattatttagaatgggaccggaccttattcttgatctggaA |
| VSRS08510R | Synthetic oligo to obtain the *VSP_RS08510* promoter EMSA probe. | tccagatcaagaataaggtccggtcccattctaaataatactgttcacaagaacatttaaaattgcatttgcatcaataaagtgtctgtccctttataaA |
| VSRS12190F | Synthetic oligo to obtain the *VSP_RS12190* promoter EMSA probe. | caaaaccctcgccaatgcaggtggcagttataggtttgacgagtgttcaaatgaactgtaaccttggcgcatgaacgaagccgctcacgcgcagataacA |
| VSRS12190R | Synthetic oligo to obtain the *VSP_RS12190* promoter EMSA probe. | gttatctgcgcgtgagcggcttcgttcatgcgccaaggttacagttcatttgaacactcgtcaaacctataactgccacctgcattggcgagggttttgA |
| VSRS32650F | Synthetic oligo to obtain the *VSP_RS32650* promoter EMSA probe. | ctccccttgcgccgccgccaatcccggctaaaggcaccacaggtggtcaaataaacaccagtccgatgcacccgaccgccgctggcgcacgaatcggatA |
| VSRS32650R | Synthetic oligo to obtain the *VSP_RS32650* promoter EMSA probe. | ATCCGATTCGTGCGCCAGCGGCGGTCGGGTGCATCGGACTGGTGTTTATTTGACCACCTGTGGTGCCTTTAGCCGGGATTGGCGGCGGCGCAAGGGGAGA |
| OTRS07185F | Synthetic oligo to obtain the *OTER_RS07185* promoter EMSA probe. | catcgccggatcgacttgcacccgccgcgaaacgactcacctgtgttcaggtgaacacatgtggcgcaactccccctattggccgcggcgccaacattaA |
| OTRS07185R | Synthetic oligo to obtain the *OTER_RS07185* promoter EMSA probe. | taatgttggcgccgcggccaatagggggagttgcgccacatgtgttcacctgaacacaggtgagtcgtttcgcggcgggtgcaagtcgatccggcgatgA |
| PrecABsuF | Synthetic oligo to obtain the *B.subtilis recA* promoter EMSA probe. | ATACATTATGATATTTTGATAGGAATCACGCCAAGAAAAAATCCGAATATGCGTTCGCTTTTTTCTTGGCAAATCCCTTCAAACAGGGTATAGTATATGA |
| PrecABsuR | Synthetic oligo to obtain the *B.subtilis recA* promoter EMSA probe. | CATATACTATACCCTGTTTGAAGGGATTTGCCAAGAAAAAAGCGAACGCATATTCGGATTTTTTCTTGGCGTGATTCCTATCAAAATATCATAATGTATA |
| M13F/pUC | Universal upper primer of pGEMT vector to obtain the EMSA probe labeled with digoxigenin (DIG). | DIG/gttttcccagtcacgac |
| M13R/pUC | Universal lower primer of pGEMT vector to obtain the EMSA probe labeled with digoxigenin (DIG). | DIG/caggaaacagctatgac |
