# Supplementary material for: The Verrucomicrobia LexA-Binding Motif: Insights into the Evolutionary Dynamics of the SOS Response
Source: Front Mol Biosci. 2016 Jul 20;3:33. doi: 10.3389/fmolb.2016.00033 (PMC4951493; doi:10.3389/fmolb.2016.00033)
Supplement: Supplementary file 2 [file Table2.DOCX]

Supplementary Material

The Verrucomicrobia LexA-binding Motif: Insights into the Evolutionary Dynamics of the SOS Response

Ivan Erill^1^, Susana Campoy^2^, Sefa Kılıç^1^ and Jordi Barbé^2*^

*** Correspondence:** Jordi Barbé, jordi.barbe@uab.cat

Supplementary material 2 – Table S2 – List of 118 Verrucomicrobia *lexA*, *recA* and *imuA* genes used for motif discovery with MEME. For each gene, the table reports the JGI Gene ID and locus tag, the gene product description, the gene name and strand, and the Verrucomicrobia species to which it belongs.

| **Gene ID (JGI)** | **Locus Tag (JGI)** | **Product** | **Name** | **Strand** | **Species** |
| --- | --- | --- | --- | --- | --- |
| 2236287223 | A300N18DRAFT_NODE-unique_3_len_67233.3 | recombination protein RecA | *recA* | - | Verrucomicrobia SCGC AAA300-N18 |
| 2236429485 | A164E4DRAFT_NODE-unique_1_len_319654.1 | recombination protein RecA | *recA* | + | Verrucomicrobia SCGC AAA164-E04 |
| 2236430230 | A164E4DRAFT_NODE-unique_4_len_173494.4 | repressor LexA | *lexA* | + | Verrucomicrobia SCGC AAA164-E04 |
| 2236434052 | A168E21DRAFT_NODE-unique_18_len_31805.18 | SOS regulatory protein LexA | *lexA* | + | Verrucomicrobia SCGC AAA168-E21 |
| 2236435003 | A168E21DRAFT_NODE-unique_83_len_6729.83 | protein RecA | *recA* | - | Verrucomicrobia SCGC AAA168-E21 |
| 2236453899 | A168F10DRAFT_NODE-unique_20_len_54947.20 | protein RecA | *recA* | + | Verrucomicrobia SCGC AAA168-F10 |
| 2236454024 | A168F10DRAFT_NODE-unique_23_len_53784.23 | SOS regulatory protein LexA | *lexA* | - | Verrucomicrobia SCGC AAA168-F10 |
| 2236679956 | IBNHDRAFT_NODE-unique_3_len_71591.3 | recombination protein RecA | *recA* | - | Verrucomicrobia bacterium SCGC AAA300-K03 |
| 2236682069 | IBNIDRAFT_NODE-unique_96_len_1312.96 | RecA/RadA recombinase | *recA* | + | Verrucomicrobia bacterium SCGC AAA300-O17 |
| 2236682078 | IBNIDRAFT_NODE-unique_100_len_1231.100 | protein RecA | *recA* | + | Verrucomicrobia bacterium SCGC AAA300-O17 |
| 2508835133 | OpiT1DRAFT_OBC.1 | recombination protein RecA | *recA* | + | Opitutaceae sp. TAV1 |
| 2510264541 | Opit5_Contig145.1 | recombination protein RecA | *recA* | - | Opitutaceae sp. TAV5 |
| 2510268627 | Opit5_Contig145.1 | repressor LexA | *lexA* | + | Opitutaceae sp. TAV5 |
| 2510269383 | Opit5_Contig145.1 | repressor LexA | *lexA* | + | Opitutaceae sp. TAV5 |
| 2517755102 | ObacDRAFT_Scaffold2.1 | recombination protein RecA | *recA* | + | Opitutaceae sp. TAV2 |
| 2517878246 | VerrucomO14_gi399212539.313 | recombination protein RecA | *recA* | - | Verrucomicrobia bacterium SCGC AAA164-O14 (genbank_version) |
| 2517879136 | VerrucomO14_gi399212144.708 | repressor LexA | *lexA* | - | Verrucomicrobia bacterium SCGC AAA164-O14 (genbank_version) |
| 2517881816 | VerrucomE21_gi399211052.191 | recombination protein RecA | *recA* | - | Verrucomicrobia bacterium SCGC AAA168-E21 (genbank_version) |
| 2517882839 | VerrucomE21_gi399210541.702 | repressor LexA | *lexA* | + | Verrucomicrobia bacterium SCGC AAA168-E21 (genbank_version) |
| 2517884759 | VerrucomF10_gi399211052.191 | recombination protein RecA | *recA* | - | Verrucomicrobia bacterium SCGC AAA168-F10 (genbank_version) |
| 2517885782 | VerrucomF10_gi399210541.702 | repressor LexA | *lexA* | + | Verrucomicrobia bacterium SCGC AAA168-F10 (genbank_version) |
| 2518030603 | A37ADRAFT_scaffold1.1 | repressor LexA | *lexA* | - | Verrucomicrobium sp. 3C |
| 2518031718 | A37ADRAFT_scaffold3.3 | recombination protein RecA | *recA* | - | Verrucomicrobium sp. 3C |
| 2519011209 | F454DRAFT_scaffold00008.8 | SOS-response transcriptional repressor, LexA | *lexA* | + | Rubritalea marina DSM 17716 |
| 2519012271 | F454DRAFT_scaffold00024.24 | recombination protein RecA | *recA* | + | Rubritalea marina DSM 17716 |
| 2524330809 | D412DRAFT_2518285663.28 | SOS-response transcriptional repressor, LexA | *lexA* | - | Verrucomicrobia bacterium SCGC AAA027-I19 |
| 2524331246 | D412DRAFT_2518285628.58 | recombination protein RecA | *recA* | - | Verrucomicrobia bacterium SCGC AAA027-I19 |
| 2539612578 | CAHT01000030 | recombination protein RecA | *recA* | - | Methylacidiphilum fumariolicum SolV |
| 2541015094 | G346DRAFT_scf7180000000013_quiver.1 | recombination protein RecA | *recA* | - | Verrucomicrobium sp. LP2A |
| 2541015433 | G346DRAFT_scf7180000000012_quiver.2 | hypothetical protein | *imuA* | + | Verrucomicrobium sp. LP2A |
| 2541015653 | G346DRAFT_scf7180000000012_quiver.2 | repressor LexA | *lexA* | + | Verrucomicrobium sp. LP2A |
| 2582639043 | ME12173DRAFT_MEint_metabat_12173_383000059.100 | SOS-response transcriptional repressor, LexA | *lexA* | + | Composite genome from Lake Mendota Epilimnion pan-assembly MEint.metabat.12173 |
| 2582639636 | ME12173DRAFT_MEint_metabat_12173_604000279.170 | recombination protein RecA | *recA* | + | Composite genome from Lake Mendota Epilimnion pan-assembly MEint.metabat.12173 |
| 2582643281 | ME12612DRAFT_MEint_metabat_12612_36000558.11 | SOS-response transcriptional repressor, LexA | *lexA* | + | Composite genome from Lake Mendota Epilimnion pan-assembly MEint.metabat.12612 |
| 2582644163 | ME12612DRAFT_MEint_metabat_12612_2347001572.126 | recombination protein RecA | *recA* | - | Composite genome from Lake Mendota Epilimnion pan-assembly MEint.metabat.12612 |
| 2582645813 | ME12657DRAFT_MEint_metabat_12657_206000876.33 | recombination protein RecA | *recA* | - | Composite genome from Lake Mendota Epilimnion pan-assembly MEint.metabat.12657 |
| 2582646426 | ME12657DRAFT_MEint_metabat_12657_66000142.74 | repressor LexA | *lexA* | + | Composite genome from Lake Mendota Epilimnion pan-assembly MEint.metabat.12657 |
| 2582697379 | ME2014DRAFT_MEint_metabat_2014_26001367.104 | recombination protein RecA | *recA* | - | Composite genome from Lake Mendota Epilimnion pan-assembly MEint.metabat.2014 |
| 2582697965 | ME2014DRAFT_MEint_metabat_2014_464002082.160 | repressor LexA | *lexA* | - | Composite genome from Lake Mendota Epilimnion pan-assembly MEint.metabat.2014 |
| 2582726124 | ME30509DRAFT_MEint_metabat_30509_2300002348.59 | repressor LexA | *lexA* | + | Composite genome from Lake Mendota Epilimnion pan-assembly MEint.metabat.30509 |
| 2582726632 | ME30509DRAFT_MEint_metabat_30509_2186001660.147 | recombination protein RecA | *recA* | - | Composite genome from Lake Mendota Epilimnion pan-assembly MEint.metabat.30509 |
| 2582752015 | ME3880DRAFT_MEint_metabat_3880_185001469.34 | recombination protein RecA | *recA* | + | Composite genome from Lake Mendota Epilimnion pan-assembly MEint.metabat.3880 |
| 2582752176 | ME3880DRAFT_MEint_metabat_3880_82002817.45 | SOS-response transcriptional repressor, LexA | *lexA* | - | Composite genome from Lake Mendota Epilimnion pan-assembly MEint.metabat.3880 |
| 2582822985 | ME8366DRAFT_MEint_metabat_8366_17000081.32 | recombination protein RecA | *recA* | - | Composite genome from Lake Mendota Epilimnion pan-assembly MEint.metabat.8366 |
| 2582824582 | ME8366DRAFT_MEint_metabat_8366_2293001869.171 | hypothetical protein | *imuA* | + | Composite genome from Lake Mendota Epilimnion pan-assembly MEint.metabat.8366 |
| 2582842543 | TE1301DRAFT_TBepi_metabat_1301_1000753.9 | recombination protein RecA | *recA* | - | Composite genome from Trout Bog Epilimnion pan-assembly TBepi.metabat.1301 |
| 2582844276 | TE1301DRAFT_TBepi_metabat_1301_1000095.97 | repressor LexA | *lexA* | - | Composite genome from Trout Bog Epilimnion pan-assembly TBepi.metabat.1301 |
| 2582880463 | TE4605DRAFT_TBepi_metabat_4605_1005844.152 | recombination protein RecA | *recA* | - | Composite genome from Trout Bog Epilimnion pan-assembly TBepi.metabat.4605 |
| 2582880774 | TE4605DRAFT_TBepi_metabat_4605_1003476.185 | SOS-response transcriptional repressor, LexA | *lexA* | - | Composite genome from Trout Bog Epilimnion pan-assembly TBepi.metabat.4605 |
| 2582941990 | TH2746DRAFT_TBhypo_metabat_2746_10005134.97 | recombination protein RecA | *recA* | - | Composite genome from Trout Bog Hypolimnion pan-assembly TBhypo.metabat.2746 |
| 2582947012 | TH2747DRAFT_TBhypo_metabat_2747_10032833.24 | recombination protein RecA | *recA* | - | Composite genome from Trout Bog Hypolimnion pan-assembly TBhypo.metabat.2747 |
| 2582948466 | TH2747DRAFT_TBhypo_metabat_2747_10002879.165 | SOS-response transcriptional repressor, LexA | *lexA* | - | Composite genome from Trout Bog Hypolimnion pan-assembly TBhypo.metabat.2747 |
| 2582959494 | TH3004DRAFT_TBhypo_metabat_3004_10008814.178 | recombination protein RecA | *recA* | - | Composite genome from Trout Bog Hypolimnion pan-assembly TBhypo.metabat.3004 |
| 2582960083 | TH3004DRAFT_TBhypo_metabat_3004_10005036.223 | repressor LexA | *lexA* | + | Composite genome from Trout Bog Hypolimnion pan-assembly TBhypo.metabat.3004 |
| 2582988720 | TH4093DRAFT_TBhypo_metabat_4093_10001519.100 | SOS-response transcriptional repressor, LexA | *lexA* | + | Composite genome from Trout Bog Hypolimnion pan-assembly TBhypo.metabat.4093 |
| 2582990911 | TH4093DRAFT_TBhypo_metabat_4093_10012276.302 | recombination protein RecA | *recA* | - | Composite genome from Trout Bog Hypolimnion pan-assembly TBhypo.metabat.4093 |
| 2583004830 | TH4590DRAFT_TBhypo_metabat_4590_10019306.101 | hypothetical protein | *imuA* | - | Composite genome from Trout Bog Hypolimnion pan-assembly TBhypo.metabat.4590 |
| 2583004831 | TH4590DRAFT_TBhypo_metabat_4590_10019306.101 | repressor LexA | *lexA* | - | Composite genome from Trout Bog Hypolimnion pan-assembly TBhypo.metabat.4590 |
| 2583006881 | TH4590DRAFT_TBhypo_metabat_4590_10001934.353 | recombination protein RecA | *recA* | - | Composite genome from Trout Bog Hypolimnion pan-assembly TBhypo.metabat.4590 |
| 2583015296 | TH4820DRAFT_TBhypo_metabat_4820_10024412.198 | RecA protein | *recA* | - | Composite genome from Trout Bog Hypolimnion pan-assembly TBhypo.metabat.4820 |
| 2585418795 | EJ93DRAFT_scaffold00003.3 | recombination protein RecA | *recA* | + | Rubritalea squalenifaciens DSM 18772 |
| 2585418854 | EJ93DRAFT_scaffold00003.3 | SOS-response transcriptional repressor, LexA | *lexA* | + | Rubritalea squalenifaciens DSM 18772 |
| 2595246978 | TH02519DRAFT_TH02519_TBL_comb47_HYPODRAFT_10003679.37 | repressor LexA | *lexA* | - | Composite genome from Trout Bog Hypolimnion pan-assembly TBhypo.metabat.2519.v2 |
| 2595259387 | TE01800DRAFT_TE01800_TBL_comb48_EPIDRAFT_1000542.21 | recA DNA recombination protein | *recA* | + | Composite genome from Trout Bog Epilimnion pan-assembly TBepi.metabat.1800.v2 |
| 2595259388 | TE01800DRAFT_TE01800_TBL_comb48_EPIDRAFT_1000542.21 | protein RecA | *recA* | + | Composite genome from Trout Bog Epilimnion pan-assembly TBepi.metabat.1800.v2 |
| 2595259627 | TE01800DRAFT_TE01800_TBL_comb48_EPIDRAFT_1000765.31 | repressor LexA | *lexA* | + | Composite genome from Trout Bog Epilimnion pan-assembly TBepi.metabat.1800.v2 |
| 2609753506 | Ga0069920_122 | repressor LexA | *lexA* | + | Chthoniobacterales bacterium JGI 000193CP-H04 |
| 2609753507 | Ga0069920_122 | recA DNA recombination protein | *recA* | + | Chthoniobacterales bacterium JGI 000193CP-H04 |
| 2609753976 | Ga0069920_154 | recA DNA recombination protein | *recA* | + | Chthoniobacterales bacterium JGI 000193CP-H04 |
| 2612356789 | Ga0056857_1041 | recombination protein RecA | *recA* | + | Verrucomicrobium sp. BvORR106 |
| 2612357500 | Ga0056857_1061 | repressor LexA | *lexA* | - | Verrucomicrobium sp. BvORR106 |
| 2612359469 | Ga0056857_1109 | hypothetical protein | *imuA* | - | Verrucomicrobium sp. BvORR106 |
| 2612380036 | Ga0056855_1008 | recombination protein RecA | *recA* | + | Verrucomicrobium sp. BvORR034 |
| 2612380763 | Ga0056855_1024 | repressor LexA | *lexA* | - | Verrucomicrobium sp. BvORR034 |
| 2612385842 | Ga0056855_1168 | hypothetical protein | *imuA* | - | Verrucomicrobium sp. BvORR034 |
| 2612405553 | Ga0056856_103 | repressor LexA | *lexA* | + | Haloferula sp. BvORR071 |
| 2612409065 | Ga0056856_133 | recombination protein RecA | *recA* | - | Haloferula sp. BvORR071 |
| 2612411457 | Ga0056856_160 | hypothetical protein | *imuA* | + | Haloferula sp. BvORR071 |
| 2617220117 | Ga0073124_1011 | recombination protein RecA | *recA* | + | Dpulchra_bleached_metagenome_bin376 Ga0073124 |
| 2617265577 | Ga0073125_1117 | recombination protein RecA | *recA* | - | Dpulchra_bleached_metagenome_bin377 Ga0073125 |
| 2617266857 | Ga0073125_1201 | repressor LexA | *lexA* | + | Dpulchra_bleached_metagenome_bin377 Ga0073125 |
| 2619624192 | Ga0073400_111 | repressor LexA | *lexA* | - | Verrucomicrobia bacterium SCGC AAA027-I19 (contamination screened) |
| 2619624559 | Ga0073400_119 | recombination protein RecA | *recA* | - | Verrucomicrobia bacterium SCGC AAA027-I19 (contamination screened) |
| 2620029940 | Ga0073653_1171 | repressor LexA | *lexA* | + | Verrucomicrobiaceae bacterium EBPR_Bin_208 |
| 2620030302 | Ga0073653_1192 | recombination protein RecA | *recA* | - | Verrucomicrobiaceae bacterium EBPR_Bin_208 |
| 2620032610 | Ga0073654_1048 | repressor LexA | *lexA* | - | Verrucomicrobiaceae bacterium EBPR_Bin_287 |
| 2620034414 | Ga0073654_1087 | recombination protein RecA | *recA* | + | Verrucomicrobiaceae bacterium EBPR_Bin_287 |
| 2620035247 | Ga0073655_1006 | recombination protein RecA | *recA* | + | Opitutaceae bacterium EBPR_Bin_179 |
| 2620038314 | Ga0073655_1117 | recombination protein RecA | *recA* | + | Opitutaceae bacterium EBPR_Bin_179 |
| 2621053551 | Ga0055369_115 | recombination protein RecA | *recA* | + | Akkermansia muciniphila ATCC BAA-835 |
| 2624341835 | Ga0077533_1143 | recombination protein RecA | *recA* | + | Verrucomicrobia sp. genome_bin_22 Ga0077533 |
| 2630033826 | Ga0070832_101 | recombination protein RecA | *recA* | + | Methylacidiphilum kamchatkense Kam1 |
| 2632215957 | Ga0077870_11 | recombination protein RecA | *recA* | - | Verrucomicrobia bacterium IMCC26134 |
| 2632217020 | Ga0077870_11 | repressor LexA | *lexA* | - | Verrucomicrobia bacterium IMCC26134 |
| 2632639835 | Ga0069468_11 | recombination protein RecA | *recA* | - | Methylacidiphilum fumariolicum SolV |
| 2634850797 | Ga0081615_1018 | recombination protein RecA | *recA* | + | Opitutae-129 (UID2982) |
| 2634851373 | Ga0081615_1035 | recA DNA recombination protein | *recA* | - | Opitutae-129 (UID2982) |
| 2634851374 | Ga0081615_1035 | repressor LexA | *lexA* | - | Opitutae-129 (UID2982) |
| 2634882408 | Ga0081625_101 | recombination protein RecA | *recA* | + | Opititae-40 (UID2982) |
| 641690509 | NC_010571 | recA protein | *recA* | - | Opitutus terrae PB90-1 |
| 641691955 | NC_010571 | SOS-response transcriptional repressor, LexA | *lexA* | - | Opitutus terrae PB90-1 |
| 641693723 | NC_010571 | hypothetical protein | *imuA* | - | Opitutus terrae PB90-1 |
| 641693724 | NC_010571 | SOS-response transcriptional repressor, LexA | *lexA* | - | Opitutus terrae PB90-1 |
| 642329995 | NZ_ABIZ01000001 | SOS-response transcriptional repressor, LexA | *lexA* | - | Verrucomicrobium spinosum DSM 4136, unfinished sequence |
| 642330155 | NZ_ABIZ01000001 | recA domain protein | *imuA* | + | Verrucomicrobium spinosum DSM 4136, unfinished sequence |
| 642335464 | NZ_ABIZ01000001 | recA protein | *recA* | - | Verrucomicrobium spinosum DSM 4136, unfinished sequence |
| 642612715 | NC_010655 | recA protein | *recA* | + | Akkermansia muciniphila ATCC BAA-835 |
| 642665773 | NC_010794 | recA/RadA recombinase | *recA* | - | Methylacidiphilum infernorum V4 |
| 642907294 | NZ_ABVL01000001 | recA protein | *recA* | + | Chthoniobacter flavus Ellin428, unfinished sequence |
| 642912055 | NZ_ABVL01000017 | SOS-response transcriptional repressor, LexA | *lexA* | - | Chthoniobacter flavus Ellin428, unfinished sequence |
| 642913471 | NZ_ABVL01000035 | hypothetical protein | *imuA* | + | Chthoniobacter flavus Ellin428, unfinished sequence |
| 645132786 | NZ_ABOX02000009 | recA protein | *recA* | - | bacterium Ellin514 |
| 645136788 | NZ_ABOX02000063 | SOS-response transcriptional repressor, LexA | *lexA* | - | bacterium Ellin514 |
| 646713453 | NC_014008 | transcriptional repressor, LexA family | *lexA* | + | Coraliomargarita akajimensis DSM 45221 chromosome |
| 646714337 | NC_014008 | recA protein | *recA* | + | Coraliomargarita akajimensis DSM 45221 chromosome |
| 647601401 | NZ_DS990592 | hypothetical protein | *imuA* | - | Verrucomicrobiae bacterium DG1235 scf_1104243000192 genomic scaffold |
| 647602054 | NZ_DS990592 | protein RecA | *recA* | + | Verrucomicrobiae bacterium DG1235 scf_1104243000192 genomic scaffold |
