# Supplementary material for: The Verrucomicrobia LexA-Binding Motif: Insights into the Evolutionary Dynamics of the SOS Response
Source: Front Mol Biosci. 2016 Jul 20;3:33. doi: 10.3389/fmolb.2016.00033 (PMC4951493; doi:10.3389/fmolb.2016.00033)
Supplement: Supplementary file 3 [file Table3.DOCX]

Supplementary Material

The Verrucomicrobia LexA-binding Motif: Insights into the Evolutionary Dynamics of the SOS Response

Ivan Erill^1^, Susana Campoy^2^, Sefa Kılıç^1^ and Jordi Barbé^2*^

*** Correspondence:** Jordi Barbé, jordi.barbe@uab.cat

Supplementary material 3 – Table S3 – List of 55 putative LexA-binding sites identified by MEME on Verrucomicrobia *lexA*, *recA* and *imuA* genes. For each site, the table reports its strand and start position (with respect to -250 bp upstream of the predicted translation start site), the p-value computed by MEME and the site sequence with its flanking regions, as well as the JGI Gene ID and locus tag, gene product description, gene name, strand and species of the gene associated with the site.

| **Gene** | | | | | **Site** | | | | | |
| --- | --- | --- | --- | --- | --- | --- | --- | --- | --- | --- |
| **ID (JGI)** | **Locus Tag (JGI)** | **Product** | **Name** | **Species** | **Str.** | **Start** | **P-value** | **Upstream flank** | **Site** | **Downstream flank** |
| 642913471 | NZ_ABVL01000035 | hypothetical protein | *imuA* | Chthoniobacter flavus Ellin428, unfinished sequence | + | 29 | 3.05E-07 | GAGTGGTATA | TGTTCGTGTGAACA | TATATGCGTG |
| 642907294 | NZ_ABVL01000001 | recA protein | *recA* | Chthoniobacter flavus Ellin428, unfinished sequence | - | 77 | 9.89E-06 | GCAAAACTTG | TGGTCATTTGTACA | GTGACCCCTC |
| 2609753506 | Ga0069920_122 | repressor LexA | *lexA* | Chthoniobacterales bacterium JGI 000193CP-H04 | - | 215 | 4.35E-06 | CGGTGGCGCA | AGTTCAAATGAACA | CACACGTTGC |
| 2609753507 | Ga0069920_122 | recA DNA recombination protein | *recA* | Chthoniobacterales bacterium JGI 000193CP-H04 | - | 209 | 3.08E-08 | ACGATGAATA | TGTTCAAGTGAACA | GTTCGATGCT |
| 2582639043 | ME12173DRAFT_MEint_metabat_12173_383000059.100 | SOS-response transcriptional repressor, LexA | *lexA* | Composite genome from Lake Mendota Epilimnion pan-assembly MEint.metabat.12173 | - | 226 | 4.84E-07 | TGATGAACAG | TGTTCGACAGAACA | AAACAAAATA |
| 2582639636 | ME12173DRAFT_MEint_metabat_12173_604000279.170 | recombination protein RecA | *recA* | Composite genome from Lake Mendota Epilimnion pan-assembly MEint.metabat.12173 | - | 177 | 1.04E-08 | ATAAAAAAGA | TGTTCGATTGAACA | AAAATATGAA |
| 2582643281 | ME12612DRAFT_MEint_metabat_12612_36000558.11 | SOS-response transcriptional repressor, LexA | *lexA* | Composite genome from Lake Mendota Epilimnion pan-assembly MEint.metabat.12612 | - | 223 | 7.56E-07 | CGTTGAACAG | TGTTCTTGAGAACA | AATTCACCGA |
| 2582645813 | ME12657DRAFT_MEint_metabat_12657_206000876.33 | recombination protein RecA | *recA* | Composite genome from Lake Mendota Epilimnion pan-assembly MEint.metabat.12657 | + | 171 | 5.93E-05 | TAGGGTAAGG | TGGATTAGTGAACA | TTTGTTCATT |
| 2582752176 | ME3880DRAFT_MEint_metabat_3880_82002817.45 | SOS-response transcriptional repressor, LexA | *lexA* | Composite genome from Lake Mendota Epilimnion pan-assembly MEint.metabat.3880 | + | 225 | 5.93E-07 | TCGCATGCTT | TGTTCCCTCGAACA | CTGTTCAAAA |
| 2582752015 | ME3880DRAFT_MEint_metabat_3880_185001469.34 | recombination protein RecA | *recA* | Composite genome from Lake Mendota Epilimnion pan-assembly MEint.metabat.3880 | + | 11 | 2.69E-07 | GTCACTTTTT | TGTTCGAGCGAACA | TCTTTTTTCT |
| 2595259627 | TE01800DRAFT_TE01800_TBL_comb48_EPIDRAFT_1000765.31 | repressor LexA | *lexA* | Composite genome from Trout Bog Epilimnion pan-assembly TBepi.metabat.1800.v2 | - | 229 | 8.96E-08 | ATGAACAG | TGTTCTTTTGAACA | CTAATTATTG |
| 2582880774 | TE4605DRAFT_TBepi_metabat_4605_1003476.185 | SOS-response transcriptional repressor, LexA | *lexA* | Composite genome from Trout Bog Epilimnion pan-assembly TBepi.metabat.4605 | + | 224 | 1.27E-06 | TGAGAGGGAA | TGTTCGCCCGAACA | GCCCTTATTC |
| 2582880463 | TE4605DRAFT_TBepi_metabat_4605_1005844.152 | recombination protein RecA | *recA* | Composite genome from Trout Bog Epilimnion pan-assembly TBepi.metabat.4605 | + | 206 | 1.27E-06 | ATGCGCTTTC | TGTTCGCCCGAACA | GCTTTTTATC |
| 2595246978 | TH02519DRAFT_TH02519_TBL_comb47_HYPODRAFT_10003679.37 | repressor LexA | *lexA* | Composite genome from Trout Bog Hypolimnion pan-assembly TBhypo.metabat.2519.v2 | - | 229 | 8.96E-08 | ATGAACAG | TGTTCTTTTGAACA | CTAATTATTG |
| 2582941990 | TH2746DRAFT_TBhypo_metabat_2746_10005134.97 | recombination protein RecA | *recA* | Composite genome from Trout Bog Hypolimnion pan-assembly TBhypo.metabat.2746 | - | 178 | 1.27E-06 | AGAAAAAAGA | TGTTCGCCCGAACA | AAAAAGTGAG |
| 2582947012 | TH2747DRAFT_TBhypo_metabat_2747_10032833.24 | recombination protein RecA | *recA* | Composite genome from Trout Bog Hypolimnion pan-assembly TBhypo.metabat.2747 | + | 167 | 3.10E-05 | CCGGCAATGT | TGATCCGGTGAACA | ACGCGCGTTT |
| 2583004831 | TH4590DRAFT_TBhypo_metabat_4590_10019306.101 | repressor LexA | *lexA* | Composite genome from Trout Bog Hypolimnion pan-assembly TBhypo.metabat.4590 | + | 233 | 3.38E-09 | CGGGATCGCG | TGTTCAATTGAACA | CATC |
| 646713453 | NC_014008 | transcriptional repressor, LexA family | *lexA* | Coraliomargarita akajimensis DSM 45221 chromosome | - | 227 | 2.32E-06 | AGTGTTCACT | TGTTTAGGTGAACA | ACTGAACAGT |
| 2617220117 | Ga0073124_1011 | recombination protein RecA | *recA* | Dpulchra_bleached_metagenome_bin376 Ga0073124 | + | 205 | 2.32E-06 | TCTGTAAAGA | TGCTCATCTGAACA | GTTTTTAGAC |
| 2617266857 | Ga0073125_1201 | repressor LexA | *lexA* | Dpulchra_bleached_metagenome_bin377 Ga0073125 | + | 229 | 8.96E-08 | ATAGATACTA | TGTTCTATAGAACA | CTACTCTT |
| 2612411457 | Ga0056856_160 | hypothetical protein | *imuA* | Haloferula sp. BvORR071 | - | 207 | 3.38E-09 | AATTTTAAAA | TGTTCAATTGAACA | CTTTAATTTC |
| 2612405553 | Ga0056856_103 | repressor LexA | *lexA* | Haloferula sp. BvORR071 | + | 223 | 1.01E-06 | GACCATGGTT | TGTTCCTAAGAACA | CTGTTCACTA |
| 2612409065 | Ga0056856_133 | recombination protein RecA | *recA* | Haloferula sp. BvORR071 | + | 174 | 2.69E-07 | GTCTCATTTT | TGTTCGAGCGAACA | GCTTTTTCGT |
| 2620035247 | Ga0073655_1006 | recombination protein RecA | *recA* | Opitutaceae bacterium EBPR_Bin_179 | - | 219 | 1.27E-06 | GTGGTTAATT | TGTTCCGGTGAACA | CTTAGGGTGG |
| 2510269383 | Opit5_Contig145.1 | repressor LexA | *lexA* | Opitutaceae sp. TAV5 | - | 201 | 3.08E-08 | ATACTCCTAG | TGTTCAAGTGAACA | CATATAGCCA |
| 2634851374 | Ga0081615_1035 | repressor LexA | *lexA* | Opitutae-129 (UID2982) | - | 235 | 2.32E-06 | AG | GGTTCAATTGAACA | CGGTTCAAAT |
| 641693724 | NC_010571 | SOS-response transcriptional repressor, LexA | *lexA* | Opitutus terrae PB90-1 | + | 229 | 1.25E-07 | CTTGAACATG | TGTTCAAGAGAACA | CATCCGCC |
| 2519012271 | F454DRAFT_scaffold00024.24 | recombination protein RecA | *recA* | Rubritalea marina DSM 17716 | + | 133 | 8.96E-08 | AATAATAATT | TGTTCTTTTGAACA | CAATTTATAG |
| 2585418854 | EJ93DRAFT_scaffold00003.3 | SOS-response transcriptional repressor, LexA | *lexA* | Rubritalea squalenifaciens DSM 18772 | - | 222 | 5.93E-07 | AATTGAACAC | TGTTCTTAAGAACA | CAATGCCTTG |
| 2585418795 | EJ93DRAFT_scaffold00003.3 | recombination protein RecA | *recA* | Rubritalea squalenifaciens DSM 18772 | + | 130 | 1.75E-06 | TTATCAAAAA | TGTTCCTCCGAACA | CCTTTTTGGT |
| 2632217020 | Ga0077870_11 | repressor LexA | *lexA* | Verrucomicrobia bacterium IMCC26134 | - | 215 | 3.08E-08 | TTGTGAACCG | TGTTCAAGTGAACA | CATGACCGGC |
| 2524330809 | D412DRAFT_2518285663.28 | SOS-response transcriptional repressor, LexA | *lexA* | Verrucomicrobia bacterium SCGC AAA027-I19 | - | 226 | 4.84E-07 | TGATGAACAG | TGTTCGACAGAACA | AAACAAAATA |
| 2524331246 | D412DRAFT_2518285628.58 | recombination protein RecA | *recA* | Verrucomicrobia bacterium SCGC AAA027-I19 | - | 177 | 1.04E-08 | ATAAAAAAGA | TGTTCGATTGAACA | AAAATATGAA |
| 2619624192 | Ga0073400_111 | repressor LexA | *lexA* | Verrucomicrobia bacterium SCGC AAA027-I19 (contamination screened) | - | 226 | 4.84E-07 | TGATGAACAG | TGTTCGACAGAACA | AAACAAAATA |
| 2619624559 | Ga0073400_119 | recombination protein RecA | *recA* | Verrucomicrobia bacterium SCGC AAA027-I19 (contamination screened) | - | 177 | 1.04E-08 | ATAAAAAAGA | TGTTCGATTGAACA | AAAATATGAA |
| 2517879136 | VerrucomO14_gi399212144.708 | repressor LexA | *lexA* | Verrucomicrobia bacterium SCGC AAA164-O14 (genbank_version) | - | 227 | 1.63E-06 | GTAAGACTAT | TGTTTCAATGAACA | CTATTTGTCC |
| 2517878246 | VerrucomO14_gi399212539.313 | recombination protein RecA | *recA* | Verrucomicrobia bacterium SCGC AAA164-O14 (genbank_version) | + | 189 | 6.68E-06 | TTGAAGTTTT | TGCTTTTTTGAACA | CTGTTCATAT |
| 2517882839 | VerrucomE21_gi399210541.702 | repressor LexA | *lexA* | Verrucomicrobia bacterium SCGC AAA168-E21 (genbank_version) | - | 227 | 1.63E-06 | GTAAGACTAT | TGTTTCAATGAACA | CTATTTGTCC |
| 2517881816 | VerrucomE21_gi399211052.191 | recombination protein RecA | *recA* | Verrucomicrobia bacterium SCGC AAA168-E21 (genbank_version) | + | 189 | 6.68E-06 | TTGAAGTTTT | TGCTTTTTTGAACA | CTGTTCATAT |
| 2517885782 | VerrucomF10_gi399210541.702 | repressor LexA | *lexA* | Verrucomicrobia bacterium SCGC AAA168-F10 (genbank_version) | - | 227 | 1.63E-06 | GTAAGACTAT | TGTTTCAATGAACA | CTATTTGTCC |
| 2517884759 | VerrucomF10_gi399211052.191 | recombination protein RecA | *recA* | Verrucomicrobia bacterium SCGC AAA168-F10 (genbank_version) | + | 189 | 6.68E-06 | TTGAAGTTTT | TGCTTTTTTGAACA | CTGTTCATAT |
| 2236434052 | A168E21DRAFT_NODE-unique_18_len_31805.18 | SOS regulatory protein LexA | *lexA* | Verrucomicrobia SCGC AAA168-E21 | - | 227 | 1.63E-06 | GTAAGACTAT | TGTTTCAATGAACA | CTATTTGTCC |
| 2236435003 | A168E21DRAFT_NODE-unique_83_len_6729.83 | protein RecA | *recA* | Verrucomicrobia SCGC AAA168-E21 | + | 189 | 6.68E-06 | TTGAAGTTTT | TGCTTTTTTGAACA | CTGTTCATAT |
| 2236454024 | A168F10DRAFT_NODE-unique_23_len_53784.23 | SOS regulatory protein LexA | *lexA* | Verrucomicrobia SCGC AAA168-F10 | - | 227 | 1.63E-06 | GTAAGACTAT | TGTTTCAATGAACA | CTATTTGTCC |
| 2236453899 | A168F10DRAFT_NODE-unique_20_len_54947.20 | protein RecA | *recA* | Verrucomicrobia SCGC AAA168-F10 | + | 189 | 6.68E-06 | TTGAAGTTTT | TGCTTTTTTGAACA | CTGTTCATAT |
| 2620030302 | Ga0073653_1192 | recombination protein RecA | *recA* | Verrucomicrobiaceae bacterium EBPR_Bin_208 | + | 198 | 3.10E-05 | ACTTTTTTAC | TGGACTTGTGAACA | CTTTCCAAGT |
| 2620032610 | Ga0073654_1048 | repressor LexA | *lexA* | Verrucomicrobiaceae bacterium EBPR_Bin_287 | - | 228 | 9.41E-07 | TATGAACAC | TGTTCCACAGAACA | TCAGATAATT |
| 2620034414 | Ga0073654_1087 | recombination protein RecA | *recA* | Verrucomicrobiaceae bacterium EBPR_Bin_287 | + | 201 | 2.33E-05 | ACTTTTTTAC | TGGACCTTTGAACA | GTTAACTGTA |
| 2612380763 | Ga0056855_1024 | repressor LexA | *lexA* | Verrucomicrobium sp. BvORR034 | + | 221 | 5.40E-06 | AACTCTTGAC | TGCACAATCGAACA | GTGTTCACAT |
| 2612380036 | Ga0056855_1008 | recombination protein RecA | *recA* | Verrucomicrobium sp. BvORR034 | - | 194 | 1.04E-08 | AGAGCGATAA | TGTTCGATTGAACA | GGAGTTGCAA |
| 2612359469 | Ga0056857_1109 | hypothetical protein | *imuA* | Verrucomicrobium sp. BvORR106 | - | 227 | 1.04E-07 | AAACAAATAG | TGTTCGAGTGAACA | CTATTTGTTT |
| 2612357500 | Ga0056857_1061 | repressor LexA | *lexA* | Verrucomicrobium sp. BvORR106 | + | 221 | 5.40E-06 | AACTCTTGAC | TGCACAATCGAACA | GTGTTCACAT |
| 2612356789 | Ga0056857_1041 | recombination protein RecA | *recA* | Verrucomicrobium sp. BvORR106 | - | 194 | 1.04E-08 | AGAGCGATAA | TGTTCGATTGAACA | GGAGTTGCAA |
| 642329995 | NZ_ABIZ01000001 | SOS-response transcriptional repressor, LexA | *lexA* | Verrucomicrobium spinosum DSM 4136, unfinished sequence | + | 221 | 1.44E-05 | AACTCTTGAC | TGCACAACCGAACA | GTGTTCACAT |
| 642335464 | NZ_ABIZ01000001 | recA protein | *recA* | Verrucomicrobium spinosum DSM 4136, unfinished sequence | - | 194 | 1.04E-08 | AGAGCGATAA | TGTTCGATTGAACA | GGAGTTGCAA |
