# Supplementary material for: The Verrucomicrobia LexA-Binding Motif: Insights into the Evolutionary Dynamics of the SOS Response
Source: Front Mol Biosci. 2016 Jul 20;3:33. doi: 10.3389/fmolb.2016.00033 (PMC4951493; doi:10.3389/fmolb.2016.00033)
Supplement: Supplementary file 4 [file Table4.DOCX]

Supplementary Material

The Verrucomicrobia LexA-binding Motif: Insights into the Evolutionary Dynamics of the SOS Response

Ivan Erill^1^, Susana Campoy^2^, Sefa Kılıç^1^ and Jordi Barbé^2*^

*** Correspondence:** Jordi Barbé, jordi.barbe@uab.cat

Supplementary material 7 – Table S4 – List of putative LexA-binding sites in analyzed Verrucomicrobia species. The table reports the identified site sequence, the genome assembly contig in which it is located, the strand, start and end positions of the site relative the contig start position, the gene name of the gene it putatively regulates, together with its locus tag, strand and product information, and the distance of the site relative to the gene’s predicted translational start.

| **Coraliomargarita akajimensis DSM 45221** | | | | | | | | | | | |
| --- | --- | --- | --- | --- | --- | --- | --- | --- | --- | --- | --- |
| **score** | **site** | **chromid** | **start** | **end** | **strand** | **distance** | **gene_strand** | **gene_start** | **gene_end** | **gene_locus_tag** | **gene_product** |
| 23.0001 | AAGTGTTCGTTTGAACACAT | NZ_ABVL01000002.1 | 516112 | 516132 | -1 | 301 | 1 | 516433 | 517141 | CFE428DRAFT_RS05050 | hypothetical protein |
| 22.9742 | ATGTGTTCAAACGAACACTT | NZ_ABVL01000002.1 | 516112 | 516132 | 1 | 301 | 1 | 516433 | 517141 | CFE428DRAFT_RS05050 | hypothetical protein |
| 20.5261 | ATGTGTTCAAGAGAACATAT | NZ_ABVL01000002.1 | 347936 | 347956 | -1 | 0 | 1 | 347956 | 349036 | CFE428DRAFT_RS04375 | radical SAM protein |
| 20.49 | ATATGTTCTCTTGAACACAT | NZ_ABVL01000002.1 | 347936 | 347956 | 1 | 0 | 1 | 347956 | 349036 | CFE428DRAFT_RS04375 | radical SAM protein |
| 18.6245 | ATATGTTCGTGTGAACATAT | NZ_ABVL01000035.1 | 31865 | 31885 | 1 | 210 | -1 | 31442 | 31655 | CFE428DRAFT_RS29915 | KTSC domain-containing protein |
| 18.6245 | ATATGTTCGTGTGAACATAT | NZ_ABVL01000035.1 | 31865 | 31885 | 1 | 4 | 1 | 31889 | 32558 | CFE428DRAFT_RS29920 | hypothetical protein |
| 18.5986 | ATATGTTCACACGAACATAT | NZ_ABVL01000035.1 | 31865 | 31885 | -1 | 210 | -1 | 31442 | 31655 | CFE428DRAFT_RS29915 | KTSC domain-containing protein |
| 18.5986 | ATATGTTCACACGAACATAT | NZ_ABVL01000035.1 | 31865 | 31885 | -1 | 4 | 1 | 31889 | 32558 | CFE428DRAFT_RS29920 | hypothetical protein |
| 18.3668 | AAGTGATCATATGAACACTA | NZ_ABVL01000013.1 | 209519 | 209539 | -1 | 215 | 1 | 209754 | 209994 | CFE428DRAFT_RS20475 | hypothetical protein |
| 18.35 | TAGTGTTCATATGATCACTT | NZ_ABVL01000013.1 | 209519 | 209539 | 1 | 215 | 1 | 209754 | 209994 | CFE428DRAFT_RS20475 | hypothetical protein |
| 17.3236 | GCATGTTCTTTAGAACACTA | NZ_ABVL01000042.1 | 28434 | 28454 | -1 | 91 | -1 | 27896 | 28343 | CFE428DRAFT_RS31330 | glycosylase |
| 17.3236 | GCATGTTCTTTAGAACACTA | NZ_ABVL01000042.1 | 28434 | 28454 | -1 | 383 | 1 | 28837 | 29461 | CFE428DRAFT_RS31335 | hypothetical protein |
| 17.2185 | TAGTGTTCTAAAGAACATGC | NZ_ABVL01000042.1 | 28434 | 28454 | 1 | 91 | -1 | 27896 | 28343 | CFE428DRAFT_RS31330 | glycosylase |
| 17.2185 | TAGTGTTCTAAAGAACATGC | NZ_ABVL01000042.1 | 28434 | 28454 | 1 | 383 | 1 | 28837 | 29461 | CFE428DRAFT_RS31335 | hypothetical protein |
| 16.4034 | CAGTGTTCTGGCGAACATGA | NZ_ABVL01000017.1 | 62340 | 62360 | -1 | -4 | -1 | 61732 | 62344 | CFE428DRAFT_RS23045 | repressor LexA |
| 16.4034 | CAGTGTTCTGGCGAACATGA | NZ_ABVL01000017.1 | 62340 | 62360 | -1 | 97 | 1 | 62457 | 63213 | CFE428DRAFT_RS23050 | nucleotidyltransferase |
| 16.3053 | TCATGTTCGCCAGAACACTG | NZ_ABVL01000017.1 | 62340 | 62360 | 1 | -4 | -1 | 61732 | 62344 | CFE428DRAFT_RS23045 | repressor LexA |
| 16.3053 | TCATGTTCGCCAGAACACTG | NZ_ABVL01000017.1 | 62340 | 62360 | 1 | 97 | 1 | 62457 | 63213 | CFE428DRAFT_RS23050 | nucleotidyltransferase |
| 15.6561 | ATGTGTTCATGAAAGCACTA | NZ_ABVL01000042.1 | 3721 | 3741 | 1 | 108 | -1 | 3280 | 3613 | CFE428DRAFT_RS31170 | hypothetical protein |
| 15.393 | TAGTGCTTTCATGAACACAT | NZ_ABVL01000042.1 | 3721 | 3741 | -1 | 108 | -1 | 3280 | 3613 | CFE428DRAFT_RS31170 | hypothetical protein |
| 15.1759 | CACTGTACAAATGACCACAA | NZ_ABVL01000001.1 | 24436 | 24456 | 1 | 78 | -1 | 22660 | 24358 | CFE428DRAFT_RS00135 | hypothetical protein |
| 14.9988 | TTGTGGTCATTTGTACAGTG | NZ_ABVL01000001.1 | 24436 | 24456 | -1 | 78 | -1 | 22660 | 24358 | CFE428DRAFT_RS00135 | hypothetical protein |
| 14.7846 | ATGTGTTCATAAGCACACAA | NZ_ABVL01000002.1 | 330133 | 330153 | 1 | 67 | -1 | 329058 | 330066 | CFE428DRAFT_RS04285 | alpha/beta hydrolase fold protein |
| 14.7548 | GTTTGTTCAGCTAAACAGAA | NZ_ABVL01000012.1 | 207878 | 207898 | -1 | 544 | -1 | 206812 | 207334 | CFE428DRAFT_RS19520 | shikimate kinase |
| 14.7548 | GTTTGTTCAGCTAAACAGAA | NZ_ABVL01000012.1 | 207878 | 207898 | -1 | 42 | 1 | 207940 | 208432 | CFE428DRAFT_RS19525 | 5-(carboxyamino)imidazole ribonucleotide mutase |
| 14.7383 | TTGTGTGCTTATGAACACAT | NZ_ABVL01000002.1 | 330133 | 330153 | -1 | 67 | -1 | 329058 | 330066 | CFE428DRAFT_RS04285 | alpha/beta hydrolase fold protein |
| 14.5521 | TTCTGTTTAGCTGAACAAAC | NZ_ABVL01000012.1 | 207878 | 207898 | 1 | 544 | -1 | 206812 | 207334 | CFE428DRAFT_RS19520 | shikimate kinase |
| 14.5521 | TTCTGTTTAGCTGAACAAAC | NZ_ABVL01000012.1 | 207878 | 207898 | 1 | 42 | 1 | 207940 | 208432 | CFE428DRAFT_RS19525 | 5-(carboxyamino)imidazole ribonucleotide mutase |
| 14.0473 | TTTTGTTTTTGGGAACAATC | NZ_ABVL01000010.1 | 207989 | 208009 | -1 | 59 | -1 | 207168 | 207930 | CFE428DRAFT_RS17575 | hypothetical protein |
| 13.998 | GATTGTTCCCAAAAACAAAA | NZ_ABVL01000010.1 | 207989 | 208009 | 1 | 59 | -1 | 207168 | 207930 | CFE428DRAFT_RS17575 | hypothetical protein |
| 13.9763 | ACTTGTTCTAAGGAACAGCC | NZ_ABVL01000001.1 | 666553 | 666573 | 1 | 190 | -1 | 665751 | 666363 | CFE428DRAFT_RS02810 | hypothetical protein |
| 13.9763 | ACTTGTTCTAAGGAACAGCC | NZ_ABVL01000001.1 | 666553 | 666573 | 1 | 47 | 1 | 666620 | 667385 | CFE428DRAFT_RS02815 | hypothetical protein |
| 13.9737 | GGCTGTTCCTTAGAACAAGT | NZ_ABVL01000001.1 | 666553 | 666573 | -1 | 190 | -1 | 665751 | 666363 | CFE428DRAFT_RS02810 | hypothetical protein |
| 13.9737 | GGCTGTTCCTTAGAACAAGT | NZ_ABVL01000001.1 | 666553 | 666573 | -1 | 47 | 1 | 666620 | 667385 | CFE428DRAFT_RS02815 | hypothetical protein |
| 13.9519 | TTGTGTTCACTGAAACAGGC | NZ_ABVL01000005.1 | 130106 | 130126 | 1 | 115 | -1 | 129646 | 129991 | CFE428DRAFT_RS10325 | hypothetical protein |
| 13.9519 | TTGTGTTCACTGAAACAGGC | NZ_ABVL01000005.1 | 130106 | 130126 | 1 | 102 | 1 | 130228 | 131341 | CFE428DRAFT_RS10330 | PEP-CTERM domain protein |
| 13.7806 | TTGTGTTAGTTTAAACAAAT | NZ_ABVL01000012.1 | 77540 | 77560 | 1 | 154 | -1 | 76897 | 77386 | CFE428DRAFT_RS18875 | pantetheine-phosphate adenylyltransferase |
| 13.7806 | TTGTGTTAGTTTAAACAAAT | NZ_ABVL01000012.1 | 77540 | 77560 | 1 | 163 | 1 | 77723 | 79784 | CFE428DRAFT_RS18880 | hypothetical protein |
| 13.6167 | GCCTGTTTCAGTGAACACAA | NZ_ABVL01000005.1 | 130106 | 130126 | -1 | 115 | -1 | 129646 | 129991 | CFE428DRAFT_RS10325 | hypothetical protein |
| 13.6167 | GCCTGTTTCAGTGAACACAA | NZ_ABVL01000005.1 | 130106 | 130126 | -1 | 102 | 1 | 130228 | 131341 | CFE428DRAFT_RS10330 | PEP-CTERM domain protein |
| **Chthoniobacter flavus Ellin428** | | | | | | | | | | | |
| **score** | **site** | **chromid** | **start** | **end** | **strand** | **distance** | **gene_strand** | **gene_start** | **gene_end** | **gene_locus_tag** | **gene_product** |
| 23.0001 | AAGTGTTCGTTTGAACACAT | NZ_ABVL01000002.1 | 516112 | 516132 | -1 | 301 | 1 | 516433 | 517141 | CFE428DRAFT_RS05050 | hypothetical protein |
| 22.9742 | ATGTGTTCAAACGAACACTT | NZ_ABVL01000002.1 | 516112 | 516132 | 1 | 301 | 1 | 516433 | 517141 | CFE428DRAFT_RS05050 | hypothetical protein |
| 20.5261 | ATGTGTTCAAGAGAACATAT | NZ_ABVL01000002.1 | 347936 | 347956 | -1 | 0 | 1 | 347956 | 349036 | CFE428DRAFT_RS04375 | radical SAM protein |
| 20.49 | ATATGTTCTCTTGAACACAT | NZ_ABVL01000002.1 | 347936 | 347956 | 1 | 0 | 1 | 347956 | 349036 | CFE428DRAFT_RS04375 | radical SAM protein |
| 18.6245 | ATATGTTCGTGTGAACATAT | NZ_ABVL01000035.1 | 31865 | 31885 | 1 | 210 | -1 | 31442 | 31655 | CFE428DRAFT_RS29915 | KTSC domain-containing protein |
| 18.6245 | ATATGTTCGTGTGAACATAT | NZ_ABVL01000035.1 | 31865 | 31885 | 1 | 4 | 1 | 31889 | 32558 | CFE428DRAFT_RS29920 | hypothetical protein |
| 18.5986 | ATATGTTCACACGAACATAT | NZ_ABVL01000035.1 | 31865 | 31885 | -1 | 210 | -1 | 31442 | 31655 | CFE428DRAFT_RS29915 | KTSC domain-containing protein |
| 18.5986 | ATATGTTCACACGAACATAT | NZ_ABVL01000035.1 | 31865 | 31885 | -1 | 4 | 1 | 31889 | 32558 | CFE428DRAFT_RS29920 | hypothetical protein |
| 18.3668 | AAGTGATCATATGAACACTA | NZ_ABVL01000013.1 | 209519 | 209539 | -1 | 215 | 1 | 209754 | 209994 | CFE428DRAFT_RS20475 | hypothetical protein |
| 18.35 | TAGTGTTCATATGATCACTT | NZ_ABVL01000013.1 | 209519 | 209539 | 1 | 215 | 1 | 209754 | 209994 | CFE428DRAFT_RS20475 | hypothetical protein |
| 17.3236 | GCATGTTCTTTAGAACACTA | NZ_ABVL01000042.1 | 28434 | 28454 | -1 | 91 | -1 | 27896 | 28343 | CFE428DRAFT_RS31330 | glycosylase |
| 17.3236 | GCATGTTCTTTAGAACACTA | NZ_ABVL01000042.1 | 28434 | 28454 | -1 | 383 | 1 | 28837 | 29461 | CFE428DRAFT_RS31335 | hypothetical protein |
| 17.2185 | TAGTGTTCTAAAGAACATGC | NZ_ABVL01000042.1 | 28434 | 28454 | 1 | 91 | -1 | 27896 | 28343 | CFE428DRAFT_RS31330 | glycosylase |
| 17.2185 | TAGTGTTCTAAAGAACATGC | NZ_ABVL01000042.1 | 28434 | 28454 | 1 | 383 | 1 | 28837 | 29461 | CFE428DRAFT_RS31335 | hypothetical protein |
| 16.4034 | CAGTGTTCTGGCGAACATGA | NZ_ABVL01000017.1 | 62340 | 62360 | -1 | -4 | -1 | 61732 | 62344 | CFE428DRAFT_RS23045 | repressor LexA |
| 16.4034 | CAGTGTTCTGGCGAACATGA | NZ_ABVL01000017.1 | 62340 | 62360 | -1 | 97 | 1 | 62457 | 63213 | CFE428DRAFT_RS23050 | nucleotidyltransferase |
| 16.3053 | TCATGTTCGCCAGAACACTG | NZ_ABVL01000017.1 | 62340 | 62360 | 1 | -4 | -1 | 61732 | 62344 | CFE428DRAFT_RS23045 | repressor LexA |
| 16.3053 | TCATGTTCGCCAGAACACTG | NZ_ABVL01000017.1 | 62340 | 62360 | 1 | 97 | 1 | 62457 | 63213 | CFE428DRAFT_RS23050 | nucleotidyltransferase |
| 15.6561 | ATGTGTTCATGAAAGCACTA | NZ_ABVL01000042.1 | 3721 | 3741 | 1 | 108 | -1 | 3280 | 3613 | CFE428DRAFT_RS31170 | hypothetical protein |
| 15.393 | TAGTGCTTTCATGAACACAT | NZ_ABVL01000042.1 | 3721 | 3741 | -1 | 108 | -1 | 3280 | 3613 | CFE428DRAFT_RS31170 | hypothetical protein |
| 15.1759 | CACTGTACAAATGACCACAA | NZ_ABVL01000001.1 | 24436 | 24456 | 1 | 78 | -1 | 22660 | 24358 | CFE428DRAFT_RS00135 | hypothetical protein |
| 14.9988 | TTGTGGTCATTTGTACAGTG | NZ_ABVL01000001.1 | 24436 | 24456 | -1 | 78 | -1 | 22660 | 24358 | CFE428DRAFT_RS00135 | hypothetical protein |
| 14.7846 | ATGTGTTCATAAGCACACAA | NZ_ABVL01000002.1 | 330133 | 330153 | 1 | 67 | -1 | 329058 | 330066 | CFE428DRAFT_RS04285 | alpha/beta hydrolase fold protein |
| 14.7548 | GTTTGTTCAGCTAAACAGAA | NZ_ABVL01000012.1 | 207878 | 207898 | -1 | 544 | -1 | 206812 | 207334 | CFE428DRAFT_RS19520 | shikimate kinase |
| 14.7548 | GTTTGTTCAGCTAAACAGAA | NZ_ABVL01000012.1 | 207878 | 207898 | -1 | 42 | 1 | 207940 | 208432 | CFE428DRAFT_RS19525 | 5-(carboxyamino)imidazole ribonucleotide mutase |
| 14.7383 | TTGTGTGCTTATGAACACAT | NZ_ABVL01000002.1 | 330133 | 330153 | -1 | 67 | -1 | 329058 | 330066 | CFE428DRAFT_RS04285 | alpha/beta hydrolase fold protein |
| 14.5521 | TTCTGTTTAGCTGAACAAAC | NZ_ABVL01000012.1 | 207878 | 207898 | 1 | 544 | -1 | 206812 | 207334 | CFE428DRAFT_RS19520 | shikimate kinase |
| 14.5521 | TTCTGTTTAGCTGAACAAAC | NZ_ABVL01000012.1 | 207878 | 207898 | 1 | 42 | 1 | 207940 | 208432 | CFE428DRAFT_RS19525 | 5-(carboxyamino)imidazole ribonucleotide mutase |
| 14.0473 | TTTTGTTTTTGGGAACAATC | NZ_ABVL01000010.1 | 207989 | 208009 | -1 | 59 | -1 | 207168 | 207930 | CFE428DRAFT_RS17575 | hypothetical protein |
| 13.998 | GATTGTTCCCAAAAACAAAA | NZ_ABVL01000010.1 | 207989 | 208009 | 1 | 59 | -1 | 207168 | 207930 | CFE428DRAFT_RS17575 | hypothetical protein |
| 13.9763 | ACTTGTTCTAAGGAACAGCC | NZ_ABVL01000001.1 | 666553 | 666573 | 1 | 190 | -1 | 665751 | 666363 | CFE428DRAFT_RS02810 | hypothetical protein |
| 13.9763 | ACTTGTTCTAAGGAACAGCC | NZ_ABVL01000001.1 | 666553 | 666573 | 1 | 47 | 1 | 666620 | 667385 | CFE428DRAFT_RS02815 | hypothetical protein |
| 13.9737 | GGCTGTTCCTTAGAACAAGT | NZ_ABVL01000001.1 | 666553 | 666573 | -1 | 190 | -1 | 665751 | 666363 | CFE428DRAFT_RS02810 | hypothetical protein |
| 13.9737 | GGCTGTTCCTTAGAACAAGT | NZ_ABVL01000001.1 | 666553 | 666573 | -1 | 47 | 1 | 666620 | 667385 | CFE428DRAFT_RS02815 | hypothetical protein |
| 13.9519 | TTGTGTTCACTGAAACAGGC | NZ_ABVL01000005.1 | 130106 | 130126 | 1 | 115 | -1 | 129646 | 129991 | CFE428DRAFT_RS10325 | hypothetical protein |
| 13.9519 | TTGTGTTCACTGAAACAGGC | NZ_ABVL01000005.1 | 130106 | 130126 | 1 | 102 | 1 | 130228 | 131341 | CFE428DRAFT_RS10330 | PEP-CTERM domain protein |
| 13.7806 | TTGTGTTAGTTTAAACAAAT | NZ_ABVL01000012.1 | 77540 | 77560 | 1 | 154 | -1 | 76897 | 77386 | CFE428DRAFT_RS18875 | pantetheine-phosphate adenylyltransferase |
| 13.7806 | TTGTGTTAGTTTAAACAAAT | NZ_ABVL01000012.1 | 77540 | 77560 | 1 | 163 | 1 | 77723 | 79784 | CFE428DRAFT_RS18880 | hypothetical protein |
| 13.6167 | GCCTGTTTCAGTGAACACAA | NZ_ABVL01000005.1 | 130106 | 130126 | -1 | 115 | -1 | 129646 | 129991 | CFE428DRAFT_RS10325 | hypothetical protein |
| 13.6167 | GCCTGTTTCAGTGAACACAA | NZ_ABVL01000005.1 | 130106 | 130126 | -1 | 102 | 1 | 130228 | 131341 | CFE428DRAFT_RS10330 | PEP-CTERM domain protein |
| **Haloferula sp. BvORR071** | | | | | | | | | | | |
| **score** | **site** | **chromid** | **start** | **end** | **strand** | **distance** | **gene_strand** | **gene_start** | **gene_end** | **gene_locus_tag** | **gene_product** |
| 24.5411 | AAGTGTTCAATTGAACATTT | NZ_BATP01000060.1 | 69247 | 69267 | 1 | 27 | 1 | 69294 | 69921 | BV071_RS30680 | hypothetical protein |
| 24.5072 | AAATGTTCAATTGAACACTT | NZ_BATP01000060.1 | 69247 | 69267 | -1 | 27 | 1 | 69294 | 69921 | BV071_RS30680 | hypothetical protein |
| 23.4386 | TAGTGTTCACTTGAACATTT | NZ_BATP01000035.1 | 32274 | 32294 | -1 | 27 | -1 | 29109 | 32247 | BV071_RS19545 | hypothetical protein |
| 23.4386 | TAGTGTTCACTTGAACATTT | NZ_BATP01000035.1 | 32274 | 32294 | -1 | 61 | 1 | 32355 | 32724 | BV071_RS19550 | hypothetical protein |
| 23.4098 | AAATGTTCAAGTGAACACTA | NZ_BATP01000035.1 | 32274 | 32294 | 1 | 27 | -1 | 29109 | 32247 | BV071_RS19545 | hypothetical protein |
| 23.4098 | AAATGTTCAAGTGAACACTA | NZ_BATP01000035.1 | 32274 | 32294 | 1 | 61 | 1 | 32355 | 32724 | BV071_RS19550 | hypothetical protein |
| 22.9481 | TAGTGTTCATGTGAACAATT | NZ_BATP01000039.1 | 112218 | 112238 | 1 | 60 | -1 | 110859 | 112158 | BV071_RS21175 | hypothetical protein |
| 22.9481 | TAGTGTTCATGTGAACAATT | NZ_BATP01000039.1 | 112218 | 112238 | 1 | 29 | 1 | 112267 | 113332 | BV071_RS21180 | radical SAM protein |
| 22.8914 | AATTGTTCACATGAACACTA | NZ_BATP01000039.1 | 112218 | 112238 | -1 | 60 | -1 | 110859 | 112158 | BV071_RS21175 | hypothetical protein |
| 22.8914 | AATTGTTCACATGAACACTA | NZ_BATP01000039.1 | 112218 | 112238 | -1 | 29 | 1 | 112267 | 113332 | BV071_RS21180 | radical SAM protein |
| 22.1345 | AAGTGTTCTTTTGAACATAA | NZ_BATP01000003.1 | 313824 | 313844 | 1 | 193 | -1 | 313232 | 313631 | BV071_RS02190 | hypothetical protein |
| 22.1345 | AAGTGTTCTTTTGAACATAA | NZ_BATP01000003.1 | 313824 | 313844 | 1 | -10 | 1 | 313834 | 314089 | BV071_RS02195 | hypothetical protein |
| 22.0429 | TTATGTTCAAAAGAACACTT | NZ_BATP01000003.1 | 313824 | 313844 | -1 | 193 | -1 | 313232 | 313631 | BV071_RS02190 | hypothetical protein |
| 22.0429 | TTATGTTCAAAAGAACACTT | NZ_BATP01000003.1 | 313824 | 313844 | -1 | -10 | 1 | 313834 | 314089 | BV071_RS02195 | hypothetical protein |
| 21.3582 | ATTTGTTCTTTTGAACAGTT | NZ_BATP01000034.1 | 63227 | 63247 | -1 | -2 | 1 | 63245 | 64478 | BV071_RS19105 | cytosine methyltransferase |
| 21.2152 | AACTGTTCAAAAGAACAAAT | NZ_BATP01000034.1 | 63227 | 63247 | 1 | -2 | 1 | 63245 | 64478 | BV071_RS19105 | cytosine methyltransferase |
| 18.8717 | AGCTGTTCGCTCGAACAAAA | NZ_BATP01000033.1 | 10990 | 11010 | 1 | 60 | -1 | 9898 | 10930 | BV071_RS18765 | DNA recombination/repair protein RecA |
| 18.7901 | TTTTGTTCGAGCGAACAGCT | NZ_BATP01000033.1 | 10990 | 11010 | -1 | 60 | -1 | 9898 | 10930 | BV071_RS18765 | DNA recombination/repair protein RecA |
| 17.1079 | CAGTGTTCTTAGGAACAAAC | NZ_BATP01000003.1 | 43785 | 43805 | -1 | 83 | -1 | 42793 | 43702 | BV071_RS01180 | hypothetical protein |
| 17.1079 | CAGTGTTCTTAGGAACAAAC | NZ_BATP01000003.1 | 43785 | 43805 | -1 | 11 | 1 | 43816 | 44431 | BV071_RS01185 | repressor LexA |
| 16.9346 | GTTTGTTCCTAAGAACACTG | NZ_BATP01000003.1 | 43785 | 43805 | 1 | 83 | -1 | 42793 | 43702 | BV071_RS01180 | hypothetical protein |
| 16.9346 | GTTTGTTCCTAAGAACACTG | NZ_BATP01000003.1 | 43785 | 43805 | 1 | 11 | 1 | 43816 | 44431 | BV071_RS01185 | repressor LexA |
| 16.7598 | AAGTGTTCATTTGATCATAG | NZ_BATP01000059.1 | 236624 | 236644 | -1 | 9 | -1 | 235766 | 236615 | BV071_RS30145 | hypothetical protein |
| 16.7577 | TTGTGTTCAATTGAACTTTC | NZ_BATP01000057.1 | 202668 | 202688 | 1 | 43 | -1 | 201929 | 202625 | BV071_RS28270 | hypothetical protein |
| 16.7301 | AGGTGTTCATTTGAAAAGTA | NZ_BATP01000020.1 | 68704 | 68724 | -1 | 245 | -1 | 66095 | 68459 | BV071_RS09400 | hypothetical protein |
| 16.7301 | AGGTGTTCATTTGAAAAGTA | NZ_BATP01000020.1 | 68704 | 68724 | -1 | 21 | 1 | 68745 | 69975 | BV071_RS09405 | hypothetical protein |
| 16.7157 | GAAAGTTCAATTGAACACAA | NZ_BATP01000057.1 | 202668 | 202688 | -1 | 43 | -1 | 201929 | 202625 | BV071_RS28270 | hypothetical protein |
| 16.7153 | CTATGATCAAATGAACACTT | NZ_BATP01000059.1 | 236624 | 236644 | 1 | 9 | -1 | 235766 | 236615 | BV071_RS30145 | hypothetical protein |
| 16.4921 | GAGTGTTCATAAAAACACCA | NZ_BATP01000055.1 | 21965 | 21985 | 1 | 183 | -1 | 21044 | 21782 | BV071_RS26395 | hypothetical protein |
| 16.4921 | GAGTGTTCATAAAAACACCA | NZ_BATP01000055.1 | 21965 | 21985 | 1 | -1 | 1 | 21984 | 22560 | BV071_RS26400 | hypothetical protein |
| 16.4389 | TGGTGTTTTTATGAACACTC | NZ_BATP01000055.1 | 21965 | 21985 | -1 | 183 | -1 | 21044 | 21782 | BV071_RS26395 | hypothetical protein |
| 16.4389 | TGGTGTTTTTATGAACACTC | NZ_BATP01000055.1 | 21965 | 21985 | -1 | -1 | 1 | 21984 | 22560 | BV071_RS26400 | hypothetical protein |
| 16.4054 | TACTTTTCAAATGAACACCT | NZ_BATP01000020.1 | 68704 | 68724 | 1 | 245 | -1 | 66095 | 68459 | BV071_RS09400 | hypothetical protein |
| 16.4054 | TACTTTTCAAATGAACACCT | NZ_BATP01000020.1 | 68704 | 68724 | 1 | 21 | 1 | 68745 | 69975 | BV071_RS09405 | hypothetical protein |
| 16.0707 | AACTGTTCATTAGATCAGTT | NZ_BATP01000032.1 | 131552 | 131572 | 1 | 13 | -1 | 129358 | 131539 | BV071_RS18370 | ATP-dependent DNA helicase RecQ |
| 16.0707 | AACTGTTCATTAGATCAGTT | NZ_BATP01000032.1 | 131552 | 131572 | 1 | 317 | 1 | 131889 | 132261 | BV071_RS18375 | transcriptional repressor |
| 15.9462 | AACTGATCTAATGAACAGTT | NZ_BATP01000032.1 | 131552 | 131572 | -1 | 13 | -1 | 129358 | 131539 | BV071_RS18370 | ATP-dependent DNA helicase RecQ |
| 15.9462 | AACTGATCTAATGAACAGTT | NZ_BATP01000032.1 | 131552 | 131572 | -1 | 317 | 1 | 131889 | 132261 | BV071_RS18375 | transcriptional repressor |
| 15.4443 | TGGTGTTCACGTGAACCCAT | NZ_BATP01000035.1 | 35814 | 35834 | 1 | 100 | -1 | 35033 | 35714 | BV071_RS19570 | hypothetical protein |
| 15.4443 | TGGTGTTCACGTGAACCCAT | NZ_BATP01000035.1 | 35814 | 35834 | 1 | -20 | 1 | 35803 | 36697 | BV071_RS19575 | hypothetical protein |
| 14.8835 | AGTTGTTCATCGGAACATGT | NZ_BATP01000053.1 | 17089 | 17109 | -1 | 12 | -1 | 16696 | 17077 | BV071_RS26005 | hypothetical protein |
| 14.8835 | AGTTGTTCATCGGAACATGT | NZ_BATP01000053.1 | 17089 | 17109 | -1 | 67 | 1 | 17176 | 18049 | BV071_RS33690 | hypothetical protein |
| 14.7902 | ATGGGTTCACGTGAACACCA | NZ_BATP01000035.1 | 35814 | 35834 | -1 | 100 | -1 | 35033 | 35714 | BV071_RS19570 | hypothetical protein |
| 14.7902 | ATGGGTTCACGTGAACACCA | NZ_BATP01000035.1 | 35814 | 35834 | -1 | -20 | 1 | 35803 | 36697 | BV071_RS19575 | hypothetical protein |
| 14.6061 | ACATGTTCCGATGAACAACT | NZ_BATP01000053.1 | 17089 | 17109 | 1 | 12 | -1 | 16696 | 17077 | BV071_RS26005 | hypothetical protein |
| 14.6061 | ACATGTTCCGATGAACAACT | NZ_BATP01000053.1 | 17089 | 17109 | 1 | 67 | 1 | 17176 | 18049 | BV071_RS33690 | hypothetical protein |
| 13.9358 | CAATGTTCATACGATCATTG | NZ_BATP01000038.1 | 21867 | 21887 | -1 | 70 | -1 | 21422 | 21797 | BV071_RS20560 | hypothetical protein |
| 13.8923 | CAATGATCGTATGAACATTG | NZ_BATP01000038.1 | 21867 | 21887 | 1 | 70 | -1 | 21422 | 21797 | BV071_RS20560 | hypothetical protein |
| 13.8125 | TGGTGTTCAACAAAGCAGTG | NZ_BATP01000060.1 | 246733 | 246753 | 1 | 51 | -1 | 245881 | 246682 | BV071_RS31395 | hypothetical protein |
| 13.7261 | TACTGTCCATGCGAACAGCA | NZ_BATP01000023.1 | 39138 | 39158 | -1 | -1 | -1 | 37897 | 39139 | BV071_RS11990 | putative DNA modification/repair radical SAM protein |
| 13.7151 | CGCTGTTCGGATAAACAGAG | NZ_BATP01000003.1 | 481785 | 481805 | 1 | 46 | -1 | 480194 | 481739 | BV071_RS32405 | hypothetical protein |
| 13.6038 | CTCTGTTTATCCGAACAGCG | NZ_BATP01000003.1 | 481785 | 481805 | -1 | 46 | -1 | 480194 | 481739 | BV071_RS32405 | hypothetical protein |
| 13.5672 | CACTGCTTTGTTGAACACCA | NZ_BATP01000060.1 | 246733 | 246753 | -1 | 51 | -1 | 245881 | 246682 | BV071_RS31395 | hypothetical protein |
| 13.4881 | AATTGTTCACATGAGCCATT | NZ_BATP01000063.1 | 3362 | 3382 | -1 | 133 | -1 | 391 | 3229 | BV071_RS32135 | 23S ribosomal RNA |
| 13.3206 | CAGTGTTCACCTGAACTCGT | NZ_BATP01000058.1 | 120444 | 120464 | -1 | -2 | -1 | 120197 | 120446 | BV071_RS29000 | hypothetical protein |
| 13.2749 | ACGAGTTCAGGTGAACACTG | NZ_BATP01000058.1 | 120444 | 120464 | 1 | -2 | -1 | 120197 | 120446 | BV071_RS29000 | hypothetical protein |
| 13.2721 | TTCTGTTCATTTGAATAGAC | NZ_BATP01000057.1 | 1357 | 1377 | 1 | 101 | -1 | 365 | 1256 | BV071_RS27425 | hypothetical protein |
| 13.2721 | TTCTGTTCATTTGAATAGAC | NZ_BATP01000057.1 | 1357 | 1377 | 1 | 700 | 1 | 2077 | 3886 | BV071_RS33810 | hypothetical protein |
| 13.2229 | GTCTATTCAAATGAACAGAA | NZ_BATP01000057.1 | 1357 | 1377 | -1 | 101 | -1 | 365 | 1256 | BV071_RS27425 | hypothetical protein |
| 13.2229 | GTCTATTCAAATGAACAGAA | NZ_BATP01000057.1 | 1357 | 1377 | -1 | 700 | 1 | 2077 | 3886 | BV071_RS33810 | hypothetical protein |
| 13.0561 | ATGAGTTCATCTGAACAAGT | NZ_BATP01000022.1 | 358291 | 358311 | -1 | -20 | -1 | 356181 | 358311 | BV071_RS11655 | recombinase RecQ |
| 13.0561 | ATGAGTTCATCTGAACAAGT | NZ_BATP01000022.1 | 358291 | 358311 | -1 | 137 | 1 | 358448 | 360548 | BV071_RS11660 | hypothetical protein |
| 12.981 | ACTTGTTCAGATGAACTCAT | NZ_BATP01000022.1 | 358291 | 358311 | 1 | -20 | -1 | 356181 | 358311 | BV071_RS11655 | recombinase RecQ |
| 12.981 | ACTTGTTCAGATGAACTCAT | NZ_BATP01000022.1 | 358291 | 358311 | 1 | 137 | 1 | 358448 | 360548 | BV071_RS11660 | hypothetical protein |
| **Opitutus terrae PB90-1** | | | | | | | | | | | |
| **score** | **site** | **chromid** | **start** | **end** | **strand** | **distance** | **gene_strand** | **gene_start** | **gene_end** | **gene_locus_tag** | **gene_product** |
| 21.5965 | ATGTGTTCAAGAGAACACAT | NC_010571.1 | 5260386 | 5260406 | -1 | 5 | -1 | 5259778 | 5260381 | OTER_RS20480 | repressor LexA |
| 21.5944 | ATGTGTTCTCTTGAACACAT | NC_010571.1 | 5260386 | 5260406 | 1 | 5 | -1 | 5259778 | 5260381 | OTER_RS20480 | repressor LexA |
| 20.3256 | CTGTGTTCAGGTGAACACAT | NC_010571.1 | 1782342 | 1782362 | -1 | -10 | -1 | 1781272 | 1782352 | OTER_RS07185 | radical SAM protein |
| 20.2667 | ATGTGTTCACCTGAACACAG | NC_010571.1 | 1782342 | 1782362 | 1 | -10 | -1 | 1781272 | 1782352 | OTER_RS07185 | radical SAM protein |
| 15.1809 | AGGTGTTCGTCCGAACAGGG | NC_010571.1 | 671361 | 671381 | -1 | 9 | 1 | 671390 | 674783 | OTER_RS23550 | immunoglobulin I-set domain-containing protein |
| 15.1054 | GGCTGTTCGCTTGAACATGT | NC_010571.1 | 5260402 | 5260422 | -1 | 21 | -1 | 5259778 | 5260381 | OTER_RS20480 | repressor LexA |
| 15.0842 | ACATGTTCAAGCGAACAGCC | NC_010571.1 | 5260402 | 5260422 | 1 | 21 | -1 | 5259778 | 5260381 | OTER_RS20480 | repressor LexA |
| 14.9998 | CCCTGTTCGGACGAACACCT | NC_010571.1 | 671361 | 671381 | 1 | 9 | 1 | 671390 | 674783 | OTER_RS23550 | immunoglobulin I-set domain-containing protein |
| 13.5872 | TTGTGTTCCTGAAAACAGAC | NC_010571.1 | 718979 | 718999 | -1 | 54 | -1 | 715238 | 718925 | OTER_RS02990 | hypothetical protein |
| 13.5622 | AGTTGCTCCCGCGAACATTA | NC_010571.1 | 4408896 | 4408916 | 1 | 251 | 1 | 4409167 | 4410229 | OTER_RS17050 | RpoD family RNA polymerase sigma factor |
| 13.5384 | TAATGTTCGCGGGAGCAACT | NC_010571.1 | 4408896 | 4408916 | -1 | 251 | 1 | 4409167 | 4410229 | OTER_RS17050 | RpoD family RNA polymerase sigma factor |
| 13.4664 | GTCTGTTTTCAGGAACACAA | NC_010571.1 | 718979 | 718999 | 1 | 54 | -1 | 715238 | 718925 | OTER_RS02990 | hypothetical protein |
| 13.3013 | CGCTCTTCGAGTGAACACTT | NC_010571.1 | 4855956 | 4855976 | 1 | 227 | -1 | 4855654 | 4855729 | OTER_RS18845 | tRNA-Met |
| 13.3013 | CGCTCTTCGAGTGAACACTT | NC_010571.1 | 4855956 | 4855976 | 1 | -10 | 1 | 4855966 | 4857451 | OTER_RS24450 | hypothetical protein |
| 13.2816 | AAGTGTTCACTCGAAGAGCG | NC_010571.1 | 4855956 | 4855976 | -1 | 227 | -1 | 4855654 | 4855729 | OTER_RS18845 | tRNA-Met |
| 13.2816 | AAGTGTTCACTCGAAGAGCG | NC_010571.1 | 4855956 | 4855976 | -1 | -10 | 1 | 4855966 | 4857451 | OTER_RS24450 | hypothetical protein |
| **Opitutaceae bacterium TAV5** | | | | | | | | | | | |
| **score** | **site** | **chromid** | **start** | **end** | **strand** | **distance** | **gene_strand** | **gene_start** | **gene_end** | **gene_locus_tag** | **gene_product** |
| 23.8732 | ATGTGTTCACTTGAACACTA | NZ_CP007053.1 | 6472599 | 6472619 | 1 | 466 | -1 | 6470207 | 6472133 | OPIT5_RS25720 | autotransporter-associated beta strand repeat protein |
| 23.8732 | ATGTGTTCACTTGAACACTA | NZ_CP007053.1 | 6472599 | 6472619 | 1 | 33 | 1 | 6472652 | 6473282 | OPIT5_RS25725 | repressor LexA |
| 23.7886 | TAGTGTTCAAGTGAACACAT | NZ_CP007053.1 | 6472599 | 6472619 | -1 | 466 | -1 | 6470207 | 6472133 | OPIT5_RS25720 | autotransporter-associated beta strand repeat protein |
| 23.7886 | TAGTGTTCAAGTGAACACAT | NZ_CP007053.1 | 6472599 | 6472619 | -1 | 33 | 1 | 6472652 | 6473282 | OPIT5_RS25725 | repressor LexA |
| 23.0551 | ATGTGTTCATTCGAACACTT | NZ_CP007053.1 | 5530019 | 5530039 | 1 | 34 | 1 | 5530073 | 5530688 | OPIT5_RS22040 | repressor LexA |
| 22.9192 | AAGTGTTCGAATGAACACAT | NZ_CP007053.1 | 5530019 | 5530039 | -1 | 34 | 1 | 5530073 | 5530688 | OPIT5_RS22040 | repressor LexA |
| 18.8535 | AATTGTTTTTTTGAACAGAA | NZ_CP007053.1 | 1221230 | 1221250 | 1 | 34 | -1 | 1219987 | 1221196 | OPIT5_RS04975 | multidrug ABC transporter substrate-binding protein |
| 18.8535 | AATTGTTTTTTTGAACAGAA | NZ_CP007053.1 | 3120809 | 3120829 | 1 | 461 | -1 | 3120153 | 3120348 | OPIT5_RS12510 | hypothetical protein |
| 18.8535 | AATTGTTTTTTTGAACAGAA | NZ_CP007053.1 | 6664491 | 6664511 | -1 | 36 | 1 | 6664547 | 6665552 | OPIT5_RS26435 | HPr(Ser) kinase/phosphatase |
| 18.8387 | TTCTGTTCAAAAAAACAATT | NZ_CP007053.1 | 1221230 | 1221250 | -1 | 34 | -1 | 1219987 | 1221196 | OPIT5_RS04975 | multidrug ABC transporter substrate-binding protein |
| 18.8387 | TTCTGTTCAAAAAAACAATT | NZ_CP007053.1 | 3120809 | 3120829 | -1 | 461 | -1 | 3120153 | 3120348 | OPIT5_RS12510 | hypothetical protein |
| 18.8387 | TTCTGTTCAAAAAAACAATT | NZ_CP007053.1 | 6664491 | 6664511 | 1 | 36 | 1 | 6664547 | 6665552 | OPIT5_RS26435 | HPr(Ser) kinase/phosphatase |
| 15.8729 | TCATGTTCATTAGAGCATTT | NZ_CP007053.1 | 4142000 | 4142020 | -1 | 29 | -1 | 4141485 | 4141971 | OPIT5_RS16525 | hypothetical protein |
| 15.7537 | AAATGCTCTAATGAACATGA | NZ_CP007053.1 | 4142000 | 4142020 | 1 | 29 | -1 | 4141485 | 4141971 | OPIT5_RS16525 | hypothetical protein |
| 15.3734 | ATATGTTCACTCAAGCACTT | NZ_CP007053.1 | 5638505 | 5638525 | -1 | 52 | -1 | 5637781 | 5638453 | OPIT5_RS22440 | prepilin-type N-terminal cleavage/methylation domain-containing protein |
| 15.204 | AAGTGCTTGAGTGAACATAT | NZ_CP007053.1 | 5638505 | 5638525 | 1 | 52 | -1 | 5637781 | 5638453 | OPIT5_RS22440 | prepilin-type N-terminal cleavage/methylation domain-containing protein |
| 14.8988 | GTTTGTTCAATAGAGCAGAC | NZ_CP007053.1 | 5245948 | 5245968 | -1 | 168 | 1 | 5246136 | 5246433 | OPIT5_RS20900 | transcriptional regulator |
| 14.8291 | CGATGTTTAGTGGAACAGTT | NZ_CP007053.1 | 2641132 | 2641152 | -1 | 3 | -1 | 2640082 | 2641129 | OPIT5_RS10560 | LacI family transcriptional regulator |
| 14.8291 | CGATGTTTAGTGGAACAGTT | NZ_CP007053.1 | 2641132 | 2641152 | -1 | 66 | 1 | 2641218 | 2641866 | OPIT5_RS10565 | prepilin-type N-terminal cleavage/methylation domain-containing protein |
| 14.7911 | GTCTGCTCTATTGAACAAAC | NZ_CP007053.1 | 5245948 | 5245968 | 1 | 168 | 1 | 5246136 | 5246433 | OPIT5_RS20900 | transcriptional regulator |
| 14.5371 | AACTGTTCCACTAAACATCG | NZ_CP007053.1 | 2641132 | 2641152 | 1 | 3 | -1 | 2640082 | 2641129 | OPIT5_RS10560 | LacI family transcriptional regulator |
| 14.5371 | AACTGTTCCACTAAACATCG | NZ_CP007053.1 | 2641132 | 2641152 | 1 | 66 | 1 | 2641218 | 2641866 | OPIT5_RS10565 | prepilin-type N-terminal cleavage/methylation domain-containing protein |
| 14.4572 | CCGTGTTCGTCAGAACACGG | NZ_CP007053.1 | 5599934 | 5599954 | -1 | 223 | -1 | 5598907 | 5599711 | OPIT5_RS22270 | aldolase |
| 14.4572 | CCGTGTTCGTCAGAACACGG | NZ_CP007053.1 | 5599934 | 5599954 | -1 | 398 | 1 | 5600352 | 5601237 | OPIT5_RS22275 | hypothetical protein |
| 14.4027 | CCGTGTTCTGACGAACACGG | NZ_CP007053.1 | 5599934 | 5599954 | 1 | 223 | -1 | 5598907 | 5599711 | OPIT5_RS22270 | aldolase |
| 14.4027 | CCGTGTTCTGACGAACACGG | NZ_CP007053.1 | 5599934 | 5599954 | 1 | 398 | 1 | 5600352 | 5601237 | OPIT5_RS22275 | hypothetical protein |
| 13.8309 | TTCTGTTCAAATGAGCCATG | NZ_CP007053.1 | 2768106 | 2768126 | 1 | -3 | 1 | 2768123 | 2768951 | OPIT5_RS11090 | 16S rRNA methyltransferase |
| 13.5333 | AAATATTCATCAGAACAATA | NZ_CP007053.1 | 1064480 | 1064500 | 1 | 2386 | -1 | 1061779 | 1062094 | OPIT5_RS04385 | hypothetical protein |
| 13.4269 | CATGGCTCATTTGAACAGAA | NZ_CP007053.1 | 2768106 | 2768126 | -1 | -3 | 1 | 2768123 | 2768951 | OPIT5_RS11090 | 16S rRNA methyltransferase |
| 13.4186 | TATTGTTCTGATGAATATTT | NZ_CP007053.1 | 1064480 | 1064500 | -1 | 2386 | -1 | 1061779 | 1062094 | OPIT5_RS04385 | hypothetical protein |
| 13.0955 | GATTGTTCAGTGGAACCAAT | NZ_CP007053.1 | 6385845 | 6385865 | -1 | 319 | 1 | 6386184 | 6386421 | OPIT5_RS25415 | hypothetical protein |
| 13.0161 | GAATGTTAACGTGAACATTT | NZ_CP007053.1 | 3403000 | 3403020 | 1 | -18 | -1 | 3401998 | 3403018 | OPIT5_RS13530 | LacI family transcriptional regulator |
| 13.0161 | GAATGTTAACGTGAACATTT | NZ_CP007053.1 | 3403000 | 3403020 | 1 | 45 | 1 | 3403065 | 3404463 | OPIT5_RS13535 | hypothetical protein |
| **Pedosphaera parvula Ellin514** | | | | | | | | | | | |
| **score** | **site** | **chromid** | **start** | **end** | **strand** | **distance** | **gene_strand** | **gene_start** | **gene_end** | **gene_locus_tag** | **gene_product** |
| 16.7794 | GTGTGTTCGTTAGAGCAATA | NZ_ABOX02000006.1 | 30836 | 30856 | 1 | 50 | -1 | 30348 | 30786 | CFLAV_RS06130 | hypothetical protein |
| 16.7794 | GTGTGTTCGTTAGAGCAATA | NZ_ABOX02000006.1 | 30836 | 30856 | 1 | 53 | 1 | 30909 | 31551 | CFLAV_RS06135 | TetR family transcriptional regulator |
| 16.5914 | TATTGCTCTAACGAACACAC | NZ_ABOX02000006.1 | 30836 | 30856 | -1 | 50 | -1 | 30348 | 30786 | CFLAV_RS06130 | hypothetical protein |
| 16.5914 | TATTGCTCTAACGAACACAC | NZ_ABOX02000006.1 | 30836 | 30856 | -1 | 53 | 1 | 30909 | 31551 | CFLAV_RS06135 | TetR family transcriptional regulator |
| 16.35 | TCTTGTTCATTGAAACAGTT | NZ_ABOX02000017.1 | 41331 | 41351 | 1 | -10 | -1 | 40873 | 41341 | CFLAV_RS14755 | tRNA-specific adenosine deaminase |
| 15.9678 | AACTGTTTCAATGAACAAGA | NZ_ABOX02000017.1 | 41331 | 41351 | -1 | -10 | -1 | 40873 | 41341 | CFLAV_RS14755 | tRNA-specific adenosine deaminase |
| 14.9349 | TGTTGTTCATGTGTACACGT | NZ_ABOX02000024.1 | 39908 | 39928 | 1 | -10 | -1 | 37779 | 39918 | CFLAV_RS18050 | elongation factor G |
| 14.8726 | ACGTGTACACATGAACAACA | NZ_ABOX02000024.1 | 39908 | 39928 | -1 | -10 | -1 | 37779 | 39918 | CFLAV_RS18050 | elongation factor G |
| 14.6215 | CAATGTTCTCTCGAGCAGTC | NZ_ABOX02000001.1 | 18545 | 18565 | -1 | 776 | -1 | 17442 | 17769 | CFLAV_RS33600 | BON domain-containing protein |
| 14.5747 | CTTTGTTCAGCGAAACAAAA | NZ_ABOX02000011.1 | 5971 | 5991 | -1 | 162 | -1 | 3742 | 5809 | CFLAV_RS10570 | alpha-tubulin suppressor |
| 14.5747 | CTTTGTTCAGCGAAACAAAA | NZ_ABOX02000011.1 | 5971 | 5991 | -1 | 212 | 1 | 6203 | 8828 | CFLAV_RS32160 | multi-sensor hybrid histidine kinase |
| 14.5409 | AATTGTTCACTTGAAGAGCA | NZ_ABOX02000036.1 | 53143 | 53163 | 1 | 35 | 1 | 53198 | 53813 | CFLAV_RS22430 | transcriptional regulator |
| 14.5325 | TGCTCTTCAAGTGAACAATT | NZ_ABOX02000036.1 | 53143 | 53163 | -1 | 35 | 1 | 53198 | 53813 | CFLAV_RS22430 | transcriptional regulator |
| 14.457 | GACTGCTCGAGAGAACATTG | NZ_ABOX02000001.1 | 18545 | 18565 | 1 | 776 | -1 | 17442 | 17769 | CFLAV_RS33600 | BON domain-containing protein |
| 14.3989 | CAGTGTTCGATTAACCAGGA | NZ_ABOX02000055.1 | 13542 | 13562 | 1 | 50 | -1 | 12691 | 13492 | CFLAV_RS27130 | prepilin-type N-terminal cleavage/methylation domain-containing protein |
| 14.3989 | CAGTGTTCGATTAACCAGGA | NZ_ABOX02000055.1 | 13542 | 13562 | 1 | 184 | 1 | 13746 | 14358 | CFLAV_RS27135 | DNA-binding protein |
| 14.2417 | TTTTGTTTCGCTGAACAAAG | NZ_ABOX02000011.1 | 5971 | 5991 | 1 | 162 | -1 | 3742 | 5809 | CFLAV_RS10570 | alpha-tubulin suppressor |
| 14.2417 | TTTTGTTTCGCTGAACAAAG | NZ_ABOX02000011.1 | 5971 | 5991 | 1 | 212 | 1 | 6203 | 8828 | CFLAV_RS32160 | multi-sensor hybrid histidine kinase |
| 14.0515 | TGGTGTTGTTGTGAACACTT | NZ_ABOX02000024.1 | 9469 | 9489 | -1 | 80 | 1 | 9569 | 10898 | CFLAV_RS17950 | NADH-dependent dehydrogenase |
| 13.9434 | AAGTGTTCACAACAACACCA | NZ_ABOX02000024.1 | 9469 | 9489 | 1 | 80 | 1 | 9569 | 10898 | CFLAV_RS17950 | NADH-dependent dehydrogenase |
| 13.8804 | TCCTGGTTAATCGAACACTG | NZ_ABOX02000055.1 | 13542 | 13562 | -1 | 50 | -1 | 12691 | 13492 | CFLAV_RS27130 | prepilin-type N-terminal cleavage/methylation domain-containing protein |
| 13.8804 | TCCTGGTTAATCGAACACTG | NZ_ABOX02000055.1 | 13542 | 13562 | -1 | 184 | 1 | 13746 | 14358 | CFLAV_RS27135 | DNA-binding protein |
| 13.655 | CTCTGTTCATGTGAACCCGA | NZ_ABOX02000041.1 | 54820 | 54840 | 1 | -10 | -1 | 51497 | 54830 | CFLAV_RS23860 | hypothetical protein |
| 13.5207 | AATTGTTCAAGGAACCACCA | NZ_ABOX02000004.1 | 200410 | 200430 | 1 | 148 | -1 | 198459 | 200262 | CFLAV_RS04705 | hypothetical protein |
| 13.497 | TGGTTTTCGTTTGAACAGCG | NZ_ABOX02000007.1 | 38233 | 38253 | 1 | 39 | -1 | 37294 | 38194 | CFLAV_RS07220 | hypothetical protein |
| 13.4371 | CGCTGTTCAAACGAAAACCA | NZ_ABOX02000007.1 | 38233 | 38253 | -1 | 39 | -1 | 37294 | 38194 | CFLAV_RS07220 | hypothetical protein |
| 13.4278 | ATTTGTACACTAGACCATTA | NZ_ABOX02000067.1 | 2419 | 2439 | 1 | 13 | -1 | 2016 | 2406 | CFLAV_RS28925 | hypothetical protein |
| 13.3995 | TAATGTTCATCTAACCAAAG | NZ_ABOX02000016.1 | 66413 | 66433 | -1 | 202 | -1 | 64597 | 66211 | CFLAV_RS14340 | hypothetical protein |
| 13.3995 | TAATGTTCATCTAACCAAAG | NZ_ABOX02000016.1 | 66413 | 66433 | -1 | 41 | 1 | 66474 | 66783 | CFLAV_RS14345 | hypothetical protein |
| 13.3689 | CAGTTTTCAATTGATCAGTA | NZ_ABOX02000017.1 | 114830 | 114850 | -1 | 234 | -1 | 114131 | 114596 | CFLAV_RS32370 | protein of unknown function UPF0040 |
| 13.3689 | CAGTTTTCAATTGATCAGTA | NZ_ABOX02000017.1 | 114830 | 114850 | -1 | 66 | 1 | 114916 | 115993 | CFLAV_RS15035 | tRNA preQ1(34) S-adenosylmethionine ribosyltransferase-isomerase QueA |
| 13.2558 | AGATGTTTATCTGAGCAGTA | NZ_ABOX02000002.1 | 75975 | 75995 | -1 | -20 | 1 | 75964 | 77272 | CFLAV_RS01655 | hypothetical protein |
| 13.2143 | GTTTGTTCATGTAAGCAAAA | NZ_ABOX02000007.1 | 22830 | 22850 | 1 | 104 | -1 | 19384 | 22726 | CFLAV_RS07175 | Immunoglobulin V-set domain protein |
| 13.2118 | ATATGTTTACTTGAACCATG | NZ_ABOX02000024.1 | 29693 | 29713 | -1 | 28 | -1 | 28675 | 29665 | CFLAV_RS18020 | bile acid:sodium symporter |
| 13.2118 | ATATGTTTACTTGAACCATG | NZ_ABOX02000024.1 | 29693 | 29713 | -1 | 173 | 1 | 29886 | 31488 | CFLAV_RS18025 | periplasmic glucan biosynthesis protein MdoG |
| 13.1653 | TACTGATCAATTGAAAACTG | NZ_ABOX02000017.1 | 114830 | 114850 | 1 | 234 | -1 | 114131 | 114596 | CFLAV_RS32370 | protein of unknown function UPF0040 |
| 13.1653 | TACTGATCAATTGAAAACTG | NZ_ABOX02000017.1 | 114830 | 114850 | 1 | 66 | 1 | 114916 | 115993 | CFLAV_RS15035 | tRNA preQ1(34) S-adenosylmethionine ribosyltransferase-isomerase QueA |
| 13.1429 | TGGTGGTTCCTTGAACAATT | NZ_ABOX02000004.1 | 200410 | 200430 | -1 | 148 | -1 | 198459 | 200262 | CFLAV_RS04705 | hypothetical protein |
| 13.0835 | TACTGCTCAGATAAACATCT | NZ_ABOX02000002.1 | 75975 | 75995 | 1 | -20 | 1 | 75964 | 77272 | CFLAV_RS01655 | hypothetical protein |
| 13.0772 | CTTTGGTTAGATGAACATTA | NZ_ABOX02000016.1 | 66413 | 66433 | 1 | 202 | -1 | 64597 | 66211 | CFLAV_RS14340 | hypothetical protein |
| 13.0772 | CTTTGGTTAGATGAACATTA | NZ_ABOX02000016.1 | 66413 | 66433 | 1 | 41 | 1 | 66474 | 66783 | CFLAV_RS14345 | hypothetical protein |
| 13.0226 | TAATGGTCTAGTGTACAAAT | NZ_ABOX02000067.1 | 2419 | 2439 | -1 | 13 | -1 | 2016 | 2406 | CFLAV_RS28925 | hypothetical protein |
| 13.0097 | TCGGGTTCACATGAACAGAG | NZ_ABOX02000041.1 | 54820 | 54840 | -1 | -10 | -1 | 51497 | 54830 | CFLAV_RS23860 | hypothetical protein |
| 13.0066 | TGGTGCTCGATCGAACTCTG | NZ_ABOX02000061.1 | 7608 | 7628 | 1 | 118 | 1 | 7746 | 8592 | CFLAV_RS28180 | glycoside hydrolase family 16 |
| 13.0063 | TTTTGCTTACATGAACAAAC | NZ_ABOX02000007.1 | 22830 | 22850 | -1 | 104 | -1 | 19384 | 22726 | CFLAV_RS07175 | Immunoglobulin V-set domain protein |
| 12.999 | TGCTGATCTTAAGAACAGTA | NZ_ABOX02000041.1 | 49347 | 49367 | 1 | 288 | -1 | 47463 | 49059 | CFLAV_RS23845 | methyl-accepting chemotaxis sensory transducer |
| 12.999 | TGCTGATCTTAAGAACAGTA | NZ_ABOX02000041.1 | 49347 | 49367 | 1 | 71 | 1 | 49438 | 50623 | CFLAV_RS23850 | hypothetical protein |
| 12.9849 | CAGAGTTCGATCGAGCACCA | NZ_ABOX02000061.1 | 7608 | 7628 | -1 | 118 | 1 | 7746 | 8592 | CFLAV_RS28180 | glycoside hydrolase family 16 |
| Rubritalea marina DSM 17716 | | | | | | | | | | | |
| score | site | chromid | start | end | strand | distance | gene_strand | gene_start | gene_end | gene_locus_tag | gene_product |
| 21.9691 | ATTTGTTCTTTTGAACACAA | NZ_KB899270.1 | 13735 | 13755 | 1 | 210 | -1 | 12481 | 13525 | F454_RS0113390 | hypothetical protein |
| 21.9691 | ATTTGTTCTTTTGAACACAA | NZ_KB899270.1 | 13735 | 13755 | 1 | 101 | 1 | 13856 | 14903 | F454_RS0113395 | DNA recombination/repair protein RecA |
| 21.919 | TTGTGTTCAAAAGAACAAAT | NZ_KB899270.1 | 13735 | 13755 | -1 | 210 | -1 | 12481 | 13525 | F454_RS0113390 | hypothetical protein |
| 21.919 | TTGTGTTCAAAAGAACAAAT | NZ_KB899270.1 | 13735 | 13755 | -1 | 101 | 1 | 13856 | 14903 | F454_RS0113395 | DNA recombination/repair protein RecA |
| 21.6349 | ATGTGTTCATATGAACAAAT | NZ_KB899247.1 | 259412 | 259432 | -1 | 347 | -1 | 256647 | 259065 | F454_RS0101090 | hypothetical protein |
| 21.6349 | ATGTGTTCATATGAACAAAT | NZ_KB899247.1 | 259412 | 259432 | -1 | 185 | 1 | 259617 | 260643 | F454_RS0101100 | twitching motility protein |
| 21.601 | ATTTGTTCATATGAACACAT | NZ_KB899247.1 | 259412 | 259432 | 1 | 347 | -1 | 256647 | 259065 | F454_RS0101090 | hypothetical protein |
| 21.601 | ATTTGTTCATATGAACACAT | NZ_KB899247.1 | 259412 | 259432 | 1 | 185 | 1 | 259617 | 260643 | F454_RS0101100 | twitching motility protein |
| 19.8787 | GTGTGTTCATTTGAACAATC | NZ_KB899249.1 | 142367 | 142387 | 1 | 9 | -1 | 141125 | 142358 | F454_RS0103370 | hypothetical protein |
| 19.7373 | GATTGTTCAAATGAACACAC | NZ_KB899249.1 | 142367 | 142387 | -1 | 9 | -1 | 141125 | 142358 | F454_RS0103370 | hypothetical protein |
| 17.6748 | CTATGTTCTTAAGAACAGTG | NZ_KB899254.1 | 155397 | 155417 | 1 | 8 | 1 | 155425 | 156040 | F454_RS0108150 | repressor LexA |
| 17.5842 | CACTGTTCTTAAGAACATAG | NZ_KB899254.1 | 155397 | 155417 | -1 | 8 | 1 | 155425 | 156040 | F454_RS0108150 | repressor LexA |
| 17.3921 | TATTGTTAGATCGAACATTA | NZ_KB899250.1 | 33612 | 33632 | 1 | 144 | -1 | 32731 | 33468 | F454_RS15350 | Maebl |
| 17.3921 | TATTGTTAGATCGAACATTA | NZ_KB899250.1 | 33612 | 33632 | 1 | -1 | 1 | 33631 | 35509 | F454_RS0103835 | hypothetical protein |
| 16.3921 | TAATGTTCGATCTAACAATA | NZ_KB899250.1 | 33612 | 33632 | -1 | 144 | -1 | 32731 | 33468 | F454_RS15350 | Maebl |
| 16.3921 | TAATGTTCGATCTAACAATA | NZ_KB899250.1 | 33612 | 33632 | -1 | -1 | 1 | 33631 | 35509 | F454_RS0103835 | hypothetical protein |
| 15.4801 | TCATGTTCAAATGACCACTC | NZ_KB899266.1 | 275 | 295 | 1 | 260 | 1 | 555 | 4062 | F454_RS0112570 | DNA polymerase III subunit alpha |
| 15.3223 | GAGTGGTCATTTGAACATGA | NZ_KB899266.1 | 275 | 295 | -1 | 260 | 1 | 555 | 4062 | F454_RS0112570 | DNA polymerase III subunit alpha |
| 15.25 | AAGTGTTACTATGAACACAT | NZ_KB899256.1 | 70483 | 70503 | 1 | -2 | 1 | 70501 | 71041 | F454_RS0109200 | hypothetical protein |
| 15.0448 | TCATGTTCACATGACCACAT | NZ_KB899270.1 | 20420 | 20440 | -1 | 286 | 1 | 20726 | 21098 | F454_RS15290 | hypothetical protein |
| 14.8869 | ATGTGGTCATGTGAACATGA | NZ_KB899270.1 | 20420 | 20440 | 1 | 286 | 1 | 20726 | 21098 | F454_RS15290 | hypothetical protein |
| 14.7862 | TCATGTTCAAATGACCACAC | NZ_KB899263.1 | 24476 | 24496 | 1 | 341 | -1 | 23928 | 24135 | F454_RS0111835 | hypothetical protein |
| 14.7862 | TCATGTTCAAATGACCACAC | NZ_KB899263.1 | 24476 | 24496 | 1 | 398 | 1 | 24894 | 25764 | F454_RS0111840 | integron integrase |
| 14.6548 | GTGTGGTCATTTGAACATGA | NZ_KB899263.1 | 24476 | 24496 | -1 | 341 | -1 | 23928 | 24135 | F454_RS0111835 | hypothetical protein |
| 14.6548 | GTGTGGTCATTTGAACATGA | NZ_KB899263.1 | 24476 | 24496 | -1 | 398 | 1 | 24894 | 25764 | F454_RS0111840 | integron integrase |
| 14.412 | ATGTGTTCATAGTAACACTT | NZ_KB899256.1 | 70483 | 70503 | -1 | -2 | 1 | 70501 | 71041 | F454_RS0109200 | hypothetical protein |
| 13.2461 | TTATGTTCTTCAGAACTCTT | NZ_KB899247.1 | 162146 | 162166 | -1 | 61 | -1 | 161359 | 162085 | F454_RS0100700 | hypothetical protein |
| 13.2175 | AAGAGTTCTGAAGAACATAA | NZ_KB899247.1 | 162146 | 162166 | 1 | 61 | -1 | 161359 | 162085 | F454_RS0100700 | hypothetical protein |
| **Verrucomicrobium spinosum DSM 4136** | | | | | | | | | | | |
| **score** | **site** | **chromid** | **start** | **end** | **strand** | **distance** | **gene_strand** | **gene_start** | **gene_end** | **gene_locus_tag** | **gene_product** |
| 23.4825 | TAGTGTTCATGTGAACACTT | NZ_ABIZ01000001.1 | 1417566 | 1417586 | 1 | -10 | 1 | 1417576 | 1418230 | VSP_RS05590 | RecA domain protein |
| 23.4597 | AAGTGTTCACATGAACACTA | NZ_ABIZ01000001.1 | 1417566 | 1417586 | -1 | -10 | 1 | 1417576 | 1418230 | VSP_RS05590 | RecA domain protein |
| 21.2158 | AAGTGTTCTTTAGAACAGAA | NZ_ABIZ01000001.1 | 4655401 | 4655421 | -1 | 848 | -1 | 4653257 | 4654553 | VSP_RS18860 | nucleotidyltransferase |
| 21.2158 | AAGTGTTCTTTAGAACAGAA | NZ_ABIZ01000001.1 | 4655401 | 4655421 | -1 | 420 | 1 | 4655841 | 4656549 | VSP_RS18870 | hypothetical protein |
| 21.0316 | TTCTGTTCTAAAGAACACTT | NZ_ABIZ01000001.1 | 4655401 | 4655421 | 1 | 848 | -1 | 4653257 | 4654553 | VSP_RS18860 | nucleotidyltransferase |
| 21.0316 | TTCTGTTCTAAAGAACACTT | NZ_ABIZ01000001.1 | 4655401 | 4655421 | 1 | 420 | 1 | 4655841 | 4656549 | VSP_RS18870 | hypothetical protein |
| 20.9499 | TAATGTTCGATTGAACAGGA | NZ_ABIZ01000001.1 | 8019138 | 8019158 | 1 | 40 | -1 | 8018060 | 8019098 | VSP_RS32310 | recombinase RecA |
| 20.9408 | TCCTGTTCAATCGAACATTA | NZ_ABIZ01000001.1 | 8019138 | 8019158 | -1 | 40 | -1 | 8018060 | 8019098 | VSP_RS32310 | recombinase RecA |
| 20.7366 | CTGTGTTCAAAAGAACAGAT | NZ_ABIZ01000001.1 | 7996531 | 7996551 | 1 | 15 | -1 | 7996138 | 7996516 | VSP_RS32200 | hemin transporter |
| 20.7366 | CTGTGTTCAAAAGAACAGAT | NZ_ABIZ01000001.1 | 7996531 | 7996551 | 1 | 175 | 1 | 7996726 | 7997353 | VSP_RS32210 | peptide-methionine (S)-S-oxide reductase |
| 20.6783 | GAGTGTTCATGTGAACATTA | NZ_ABIZ01000001.1 | 4784646 | 4784666 | -1 | 63 | -1 | 4783731 | 4784583 | VSP_RS19355 | hypothetical protein |
| 20.6783 | GAGTGTTCATGTGAACATTA | NZ_ABIZ01000001.1 | 4784646 | 4784666 | -1 | 274 | 1 | 4784940 | 4787109 | VSP_RS19360 | ferripyoverdine receptor |
| 20.632 | ATCTGTTCTTTTGAACACAG | NZ_ABIZ01000001.1 | 7996531 | 7996551 | -1 | 15 | -1 | 7996138 | 7996516 | VSP_RS32200 | hemin transporter |
| 20.632 | ATCTGTTCTTTTGAACACAG | NZ_ABIZ01000001.1 | 7996531 | 7996551 | -1 | 175 | 1 | 7996726 | 7997353 | VSP_RS32210 | peptide-methionine (S)-S-oxide reductase |
| 20.5581 | TAATGTTCACATGAACACTC | NZ_ABIZ01000001.1 | 4784646 | 4784666 | 1 | 63 | -1 | 4783731 | 4784583 | VSP_RS19355 | hypothetical protein |
| 20.5581 | TAATGTTCACATGAACACTC | NZ_ABIZ01000001.1 | 4784646 | 4784666 | 1 | 274 | 1 | 4784940 | 4787109 | VSP_RS19360 | ferripyoverdine receptor |
| 20.3922 | AAATGTTCTTGTGAACAGTA | NZ_ABIZ01000001.1 | 2177411 | 2177431 | -1 | 9 | -1 | 2176184 | 2177402 | VSP_RS08510 | DNA-directed DNA polymerase |
| 20.3922 | AAATGTTCTTGTGAACAGTA | NZ_ABIZ01000001.1 | 2177411 | 2177431 | -1 | 44 | 1 | 2177475 | 2178540 | VSP_RS39135 | hypothetical protein |
| 20.3535 | AACTGTTCATGCGAACAGTT | NZ_ABIZ01000001.1 | 6886492 | 6886512 | 1 | 93 | 1 | 6886605 | 6886923 | VSP_RS28015 | hypothetical protein |
| 20.2705 | TACTGTTCACAAGAACATTT | NZ_ABIZ01000001.1 | 2177411 | 2177431 | 1 | 9 | -1 | 2176184 | 2177402 | VSP_RS08510 | DNA-directed DNA polymerase |
| 20.2705 | TACTGTTCACAAGAACATTT | NZ_ABIZ01000001.1 | 2177411 | 2177431 | 1 | 44 | 1 | 2177475 | 2178540 | VSP_RS39135 | hypothetical protein |
| 20.2705 | AACTGTTCGCATGAACAGTT | NZ_ABIZ01000001.1 | 6886492 | 6886512 | -1 | 93 | 1 | 6886605 | 6886923 | VSP_RS28015 | hypothetical protein |
| 19.8191 | TTGTGTTCAAAAGAACAGAC | NZ_ABIZ01000001.1 | 5923273 | 5923293 | 1 | 392 | -1 | 5920991 | 5922881 | VSP_RS23935 | hypothetical protein |
| 19.8051 | GTCTGTTCTTTTGAACACAA | NZ_ABIZ01000001.1 | 5923273 | 5923293 | -1 | 392 | -1 | 5920991 | 5922881 | VSP_RS23935 | hypothetical protein |
| 18.5863 | TGGTGTTCTCGTGAACATTG | NZ_ABIZ01000001.1 | 2870293 | 2870313 | 1 | 198 | 1 | 2870511 | 2870727 | VSP_RS11635 | hypothetical protein |
| 18.5576 | CAATGTTCACGAGAACACCA | NZ_ABIZ01000001.1 | 2870293 | 2870313 | -1 | 198 | 1 | 2870511 | 2870727 | VSP_RS11635 | hypothetical protein |
| 16.5775 | GAGTGTTCAAATGAACTGTA | NZ_ABIZ01000001.1 | 3001260 | 3001280 | 1 | 147 | -1 | 2999958 | 3001113 | VSP_RS35105 | sulfite reductase flavoprotein subunit |
| 16.5775 | GAGTGTTCAAATGAACTGTA | NZ_ABIZ01000001.1 | 3001260 | 3001280 | 1 | 10 | 1 | 3001290 | 3002364 | VSP_RS12190 | radical SAM protein |
| 16.5153 | TACAGTTCATTTGAACACTC | NZ_ABIZ01000001.1 | 3001260 | 3001280 | -1 | 147 | -1 | 2999958 | 3001113 | VSP_RS35105 | sulfite reductase flavoprotein subunit |
| 16.5153 | TACAGTTCATTTGAACACTC | NZ_ABIZ01000001.1 | 3001260 | 3001280 | -1 | 10 | 1 | 3001290 | 3002364 | VSP_RS12190 | radical SAM protein |
| 15.9519 | TGGTGTTCAAATGAATAATT | NZ_ABIZ01000001.1 | 3760370 | 3760390 | -1 | -10 | 1 | 3760380 | 3760863 | VSP_RS39495 | HNH endonuclease |
| 15.9166 | AATTATTCATTTGAACACCA | NZ_ABIZ01000001.1 | 3760370 | 3760390 | 1 | -10 | 1 | 3760380 | 3760863 | VSP_RS39495 | HNH endonuclease |
| 15.6537 | ACCTGTTCGTTCAAACAAAA | NZ_ABIZ01000001.1 | 5776332 | 5776352 | -1 | 106 | -1 | 5774603 | 5776226 | VSP_RS37050 | protease Do |
| 15.4983 | TTTTGTTTGAACGAACAGGT | NZ_ABIZ01000001.1 | 5776332 | 5776352 | 1 | 106 | -1 | 5774603 | 5776226 | VSP_RS37050 | protease Do |
| 14.8614 | TCCTGTTCGCTGAAACAATT | NZ_ABIZ01000001.1 | 4659785 | 4659805 | 1 | 194 | -1 | 4658334 | 4659591 | VSP_RS18880 | recombinase |
| 14.8614 | TCCTGTTCGCTGAAACAATT | NZ_ABIZ01000001.1 | 4659785 | 4659805 | 1 | 331 | 1 | 4660136 | 4660994 | VSP_RS36175 | heparan N-sulfatase |
| 14.6905 | AATTGTTTCAGCGAACAGGA | NZ_ABIZ01000001.1 | 4659785 | 4659805 | -1 | 194 | -1 | 4658334 | 4659591 | VSP_RS18880 | recombinase |
| 14.6905 | AATTGTTTCAGCGAACAGGA | NZ_ABIZ01000001.1 | 4659785 | 4659805 | -1 | 331 | 1 | 4660136 | 4660994 | VSP_RS36175 | heparan N-sulfatase |
| 14.6528 | TGGTGTTTATTTGACCACCT | NZ_ABIZ01000001.1 | 8102612 | 8102632 | -1 | 389 | -1 | 8101983 | 8102223 | VSP_RS38570 | hypothetical protein |
| 14.6528 | TGGTGTTTATTTGACCACCT | NZ_ABIZ01000001.1 | 8102612 | 8102632 | -1 | 174 | 1 | 8102806 | 8108347 | VSP_RS32650 | excinuclease ABC subunit A |
| 14.6392 | CATTGTTCGCTAGAAAATTA | NZ_ABIZ01000001.1 | 646656 | 646676 | -1 | 164 | -1 | 646084 | 646492 | VSP_RS02455 | prepilin-type N-terminal cleavage/methylation domain-containing protein |
| 14.5221 | TAATTTTCTAGCGAACAATG | NZ_ABIZ01000001.1 | 646656 | 646676 | 1 | 164 | -1 | 646084 | 646492 | VSP_RS02455 | prepilin-type N-terminal cleavage/methylation domain-containing protein |
| 14.516 | CACTGTTCCGTTGACCACTC | NZ_ABIZ01000001.1 | 6458043 | 6458063 | 1 | 20 | -1 | 6456658 | 6458023 | VSP_RS26370 | hypothetical protein |
| 14.516 | CACTGTTCCGTTGACCACTC | NZ_ABIZ01000001.1 | 6458043 | 6458063 | 1 | 47 | 1 | 6458110 | 6461221 | VSP_RS26375 | glycosyl transferase group 1 |
| 14.4326 | AGGTGGTCAAATAAACACCA | NZ_ABIZ01000001.1 | 8102612 | 8102632 | 1 | 389 | -1 | 8101983 | 8102223 | VSP_RS38570 | hypothetical protein |
| 14.4326 | AGGTGGTCAAATAAACACCA | NZ_ABIZ01000001.1 | 8102612 | 8102632 | 1 | 174 | 1 | 8102806 | 8108347 | VSP_RS32650 | excinuclease ABC subunit A |
| 14.3862 | GAGTGGTCAACGGAACAGTG | NZ_ABIZ01000001.1 | 6458043 | 6458063 | -1 | 20 | -1 | 6456658 | 6458023 | VSP_RS26370 | hypothetical protein |
| 14.3862 | GAGTGGTCAACGGAACAGTG | NZ_ABIZ01000001.1 | 6458043 | 6458063 | -1 | 47 | 1 | 6458110 | 6461221 | VSP_RS26375 | glycosyl transferase group 1 |
| 13.9683 | TGGTGCACATTTGAACATCA | NZ_ABIZ01000001.1 | 4845988 | 4846008 | 1 | 57 | -1 | 4845697 | 4845931 | VSP_RS19590 | hypothetical protein |
| 13.8545 | TGATGTTCAAATGTGCACCA | NZ_ABIZ01000001.1 | 4845988 | 4846008 | -1 | 57 | -1 | 4845697 | 4845931 | VSP_RS19590 | hypothetical protein |
| 13.7095 | GACTGCTCAATGGAGCAAAA | NZ_ABIZ01000001.1 | 3579015 | 3579035 | 1 | 115 | -1 | 3578633 | 3578900 | VSP_RS14420 | hypothetical protein |
| 13.7095 | GACTGCTCAATGGAGCAAAA | NZ_ABIZ01000001.1 | 3579015 | 3579035 | 1 | 57 | 1 | 3579092 | 3581135 | VSP_RS14430 | hypothetical protein |
| 13.6887 | CAGTCTTCGAGAGAACAGAA | NZ_ABIZ01000001.1 | 1560199 | 1560219 | -1 | 22 | -1 | 1559814 | 1560177 | VSP_RS06130 | hypothetical protein |
| 13.6329 | TTTTGCTCCATTGAGCAGTC | NZ_ABIZ01000001.1 | 3579015 | 3579035 | -1 | 115 | -1 | 3578633 | 3578900 | VSP_RS14420 | hypothetical protein |
| 13.6329 | TTTTGCTCCATTGAGCAGTC | NZ_ABIZ01000001.1 | 3579015 | 3579035 | -1 | 57 | 1 | 3579092 | 3581135 | VSP_RS14430 | hypothetical protein |
| 13.5779 | AACTGTTCATTTGTACCCAG | NZ_ABIZ01000001.1 | 6120185 | 6120205 | 1 | 142 | -1 | 6118606 | 6120043 | VSP_RS24950 | ribosomal protein S12 methylthiotransferase RimO |
| 13.5779 | AACTGTTCATTTGTACCCAG | NZ_ABIZ01000001.1 | 6120185 | 6120205 | 1 | 549 | 1 | 6120754 | 6121126 | VSP_RS24955 | hypothetical protein |
| 13.553 | TTCTGTTCTCTCGAAGACTG | NZ_ABIZ01000001.1 | 1560199 | 1560219 | 1 | 22 | -1 | 1559814 | 1560177 | VSP_RS06130 | hypothetical protein |
| 13.3383 | AGATGTTCGCCAGAACAGGG | NZ_ABIZ01000001.1 | 7951807 | 7951827 | -1 | 50 | 1 | 7951877 | 7954055 | VSP_RS32015 | recombinase RecQ |
| 13.2456 | CCCTGTTCTGGCGAACATCT | NZ_ABIZ01000001.1 | 7951807 | 7951827 | 1 | 50 | 1 | 7951877 | 7954055 | VSP_RS32015 | recombinase RecQ |
| 13.0961 | CTGGGTACAAATGAACAGTT | NZ_ABIZ01000001.1 | 6120185 | 6120205 | -1 | 142 | -1 | 6118606 | 6120043 | VSP_RS24950 | ribosomal protein S12 methylthiotransferase RimO |
| 13.0961 | CTGGGTACAAATGAACAGTT | NZ_ABIZ01000001.1 | 6120185 | 6120205 | -1 | 549 | 1 | 6120754 | 6121126 | VSP_RS24955 | hypothetical protein |
| 13.0037 | CAATTTTCAATCGAGCAGAA | NZ_ABIZ01000001.1 | 4993573 | 4993593 | -1 | 3 | 1 | 4993596 | 4993959 | VSP_RS20185 | hypothetical protein |
| **Verrucomicrobium spinosum DSM 4136** | | | | | | | | | | | |
| **score** | **site** | **chromid** | **start** | **end** | **strand** | **distance** | **gene_strand** | **gene_start** | **gene_end** | **gene_locus_tag** | **gene_product** |
| 20.3556 | CCGTGTTCAAGTGAACACAT | NZ_CP011265.1 | 1448339 | 1448359 | 1 | 19 | -1 | 1447711 | 1448320 | IMCC26134_RS05330 | LexA family transcriptional regulator |
| 20.3232 | ATGTGTTCACTTGAACACGG | NZ_CP011265.1 | 1448339 | 1448359 | -1 | 19 | -1 | 1447711 | 1448320 | IMCC26134_RS05330 | LexA family transcriptional regulator |
| 16.2796 | CGGTGTTCTCTCAAACAGTT | NZ_CP011265.1 | 1304836 | 1304856 | 1 | 5875 | -1 | 1298625 | 1298961 | IMCC26134_RS04820 | hypothetical protein |
| 16.2796 | CGGTGTTCTCTCAAACAGTT | NZ_CP011265.1 | 1304836 | 1304856 | 1 | 310 | 1 | 1305166 | 1306501 | IMCC26134_RS04830 | integron integrase |
| 15.8753 | AACTGTTTGAGAGAACACCG | NZ_CP011265.1 | 1304836 | 1304856 | -1 | 5875 | -1 | 1298625 | 1298961 | IMCC26134_RS04820 | hypothetical protein |
| 15.8753 | AACTGTTTGAGAGAACACCG | NZ_CP011265.1 | 1304836 | 1304856 | -1 | 310 | 1 | 1305166 | 1306501 | IMCC26134_RS04830 | integron integrase |
| 13.0564 | AGATGTTCTTTTGAACTACA | NZ_CP011265.1 | 1904749 | 1904769 | -1 | 11 | -1 | 1904662 | 1904738 | IMCC26134_RS07125 | tRNA-Ala |
| 13.0564 | AGATGTTCTTTTGAACTACA | NZ_CP011265.1 | 2720900 | 2720920 | -1 | 11 | -1 | 2720813 | 2720889 | IMCC26134_RS10240 | tRNA-Ala |
| 12.9902 | CATTGTTCCCATGAACCACA | NZ_CP011265.1 | 1405013 | 1405033 | 1 | -10 | 1 | 1405023 | 1406622 | IMCC26134_RS05185 | hypothetical protein |
| **Verrucomicrobia bacterium LP2A** | | | | | | | | | | | |
| **score** | **site** | **chromid** | **start** | **end** | **strand** | **distance** | **gene_strand** | **gene_start** | **gene_end** | **gene_locus_tag** | **gene_product** |
| 15.2897 | CGCTGCTCACAAGAACACAA | NZ_JAFS01000001.1 | 574778 | 574798 | -1 | 69 | 1 | 574867 | 575167 | G346_RS0102835 | Clp protease ClpS |
| 15.2505 | TTGTGTTCTTGTGAGCAGCG | NZ_JAFS01000001.1 | 574778 | 574798 | 1 | 69 | 1 | 574867 | 575167 | G346_RS0102835 | Clp protease ClpS |
| 14.2139 | CGATGCTCAGGCGAACAATT | NZ_JAFS01000002.1 | 962732 | 962752 | 1 | 45 | -1 | 962462 | 962687 | G346_RS0111540 | hypothetical protein |
| 14.0274 | AATTGTTCGCCTGAGCATCG | NZ_JAFS01000002.1 | 962732 | 962752 | -1 | 45 | -1 | 962462 | 962687 | G346_RS0111540 | hypothetical protein |
| **Verrucomicrobia bacterium SCGC AAA164-L15** | | | | | | | | | | | |
| **score** | **site** | **chromid** | **start** | **end** | **strand** | **distance** | **gene_strand** | **gene_start** | **gene_end** | **gene_locus_tag** | **gene_product** |
| 20.2738 | TAGTGTTCATTGAAACAATA | NZ_HF539242.1 | 1819 | 1839 | 1 | 175 | -1 | 957 | 1644 | A164L15_RS10030 | ribulose-phosphate 3-epimerase |
| 20.2738 | TAGTGTTCATTGAAACAATA | NZ_HF539242.1 | 1819 | 1839 | 1 | 7 | 1 | 1846 | 2464 | A164L15_RS10035 | repressor LexA |
| 19.9165 | TATTGTTTCAATGAACACTA | NZ_HF539242.1 | 1819 | 1839 | -1 | 175 | -1 | 957 | 1644 | A164L15_RS10030 | ribulose-phosphate 3-epimerase |
| 19.9165 | TATTGTTTCAATGAACACTA | NZ_HF539242.1 | 1819 | 1839 | -1 | 7 | 1 | 1846 | 2464 | A164L15_RS10035 | repressor LexA |
| 19.1839 | ATGTGTTCTTAGGAACACAT | NZ_HF538679.1 | 6975 | 6995 | 1 | 345 | -1 | 6108 | 6630 | A164L15_RS00675 | hypothetical protein |
| 19.1839 | ATGTGTTCTTAGGAACACAT | NZ_HF538679.1 | 6975 | 6995 | 1 | 113 | 1 | 7108 | 9917 | A164L15_RS00680 | hypothetical protein |
| 19.077 | ATGTGTTCCTAAGAACACAT | NZ_HF538679.1 | 6975 | 6995 | -1 | 345 | -1 | 6108 | 6630 | A164L15_RS00675 | hypothetical protein |
| 19.077 | ATGTGTTCCTAAGAACACAT | NZ_HF538679.1 | 6975 | 6995 | -1 | 113 | 1 | 7108 | 9917 | A164L15_RS00680 | hypothetical protein |
| 17.2242 | TAGTGTTCATTTGAACGATG | NZ_HF539195.1 | 24080 | 24100 | 1 | -3 | 1 | 24097 | 25378 | A164L15_RS09495 | putative DNA modification/repair radical SAM protein |
| 17.1866 | CATCGTTCAAATGAACACTA | NZ_HF539195.1 | 24080 | 24100 | -1 | -3 | 1 | 24097 | 25378 | A164L15_RS09495 | putative DNA modification/repair radical SAM protein |
| 16.7695 | ATTTGTTCTTTTAAACAGCT | NZ_HF539074.1 | 862 | 882 | 1 | 527 | -1 | 0 | 335 | A164L15_RS07815 | multidrug ABC transporter ATPase |
| 16.7695 | ATTTGTTCTTTTAAACAGCT | NZ_HF539074.1 | 862 | 882 | 1 | 95 | 1 | 977 | 1355 | A164L15_RS07820 | hypothetical protein |
| 16.6335 | AGCTGTTTAAAAGAACAAAT | NZ_HF539074.1 | 862 | 882 | -1 | 527 | -1 | 0 | 335 | A164L15_RS07815 | multidrug ABC transporter ATPase |
| 16.6335 | AGCTGTTTAAAAGAACAAAT | NZ_HF539074.1 | 862 | 882 | -1 | 95 | 1 | 977 | 1355 | A164L15_RS07820 | hypothetical protein |
| 16.1042 | CAGTGTTCAAAAAAGCAAAA | NZ_HF539299.1 | 17237 | 17257 | -1 | 85 | -1 | 16853 | 17152 | A164L15_RS11200 | hypothetical protein |
| 16.1042 | CAGTGTTCAAAAAAGCAAAA | NZ_HF539299.1 | 17237 | 17257 | -1 | 75 | 1 | 17332 | 18379 | A164L15_RS11205 | DNA recombination/repair protein RecA |
| 15.9366 | TTTTGCTTTTTTGAACACTG | NZ_HF539299.1 | 17237 | 17257 | 1 | 85 | -1 | 16853 | 17152 | A164L15_RS11200 | hypothetical protein |
| 15.9366 | TTTTGCTTTTTTGAACACTG | NZ_HF539299.1 | 17237 | 17257 | 1 | 75 | 1 | 17332 | 18379 | A164L15_RS11205 | DNA recombination/repair protein RecA |
| 14.9404 | CTTTGTTATTTTGAACAAAT | NZ_HF539006.1 | 4923 | 4943 | 1 | 88 | -1 | 3836 | 4835 | A164L15_RS06725 | hypothetical protein |
| 14.8576 | TTGTTTTCATAGGAACAAAA | NZ_HF539117.1 | 14020 | 14040 | 1 | -10 | -1 | 13349 | 14030 | A164L15_RS08615 | hypothetical protein |
| 14.8576 | TTGTTTTCATAGGAACAAAA | NZ_HF539117.1 | 14020 | 14040 | 1 | 211 | 1 | 14251 | 14770 | A164L15_RS08620 | peptide-methionine (R)-S-oxide reductase |
| 14.6881 | TTTTGTTCCTATGAAAACAA | NZ_HF539117.1 | 14020 | 14040 | -1 | -10 | -1 | 13349 | 14030 | A164L15_RS08615 | hypothetical protein |
| 14.6881 | TTTTGTTCCTATGAAAACAA | NZ_HF539117.1 | 14020 | 14040 | -1 | 211 | 1 | 14251 | 14770 | A164L15_RS08620 | peptide-methionine (R)-S-oxide reductase |
| 14.6595 | CTCTGTTTACTCGACCACTA | NZ_HF538801.1 | 28497 | 28517 | 1 | 112 | -1 | 28124 | 28385 | A164L15_RS03305 | 30S ribosomal protein S15 |
| 14.4144 | TAGTGGTCGAGTAAACAGAG | NZ_HF538801.1 | 28497 | 28517 | -1 | 112 | -1 | 28124 | 28385 | A164L15_RS03305 | 30S ribosomal protein S15 |
| 14.2599 | CAGTGTTCTCTGGAGCAGCG | NZ_HF539178.1 | 10556 | 10576 | -1 | 95 | -1 | 8454 | 10461 | A164L15_RS09270 | acyltransferase |
| 14.2599 | CAGTGTTCTCTGGAGCAGCG | NZ_HF539178.1 | 10556 | 10576 | -1 | 220 | 1 | 10796 | 13994 | A164L15_RS09275 | carbamoyl phosphate synthase large subunit |
| 14.1274 | CGCTGCTCCAGAGAACACTG | NZ_HF539178.1 | 10556 | 10576 | 1 | 95 | -1 | 8454 | 10461 | A164L15_RS09270 | acyltransferase |
| 14.1274 | CGCTGCTCCAGAGAACACTG | NZ_HF539178.1 | 10556 | 10576 | 1 | 220 | 1 | 10796 | 13994 | A164L15_RS09275 | carbamoyl phosphate synthase large subunit |
| 13.9726 | TGCTTTTCAAGGGAACACAA | NZ_HF538945.1 | 8777 | 8797 | -1 | 29 | -1 | 7699 | 8748 | A164L15_RS05410 | beta-ketoacyl-[acyl-carrier-protein] synthase II |
| 13.8742 | TTGTGTTCCCTTGAAAAGCA | NZ_HF538945.1 | 8777 | 8797 | 1 | 29 | -1 | 7699 | 8748 | A164L15_RS05410 | beta-ketoacyl-[acyl-carrier-protein] synthase II |
| 13.872 | TGGTGCTCGGTTGAGCATTT | NZ_HF539326.1 | 14396 | 14416 | 1 | -20 | 1 | 14392 | 15551 | A164L15_RS13375 | hypothetical protein |
| 13.8291 | ATTTGTTCAAAATAACAAAG | NZ_HF539006.1 | 4923 | 4943 | -1 | 88 | -1 | 3836 | 4835 | A164L15_RS06725 | hypothetical protein |
| 13.7579 | AAATGCTCAACCGAGCACCA | NZ_HF539326.1 | 14396 | 14416 | -1 | -20 | 1 | 14392 | 15551 | A164L15_RS13375 | hypothetical protein |
| **Verrucomicrobia bacterium SCGC AAA164-O14** | | | | | | | | | | | |
| **score** | **site** | **chromid** | **start** | **end** | **strand** | **distance** | **gene_strand** | **gene_start** | **gene_end** | **gene_locus_tag** | **gene_product** |
| 22.6446 | TAGTGTTCATTTAAACACTA | NZ_HF540448.1 | 8675 | 8695 | -1 | 683 | 1 | 9378 | 9647 | A164O14_RS16650 | hypothetical protein |
| 22.4568 | TAGTGTTTAAATGAACACTA | NZ_HF540448.1 | 8675 | 8695 | 1 | 683 | 1 | 9378 | 9647 | A164O14_RS16650 | hypothetical protein |
| 20.2738 | TAGTGTTCATTGAAACAATA | NZ_HF539882.1 | 650 | 670 | -1 | 7 | -1 | 25 | 643 | A164O14_RS07620 | repressor LexA |
| 20.2738 | TAGTGTTCATTGAAACAATA | NZ_HF539882.1 | 650 | 670 | -1 | 175 | 1 | 845 | 1532 | A164O14_RS07625 | ribulose-phosphate 3-epimerase |
| 19.9165 | TATTGTTTCAATGAACACTA | NZ_HF539882.1 | 650 | 670 | 1 | 7 | -1 | 25 | 643 | A164O14_RS07620 | repressor LexA |
| 19.9165 | TATTGTTTCAATGAACACTA | NZ_HF539882.1 | 650 | 670 | 1 | 175 | 1 | 845 | 1532 | A164O14_RS07625 | ribulose-phosphate 3-epimerase |
| 17.9401 | AAGTGTTCTAATAAACAGTC | NZ_HF539351.1 | 8256 | 8276 | -1 | 29 | -1 | 7789 | 8227 | A164O14_RS00360 | 30S ribosomal protein S12 |
| 17.8446 | GACTGTTTATTAGAACACTT | NZ_HF539351.1 | 8256 | 8276 | 1 | 29 | -1 | 7789 | 8227 | A164O14_RS00360 | 30S ribosomal protein S12 |
| 17.2242 | TAGTGTTCATTTGAACGATG | NZ_HF539851.1 | 12965 | 12985 | 1 | -3 | 1 | 12982 | 14263 | A164O14_RS07270 | putative DNA modification/repair radical SAM protein |
| 17.1866 | CATCGTTCAAATGAACACTA | NZ_HF539851.1 | 12965 | 12985 | -1 | -3 | 1 | 12982 | 14263 | A164O14_RS07270 | putative DNA modification/repair radical SAM protein |
| 16.9863 | TGTTGTTAAAATGAACAATA | NZ_HF539345.1 | 1192 | 1212 | -1 | -10 | -1 | 737 | 1202 | A164O14_RS00265 | hypothetical protein |
| 16.1042 | CAGTGTTCAAAAAAGCAAAA | NZ_HF539584.1 | 9396 | 9416 | 1 | 75 | -1 | 8274 | 9321 | A164O14_RS03700 | DNA recombination/repair protein RecA |
| 16.1042 | CAGTGTTCAAAAAAGCAAAA | NZ_HF539584.1 | 9396 | 9416 | 1 | 115 | 1 | 9531 | 9720 | A164O14_RS03705 | hypothetical protein |
| 15.9533 | TATTGTTCATTTTAACAACA | NZ_HF539345.1 | 1192 | 1212 | 1 | -10 | -1 | 737 | 1202 | A164O14_RS00265 | hypothetical protein |
| 15.9366 | TTTTGCTTTTTTGAACACTG | NZ_HF539584.1 | 9396 | 9416 | -1 | 75 | -1 | 8274 | 9321 | A164O14_RS03700 | DNA recombination/repair protein RecA |
| 15.9366 | TTTTGCTTTTTTGAACACTG | NZ_HF539584.1 | 9396 | 9416 | -1 | 115 | 1 | 9531 | 9720 | A164O14_RS03705 | hypothetical protein |
| 14.8576 | TTGTTTTCATAGGAACAAAA | NZ_HF539416.1 | 17959 | 17979 | 1 | -10 | -1 | 17288 | 17969 | A164O14_RS01155 | hypothetical protein |
| 14.8576 | TTGTTTTCATAGGAACAAAA | NZ_HF539416.1 | 17959 | 17979 | 1 | 211 | 1 | 18190 | 18709 | A164O14_RS01160 | peptide-methionine (R)-S-oxide reductase |
| 14.6881 | TTTTGTTCCTATGAAAACAA | NZ_HF539416.1 | 17959 | 17979 | -1 | -10 | -1 | 17288 | 17969 | A164O14_RS01155 | hypothetical protein |
| 14.6881 | TTTTGTTCCTATGAAAACAA | NZ_HF539416.1 | 17959 | 17979 | -1 | 211 | 1 | 18190 | 18709 | A164O14_RS01160 | peptide-methionine (R)-S-oxide reductase |
| 14.6595 | CTCTGTTTACTCGACCACTA | NZ_HF539943.1 | 1427 | 1447 | -1 | 112 | 1 | 1559 | 1820 | A164O14_RS08905 | 30S ribosomal protein S15 |
| 14.4144 | TAGTGGTCGAGTAAACAGAG | NZ_HF539943.1 | 1427 | 1447 | 1 | 112 | 1 | 1559 | 1820 | A164O14_RS08905 | 30S ribosomal protein S15 |
| 14.2599 | CAGTGTTCTCTGGAGCAGCG | NZ_HF540402.1 | 656 | 676 | -1 | 95 | -1 | 0 | 561 | A164O14_RS15520 | hypothetical protein |
| 14.2599 | CAGTGTTCTCTGGAGCAGCG | NZ_HF539636.1 | 47 | 67 | -1 | 220 | 1 | 287 | 3485 | A164O14_RS04145 | carbamoyl phosphate synthase large subunit |
| 14.1274 | CGCTGCTCCAGAGAACACTG | NZ_HF540402.1 | 656 | 676 | 1 | 95 | -1 | 0 | 561 | A164O14_RS15520 | hypothetical protein |
| 14.1274 | CGCTGCTCCAGAGAACACTG | NZ_HF539636.1 | 47 | 67 | 1 | 220 | 1 | 287 | 3485 | A164O14_RS04145 | carbamoyl phosphate synthase large subunit |
| 14.055 | TAATGTTTTTTGGAGCACAT | NZ_HF539520.1 | 468 | 488 | 1 | 92 | -1 | 0 | 376 | A164O14_RS02725 | sulfatase |
| 14.055 | TAATGTTTTTTGGAGCACAT | NZ_HF539520.1 | 468 | 488 | 1 | 9 | 1 | 497 | 1205 | A164O14_RS02730 | hypothetical protein |
| 14.0513 | ATGTGCTCCAAAAAACATTA | NZ_HF539520.1 | 468 | 488 | -1 | 92 | -1 | 0 | 376 | A164O14_RS02725 | sulfatase |
| 14.0513 | ATGTGCTCCAAAAAACATTA | NZ_HF539520.1 | 468 | 488 | -1 | 9 | 1 | 497 | 1205 | A164O14_RS02730 | hypothetical protein |
| 13.7243 | ATGAGTACAAAAGAACACTA | NZ_HF539546.1 | 449 | 469 | -1 | 11 | -1 | 0 | 438 | A164O14_RS03060 | magnesium chelatase |
| 13.7243 | ATGAGTACAAAAGAACACTA | NZ_HF539546.1 | 449 | 469 | -1 | 58 | 1 | 527 | 890 | A164O14_RS03065 | hypothetical protein |
| 13.7178 | TAGTGTTCTTTTGTACTCAT | NZ_HF539546.1 | 449 | 469 | 1 | 11 | -1 | 0 | 438 | A164O14_RS03060 | magnesium chelatase |
| 13.7178 | TAGTGTTCTTTTGTACTCAT | NZ_HF539546.1 | 449 | 469 | 1 | 58 | 1 | 527 | 890 | A164O14_RS03065 | hypothetical protein |
| **Verrucomicrobium sp. 3C** | | | | | | | | | | | |
| **score** | **site** | **chromid** | **start** | **end** | **strand** | **distance** | **gene_strand** | **gene_start** | **gene_end** | **gene_locus_tag** | **gene_product** |
| 17.2646 | CTTTGTTCGCGTGAACAAGA | NZ_KB901875.1 | 653386 | 653406 | 1 | 68 | 1 | 653474 | 654305 | A37A_RS0103850 | rhomboid family intramembrane serine protease |
| 17.2026 | TCTTGTTCACGCGAACAAAG | NZ_KB901875.1 | 653386 | 653406 | -1 | 68 | 1 | 653474 | 654305 | A37A_RS0103850 | rhomboid family intramembrane serine protease |
| **Verrucomicrobium sp. BvORR034** | | | | | | | | | | | |
| **score** | **site** | **chromid** | **start** | **end** | **strand** | **distance** | **gene_strand** | **gene_start** | **gene_end** | **gene_locus_tag** | **gene_product** |
| 24.0432 | AATTGTTCATTTGAACACTT | NZ_BATQ01000093.1 | 2740 | 2760 | -1 | 107 | -1 | 1646 | 2633 | BV034_RS15850 | DNA methyltransferase |
| 23.9963 | AAGTGTTCAAATGAACAATT | NZ_BATQ01000093.1 | 2740 | 2760 | 1 | 107 | -1 | 1646 | 2633 | BV034_RS15850 | DNA methyltransferase |
| 23.4825 | TAGTGTTCATGTGAACACTT | NZ_BATQ01000168.1 | 36194 | 36214 | -1 | -10 | -1 | 35547 | 36204 | BV034_RS29455 | hypothetical protein |
| 23.4597 | AAGTGTTCACATGAACACTA | NZ_BATQ01000168.1 | 36194 | 36214 | 1 | -10 | -1 | 35547 | 36204 | BV034_RS29455 | hypothetical protein |
| 22.8632 | ATGTGTTCATATGAACACTT | NZ_BATQ01000141.1 | 71318 | 71338 | -1 | -2 | 1 | 71336 | 71705 | BV034_RS24615 | hypothetical protein |
| 22.8367 | AAGTGTTCATATGAACACAT | NZ_BATQ01000141.1 | 71318 | 71338 | 1 | -2 | 1 | 71336 | 71705 | BV034_RS24615 | hypothetical protein |
| 20.9499 | TAATGTTCGATTGAACAGGA | NZ_BATQ01000008.1 | 78808 | 78828 | -1 | 40 | 1 | 78868 | 79993 | BV034_RS01410 | DNA recombination/repair protein RecA |
| 20.9408 | TCCTGTTCAATCGAACATTA | NZ_BATQ01000008.1 | 78808 | 78828 | 1 | 40 | 1 | 78868 | 79993 | BV034_RS01410 | DNA recombination/repair protein RecA |
| 20.7703 | TAATGTTCACTTGAACAGTC | NZ_BATQ01000080.1 | 32426 | 32446 | -1 | 275 | -1 | 29973 | 32151 | BV034_RS14045 | hypothetical protein |
| 20.7703 | TAATGTTCACTTGAACAGTC | NZ_BATQ01000080.1 | 32426 | 32446 | -1 | 135 | 1 | 32581 | 33361 | BV034_RS14050 | hypothetical protein |
| 20.7611 | AACTGTTCGTGTGAACAGTA | NZ_BATQ01000036.1 | 2929 | 2949 | 1 | 93 | -1 | 2518 | 2836 | BV034_RS05920 | hypothetical protein |
| 20.7366 | CTGTGTTCAAAAGAACAGAT | NZ_BATQ01000008.1 | 84789 | 84809 | -1 | 175 | -1 | 83987 | 84614 | BV034_RS01435 | peptide-methionine (S)-S-oxide reductase |
| 20.7366 | CTGTGTTCAAAAGAACAGAT | NZ_BATQ01000008.1 | 84789 | 84809 | -1 | 15 | 1 | 84824 | 85202 | BV034_RS01440 | hemin transporter |
| 20.7114 | GACTGTTCAAGTGAACATTA | NZ_BATQ01000080.1 | 32426 | 32446 | 1 | 275 | -1 | 29973 | 32151 | BV034_RS14045 | hypothetical protein |
| 20.7114 | GACTGTTCAAGTGAACATTA | NZ_BATQ01000080.1 | 32426 | 32446 | 1 | 135 | 1 | 32581 | 33361 | BV034_RS14050 | hypothetical protein |
| 20.7035 | TACTGTTCACACGAACAGTT | NZ_BATQ01000036.1 | 2929 | 2949 | -1 | 93 | -1 | 2518 | 2836 | BV034_RS05920 | hypothetical protein |
| 20.632 | ATCTGTTCTTTTGAACACAG | NZ_BATQ01000008.1 | 84789 | 84809 | 1 | 175 | -1 | 83987 | 84614 | BV034_RS01435 | peptide-methionine (S)-S-oxide reductase |
| 20.632 | ATCTGTTCTTTTGAACACAG | NZ_BATQ01000008.1 | 84789 | 84809 | 1 | 15 | 1 | 84824 | 85202 | BV034_RS01440 | hemin transporter |
| 20.2706 | AATTGTTCGTGCGAACAGTA | NZ_BATQ01000145.1 | 20351 | 20371 | 1 | 44 | -1 | 19242 | 20307 | BV034_RS25560 | hypothetical protein |
| 20.2706 | AATTGTTCGTGCGAACAGTA | NZ_BATQ01000145.1 | 20351 | 20371 | 1 | 9 | 1 | 20380 | 21598 | BV034_RS25565 | hypothetical protein |
| 20.1203 | TACTGTTCGCACGAACAATT | NZ_BATQ01000145.1 | 20351 | 20371 | -1 | 44 | -1 | 19242 | 20307 | BV034_RS25560 | hypothetical protein |
| 20.1203 | TACTGTTCGCACGAACAATT | NZ_BATQ01000145.1 | 20351 | 20371 | -1 | 9 | 1 | 20380 | 21598 | BV034_RS25565 | hypothetical protein |
| 19.8191 | TTGTGTTCAAAAGAACAGAC | NZ_BATQ01000050.1 | 24735 | 24755 | -1 | 392 | 1 | 25147 | 27037 | BV034_RS09610 | hypothetical protein |
| 19.8051 | GTCTGTTCTTTTGAACACAA | NZ_BATQ01000050.1 | 24735 | 24755 | 1 | 392 | 1 | 25147 | 27037 | BV034_RS09610 | hypothetical protein |
| 18.5062 | TGCTGTTCAAACAAACAATA | NZ_BATQ01000036.1 | 1892 | 1912 | 1 | 31 | -1 | 1366 | 1861 | BV034_RS05910 | hypothetical protein |
| 18.5062 | TGCTGTTCAAACAAACAATA | NZ_BATQ01000036.1 | 1892 | 1912 | 1 | 253 | 1 | 2165 | 2456 | BV034_RS05915 | hypothetical protein |
| 18.4018 | TATTGTTTGTTTGAACAGCA | NZ_BATQ01000036.1 | 1892 | 1912 | -1 | 31 | -1 | 1366 | 1861 | BV034_RS05910 | hypothetical protein |
| 18.4018 | TATTGTTTGTTTGAACAGCA | NZ_BATQ01000036.1 | 1892 | 1912 | -1 | 253 | 1 | 2165 | 2456 | BV034_RS05915 | hypothetical protein |
| 17.698 | AATTATTCATTTGAACACTA | NZ_BATQ01000113.1 | 33233 | 33253 | -1 | 17 | -1 | 32760 | 33216 | BV034_RS18725 | HNH endonuclease |
| 17.6756 | ATGTGTTCAAATGAACTGTA | NZ_BATQ01000133.1 | 20721 | 20741 | -1 | 10 | -1 | 19637 | 20711 | BV034_RS22225 | radical SAM protein |
| 17.6756 | ATGTGTTCAAATGAACTGTA | NZ_BATQ01000133.1 | 20721 | 20741 | -1 | 147 | 1 | 20888 | 21710 | BV034_RS22230 | hypothetical protein |
| 17.6193 | TAGTGTTCAAATGAATAATT | NZ_BATQ01000113.1 | 33233 | 33253 | 1 | 17 | -1 | 32760 | 33216 | BV034_RS18725 | HNH endonuclease |
| 17.587 | TACAGTTCATTTGAACACAT | NZ_BATQ01000133.1 | 20721 | 20741 | 1 | 10 | -1 | 19637 | 20711 | BV034_RS22225 | radical SAM protein |
| 17.587 | TACAGTTCATTTGAACACAT | NZ_BATQ01000133.1 | 20721 | 20741 | 1 | 147 | 1 | 20888 | 21710 | BV034_RS22230 | hypothetical protein |
| 17.0359 | TAATGTTCAATGGAAAACAA | NZ_BATQ01000117.1 | 121850 | 121870 | -1 | 297 | -1 | 120866 | 121553 | BV034_RS19535 | hypothetical protein |
| 17.0359 | TAATGTTCAATGGAAAACAA | NZ_BATQ01000117.1 | 121850 | 121870 | -1 | -10 | 1 | 121860 | 122640 | BV034_RS19540 | hypothetical protein |
| 16.9608 | TTGTTTTCCATTGAACATTA | NZ_BATQ01000117.1 | 121850 | 121870 | 1 | 297 | -1 | 120866 | 121553 | BV034_RS19535 | hypothetical protein |
| 16.9608 | TTGTTTTCCATTGAACATTA | NZ_BATQ01000117.1 | 121850 | 121870 | 1 | -10 | 1 | 121860 | 122640 | BV034_RS19540 | hypothetical protein |
| 16.6127 | GAGTTTTCGATTGAACAATT | NZ_BATQ01000184.1 | 15871 | 15891 | 1 | 54 | -1 | 14629 | 15817 | BV034_RS33095 | hypothetical protein |
| 16.6074 | AATTGTTCAATCGAAAACTC | NZ_BATQ01000184.1 | 15871 | 15891 | -1 | 54 | -1 | 14629 | 15817 | BV034_RS33095 | hypothetical protein |
| 15.6537 | ACCTGTTCGTTCAAACAAAA | NZ_BATQ01000055.1 | 18298 | 18318 | 1 | 106 | 1 | 18424 | 20047 | BV034_RS10175 | hypothetical protein |
| 15.4983 | TTTTGTTTGAACGAACAGGT | NZ_BATQ01000055.1 | 18298 | 18318 | -1 | 106 | 1 | 18424 | 20047 | BV034_RS10175 | hypothetical protein |
| 14.6528 | TGGTGTTTATTTGACCACCT | NZ_BATQ01000007.1 | 87242 | 87262 | 1 | 168 | -1 | 81539 | 87074 | BV034_RS01070 | excinuclease ABC subunit A |
| 14.6528 | TGGTGTTTATTTGACCACCT | NZ_BATQ01000007.1 | 87242 | 87262 | 1 | 149 | 1 | 87411 | 87612 | BV034_RS01075 | acyl-protein synthetase |
| 14.6392 | CATTGTTCGCTAGAAAATTA | NZ_BATQ01000015.1 | 3866 | 3886 | -1 | 165 | -1 | 3293 | 3701 | BV034_RS03125 | prepilin-type N-terminal cleavage/methylation domain-containing protein |
| 14.5221 | TAATTTTCTAGCGAACAATG | NZ_BATQ01000015.1 | 3866 | 3886 | 1 | 165 | -1 | 3293 | 3701 | BV034_RS03125 | prepilin-type N-terminal cleavage/methylation domain-containing protein |
| 14.4822 | TTTTGCTCATGGGAACAGCT | NZ_BATQ01000119.1 | 51065 | 51085 | 1 | -10 | -1 | 30039 | 51075 | BV034_RS19765 | hypothetical protein |
| 14.4326 | AGGTGGTCAAATAAACACCA | NZ_BATQ01000007.1 | 87242 | 87262 | -1 | 168 | -1 | 81539 | 87074 | BV034_RS01070 | excinuclease ABC subunit A |
| 14.4326 | AGGTGGTCAAATAAACACCA | NZ_BATQ01000007.1 | 87242 | 87262 | -1 | 149 | 1 | 87411 | 87612 | BV034_RS01075 | acyl-protein synthetase |
| 14.3622 | AGCTGTTCCCATGAGCAAAA | NZ_BATQ01000119.1 | 51065 | 51085 | -1 | -10 | -1 | 30039 | 51075 | BV034_RS19765 | hypothetical protein |
| 13.6858 | CACTGTTCGATTGTGCAGTC | NZ_BATQ01000024.1 | 31309 | 31329 | 1 | 13 | -1 | 30687 | 31296 | BV034_RS04925 | repressor LexA |
| 13.6858 | CACTGTTCGATTGTGCAGTC | NZ_BATQ01000024.1 | 31309 | 31329 | 1 | 69 | 1 | 31398 | 32193 | BV034_RS04930 | hypothetical protein |
| 13.6544 | GACTGCACAATCGAACAGTG | NZ_BATQ01000024.1 | 31309 | 31329 | -1 | 13 | -1 | 30687 | 31296 | BV034_RS04925 | repressor LexA |
| 13.6544 | GACTGCACAATCGAACAGTG | NZ_BATQ01000024.1 | 31309 | 31329 | -1 | 69 | 1 | 31398 | 32193 | BV034_RS04930 | hypothetical protein |
| 13.6018 | TGCTGTTCAAGAGAACTGTG | NZ_BATQ01000050.1 | 30328 | 30348 | 1 | 22 | 1 | 30370 | 31324 | BV034_RS09625 | hypothetical protein |
| 13.563 | CACAGTTCTCTTGAACAGCA | NZ_BATQ01000050.1 | 30328 | 30348 | -1 | 22 | 1 | 30370 | 31324 | BV034_RS09625 | hypothetical protein |
| 13.5225 | AAGTGTCCAAACGACCAGTA | NZ_BATQ01000176.1 | 46888 | 46908 | -1 | 737 | 1 | 47645 | 47858 | BV034_RS31205 | hypothetical protein |
| 13.515 | CACTGTTCATGTGAATAGTG | NZ_BATQ01000125.1 | 106736 | 106756 | -1 | 37 | -1 | 106015 | 106699 | BV034_RS20915 | hypothetical protein |
| 13.4606 | CACTATTCACATGAACAGTG | NZ_BATQ01000125.1 | 106736 | 106756 | 1 | 37 | -1 | 106015 | 106699 | BV034_RS20915 | hypothetical protein |
| 13.2608 | AACTGTCTTTTTGAACATTA | NZ_BATQ01000003.1 | 29089 | 29109 | -1 | 84 | -1 | 26254 | 29005 | BV034_RS00595 | hypothetical protein |
| 13.2608 | AACTGTCTTTTTGAACATTA | NZ_BATQ01000003.1 | 29089 | 29109 | -1 | 145 | 1 | 29254 | 29752 | BV034_RS00600 | hypothetical protein |
| 13.1778 | AGATTTTCGATGGAACAGAA | NZ_BATQ01000046.1 | 63485 | 63505 | -1 | 40 | 1 | 63545 | 64010 | BV034_RS08210 | hypothetical protein |
| 13.1689 | TTATGTTCATAGGATCATTG | NZ_BATQ01000182.1 | 104125 | 104145 | -1 | 194 | 1 | 104339 | 104828 | BV034_RS32565 | hypothetical protein |
| 13.1577 | TTGTGTTCAATGAAAAACTC | NZ_BATQ01000011.1 | 636 | 656 | -1 | 66 | 1 | 722 | 1043 | BV034_RS02175 | thiol reductase thioredoxin |
| 13.104 | AGGTGTTTGATCGATCAATT | NZ_BATQ01000008.1 | 87551 | 87571 | -1 | 46 | 1 | 87617 | 88295 | BV034_RS01450 | hypothetical protein |
| 13.0826 | CAATGATCCTATGAACATAA | NZ_BATQ01000182.1 | 104125 | 104145 | 1 | 194 | 1 | 104339 | 104828 | BV034_RS32565 | hypothetical protein |
| 13.0481 | AATTGATCGATCAAACACCT | NZ_BATQ01000008.1 | 87551 | 87571 | 1 | 46 | 1 | 87617 | 88295 | BV034_RS01450 | hypothetical protein |
| 13.0359 | CGATGGTCAATGGAACATGA | NZ_BATQ01000085.1 | 72878 | 72898 | 1 | 169 | -1 | 71314 | 72709 | BV034_RS14780 | hypothetical protein |
| 13.0359 | CGATGGTCAATGGAACATGA | NZ_BATQ01000085.1 | 72878 | 72898 | 1 | 450 | 1 | 73348 | 73825 | BV034_RS14785 | hypothetical protein |
| 13.0037 | CAATTTTCAATCGAGCAGAA | NZ_BATQ01000075.1 | 14845 | 14865 | 1 | 3 | -1 | 14479 | 14842 | BV034_RS13110 | hypothetical protein |
| 13.0004 | TCATGTTCCATTGACCATCG | NZ_BATQ01000085.1 | 72878 | 72898 | -1 | 169 | -1 | 71314 | 72709 | BV034_RS14780 | hypothetical protein |
| 13.0004 | TCATGTTCCATTGACCATCG | NZ_BATQ01000085.1 | 72878 | 72898 | -1 | 450 | 1 | 73348 | 73825 | BV034_RS14785 | hypothetical protein |
| **Verrucomicrobium sp. BvORR034** | | | | | | | | | | | |
| **score** | **site** | **chromid** | **start** | **end** | **strand** | **distance** | **gene_strand** | **gene_start** | **gene_end** | **gene_locus_tag** | **gene_product** |
| 24.0561 | TAGTGTTCACTCGAACACTA | NZ_BATR01000109.1 | 2670 | 2690 | -1 | 7 | -1 | 2015 | 2663 | BV106_RS19380 | hypothetical protein |
| 24.001 | TAGTGTTCGAGTGAACACTA | NZ_BATR01000109.1 | 2670 | 2690 | 1 | 7 | -1 | 2015 | 2663 | BV106_RS19380 | hypothetical protein |
| 23.6454 | AAGTGTTCAAGTGAACAGTA | NZ_BATR01000122.1 | 40396 | 40416 | 1 | 77 | 1 | 40493 | 40730 | BV106_RS22605 | hypothetical protein |
| 23.5421 | TACTGTTCACTTGAACACTT | NZ_BATR01000122.1 | 40396 | 40416 | -1 | 77 | 1 | 40493 | 40730 | BV106_RS22605 | hypothetical protein |
| 22.8468 | ATGTGTTCATGTGAACACTA | NZ_BATR01000094.1 | 133643 | 133663 | 1 | -2 | -1 | 133276 | 133645 | BV106_RS15940 | hypothetical protein |
| 22.7341 | TAGTGTTCACATGAACACAT | NZ_BATR01000094.1 | 133643 | 133663 | -1 | -2 | -1 | 133276 | 133645 | BV106_RS15940 | hypothetical protein |
| 21.5846 | TTGTGTTCAAAAGAACAGAT | NZ_BATR01000171.1 | 51621 | 51641 | 1 | 392 | -1 | 49339 | 51229 | BV106_RS30940 | hypothetical protein |
| 21.5706 | ATCTGTTCTTTTGAACACAA | NZ_BATR01000171.1 | 51621 | 51641 | -1 | 392 | -1 | 49339 | 51229 | BV106_RS30940 | hypothetical protein |
| 20.9499 | TAATGTTCGATTGAACAGGA | NZ_BATR01000041.1 | 3041 | 3061 | -1 | 40 | 1 | 3101 | 4226 | BV106_RS06355 | DNA recombination/repair protein RecA |
| 20.9408 | TCCTGTTCAATCGAACATTA | NZ_BATR01000041.1 | 3041 | 3061 | 1 | 40 | 1 | 3101 | 4226 | BV106_RS06355 | DNA recombination/repair protein RecA |
| 20.7611 | AACTGTTCGTGTGAACAGTA | NZ_BATR01000075.1 | 173249 | 173269 | 1 | 93 | -1 | 172838 | 173156 | BV106_RS11595 | hypothetical protein |
| 20.7366 | CTGTGTTCAAAAGAACAGAT | NZ_BATR01000022.1 | 29389 | 29409 | 1 | 15 | -1 | 28996 | 29374 | BV106_RS04140 | hemin transporter |
| 20.7366 | CTGTGTTCAAAAGAACAGAT | NZ_BATR01000022.1 | 29389 | 29409 | 1 | 173 | 1 | 29582 | 30209 | BV106_RS04145 | peptide-methionine (S)-S-oxide reductase |
| 20.7035 | TACTGTTCACACGAACAGTT | NZ_BATR01000075.1 | 173249 | 173269 | -1 | 93 | -1 | 172838 | 173156 | BV106_RS11595 | hypothetical protein |
| 20.632 | ATCTGTTCTTTTGAACACAG | NZ_BATR01000022.1 | 29389 | 29409 | -1 | 15 | -1 | 28996 | 29374 | BV106_RS04140 | hemin transporter |
| 20.632 | ATCTGTTCTTTTGAACACAG | NZ_BATR01000022.1 | 29389 | 29409 | -1 | 173 | 1 | 29582 | 30209 | BV106_RS04145 | peptide-methionine (S)-S-oxide reductase |
| 20.0482 | CATTGTTCTTACGAACAGTA | NZ_BATR01000176.1 | 11774 | 11794 | 1 | 44 | -1 | 10650 | 11730 | BV106_RS31335 | hypothetical protein |
| 20.0482 | CATTGTTCTTACGAACAGTA | NZ_BATR01000176.1 | 11774 | 11794 | 1 | 9 | 1 | 11803 | 13022 | BV106_RS31340 | hypothetical protein |
| 19.8935 | TACTGTTCGTAAGAACAATG | NZ_BATR01000176.1 | 11774 | 11794 | -1 | 44 | -1 | 10650 | 11730 | BV106_RS31335 | hypothetical protein |
| 19.8935 | TACTGTTCGTAAGAACAATG | NZ_BATR01000176.1 | 11774 | 11794 | -1 | 9 | 1 | 11803 | 13022 | BV106_RS31340 | hypothetical protein |
| 19.3909 | TATTGTTCATGCGAACAGTC | NZ_BATR01000146.1 | 72287 | 72307 | 1 | 61 | -1 | 71398 | 72226 | BV106_RS26435 | hypothetical protein |
| 19.3909 | TATTGTTCATGCGAACAGTC | NZ_BATR01000146.1 | 72287 | 72307 | 1 | 289 | 1 | 72596 | 74782 | BV106_RS26440 | hypothetical protein |
| 19.2755 | GACTGTTCGCATGAACAATA | NZ_BATR01000146.1 | 72287 | 72307 | -1 | 61 | -1 | 71398 | 72226 | BV106_RS26435 | hypothetical protein |
| 19.2755 | GACTGTTCGCATGAACAATA | NZ_BATR01000146.1 | 72287 | 72307 | -1 | 289 | 1 | 72596 | 74782 | BV106_RS26440 | hypothetical protein |
| 19.269 | CGCTGTTCACTCGAACAGTT | NZ_BATR01000081.1 | 46667 | 46687 | -1 | 74 | -1 | 46278 | 46593 | BV106_RS12160 | hypothetical protein |
| 19.0411 | AACTGTTCGAGTGAACAGCG | NZ_BATR01000081.1 | 46667 | 46687 | 1 | 74 | -1 | 46278 | 46593 | BV106_RS12160 | hypothetical protein |
| 18.3338 | TGGTGTACATTTGAACATAA | NZ_BATR01000016.1 | 65515 | 65535 | -1 | 255 | -1 | 64324 | 65260 | BV106_RS02785 | hypothetical protein |
| 18.1175 | TTATGTTCAAATGTACACCA | NZ_BATR01000016.1 | 65515 | 65535 | 1 | 255 | -1 | 64324 | 65260 | BV106_RS02785 | hypothetical protein |
| 18.1018 | CGCTGTTCACGCGAACACTG | NZ_BATR01000122.1 | 44150 | 44170 | 1 | 106 | -1 | 43582 | 44044 | BV106_RS22635 | hypothetical protein |
| 18.0574 | CAGTGTTCGCGTGAACAGCG | NZ_BATR01000122.1 | 44150 | 44170 | -1 | 106 | -1 | 43582 | 44044 | BV106_RS22635 | hypothetical protein |
| 17.698 | AATTATTCATTTGAACACTA | NZ_BATR01000118.1 | 2976 | 2996 | 1 | 17 | 1 | 3013 | 3469 | BV106_RS21455 | HNH endonuclease |
| 17.6756 | ATGTGTTCAAATGAACTGTA | NZ_BATR01000104.1 | 796 | 816 | 1 | 147 | -1 | 0 | 649 | BV106_RS18300 | hypothetical protein |
| 17.6756 | ATGTGTTCAAATGAACTGTA | NZ_BATR01000104.1 | 796 | 816 | 1 | 10 | 1 | 826 | 1900 | BV106_RS18305 | radical SAM protein |
| 17.6193 | TAGTGTTCAAATGAATAATT | NZ_BATR01000118.1 | 2976 | 2996 | -1 | 17 | 1 | 3013 | 3469 | BV106_RS21455 | HNH endonuclease |
| 17.587 | TACAGTTCATTTGAACACAT | NZ_BATR01000104.1 | 796 | 816 | -1 | 147 | -1 | 0 | 649 | BV106_RS18300 | hypothetical protein |
| 17.587 | TACAGTTCATTTGAACACAT | NZ_BATR01000104.1 | 796 | 816 | -1 | 10 | 1 | 826 | 1900 | BV106_RS18305 | radical SAM protein |
| 15.6537 | ACCTGTTCGTTCAAACAAAA | NZ_BATR01000168.1 | 392 | 412 | -1 | 106 | -1 | 0 | 286 | BV106_RS30390 | hypothetical protein |
| 15.5081 | GAGTGTCCTTTTGAACACTG | NZ_BATR01000046.1 | 3419 | 3439 | -1 | 28 | 1 | 3467 | 4058 | BV106_RS06945 | hypothetical protein |
| 15.4983 | TTTTGTTTGAACGAACAGGT | NZ_BATR01000168.1 | 392 | 412 | 1 | 106 | -1 | 0 | 286 | BV106_RS30390 | hypothetical protein |
| 15.4426 | ATGTGTTCGCCAGAACAGGG | NZ_BATR01000021.1 | 29116 | 29136 | -1 | 49 | 1 | 29185 | 31362 | BV106_RS03920 | recombinase RecQ |
| 15.4034 | CCCTGTTCTGGCGAACACAT | NZ_BATR01000021.1 | 29116 | 29136 | 1 | 49 | 1 | 29185 | 31362 | BV106_RS03920 | recombinase RecQ |
| 15.3875 | AACTGTTCACGGAAACATTG | NZ_BATR01000047.1 | 14251 | 14271 | -1 | 1338 | -1 | 11740 | 12913 | BV106_RS07140 | hypothetical protein |
| 15.2682 | CAATGTTTCCGTGAACAGTT | NZ_BATR01000047.1 | 14251 | 14271 | 1 | 1338 | -1 | 11740 | 12913 | BV106_RS07140 | hypothetical protein |
| 15.1352 | TGCTGTTCAATAGAACTGTG | NZ_BATR01000171.1 | 45722 | 45742 | -1 | 22 | -1 | 44749 | 45700 | BV106_RS30925 | hypothetical protein |
| 15.07 | CACAGTTCTATTGAACAGCA | NZ_BATR01000171.1 | 45722 | 45742 | 1 | 22 | -1 | 44749 | 45700 | BV106_RS30925 | hypothetical protein |
| 14.9603 | TGATGTTCATTTGAATACTG | NZ_BATR01000081.1 | 97131 | 97151 | 1 | 459 | -1 | 95703 | 96672 | BV106_RS12405 | hypothetical protein |
| 14.9603 | TGATGTTCATTTGAATACTG | NZ_BATR01000081.1 | 97131 | 97151 | 1 | 36 | 1 | 97187 | 97865 | BV106_RS12410 | hypothetical protein |
| 14.89 | CAGTATTCAAATGAACATCA | NZ_BATR01000081.1 | 97131 | 97151 | -1 | 459 | -1 | 95703 | 96672 | BV106_RS12405 | hypothetical protein |
| 14.89 | CAGTATTCAAATGAACATCA | NZ_BATR01000081.1 | 97131 | 97151 | -1 | 36 | 1 | 97187 | 97865 | BV106_RS12410 | hypothetical protein |
| 14.7878 | CAGTGCACATTTGAACATCA | NZ_BATR01000149.1 | 12992 | 13012 | 1 | 57 | -1 | 12701 | 12935 | BV106_RS26735 | hypothetical protein |
| 14.6972 | TGATGTTCAAATGTGCACTG | NZ_BATR01000149.1 | 12992 | 13012 | -1 | 57 | -1 | 12701 | 12935 | BV106_RS26735 | hypothetical protein |
| 14.6528 | TGGTGTTTATTTGACCACCT | NZ_BATR01000036.1 | 17201 | 17221 | 1 | 168 | -1 | 11492 | 17033 | BV106_RS05915 | excinuclease ABC subunit A |
| 14.5039 | TCGTGTTCATGAGTACATTG | NZ_BATR01000164.1 | 27158 | 27178 | -1 | 474 | 1 | 27652 | 28414 | BV106_RS29285 | hypothetical protein |
| 14.5007 | CAGTGTTCAAAAGGACACTC | NZ_BATR01000046.1 | 3419 | 3439 | 1 | 28 | 1 | 3467 | 4058 | BV106_RS06945 | hypothetical protein |
| 14.465 | CAATGTACTCATGAACACGA | NZ_BATR01000164.1 | 27158 | 27178 | 1 | 474 | 1 | 27652 | 28414 | BV106_RS29285 | hypothetical protein |
| 14.4326 | AGGTGGTCAAATAAACACCA | NZ_BATR01000036.1 | 17201 | 17221 | -1 | 168 | -1 | 11492 | 17033 | BV106_RS05915 | excinuclease ABC subunit A |
| 13.8308 | TGGTGTTCAAATGAACTTGT | NZ_BATR01000003.1 | 1910 | 1930 | -1 | 324 | -1 | 337 | 1586 | BV106_RS00575 | putative DNA modification/repair radical SAM protein |
| 13.8088 | ACAAGTTCATTTGAACACCA | NZ_BATR01000003.1 | 1910 | 1930 | 1 | 324 | -1 | 337 | 1586 | BV106_RS00575 | putative DNA modification/repair radical SAM protein |
| 13.6858 | CACTGTTCGATTGTGCAGTC | NZ_BATR01000061.1 | 34520 | 34540 | 1 | 13 | -1 | 33898 | 34507 | BV106_RS09795 | repressor LexA |
| 13.6858 | CACTGTTCGATTGTGCAGTC | NZ_BATR01000061.1 | 34520 | 34540 | 1 | 69 | 1 | 34609 | 35404 | BV106_RS09800 | hypothetical protein |
| 13.6544 | GACTGCACAATCGAACAGTG | NZ_BATR01000061.1 | 34520 | 34540 | -1 | 13 | -1 | 33898 | 34507 | BV106_RS09795 | repressor LexA |
| 13.6544 | GACTGCACAATCGAACAGTG | NZ_BATR01000061.1 | 34520 | 34540 | -1 | 69 | 1 | 34609 | 35404 | BV106_RS09800 | hypothetical protein |
| 13.5712 | ACGTGTTCAATTGTACTCTT | NZ_BATR01000010.1 | 54866 | 54886 | 1 | 127 | -1 | 54334 | 54739 | BV106_RS01865 | hypothetical protein |
| 13.5454 | AAGAGTACAATTGAACACGT | NZ_BATR01000010.1 | 54866 | 54886 | -1 | 127 | -1 | 54334 | 54739 | BV106_RS01865 | hypothetical protein |
| 13.2608 | AACTGTCTTTTTGAACATTA | NZ_BATR01000031.1 | 64984 | 65004 | 1 | 146 | -1 | 64340 | 64838 | BV106_RS05225 | hypothetical protein |
| 13.2608 | AACTGTCTTTTTGAACATTA | NZ_BATR01000031.1 | 64984 | 65004 | 1 | 83 | 1 | 65087 | 67835 | BV106_RS05230 | hypothetical protein |
| 13.1577 | TTGTGTTCAATGAAAAACTC | NZ_BATR01000019.1 | 12795 | 12815 | 1 | 66 | -1 | 12408 | 12729 | BV106_RS03400 | thiol reductase thioredoxin |
| 13.0077 | ATTTGTTCGATTGAGCGGTA | NZ_BATR01000104.1 | 30466 | 30486 | -1 | 625 | -1 | 29202 | 29841 | BV106_RS18395 | hypothetical protein |
