# Supplementary material for: The Verrucomicrobia LexA-Binding Motif: Insights into the Evolutionary Dynamics of the SOS Response
Source: Front Mol Biosci. 2016 Jul 20;3:33. doi: 10.3389/fmolb.2016.00033 (PMC4951493; doi:10.3389/fmolb.2016.00033)
Supplement: Supplementary file 5 [file DataSheet1.DOCX]

Supplementary Material

The Verrucomicrobia LexA-binding Motif: Insights into the Evolutionary Dynamics of the SOS Response

Ivan Erill^1^, Susana Campoy^2^, Sefa Kılıç^1^ and Jordi Barbé^2*^

*** Correspondence:** Jordi Barbé, jordi.barbe@uab.cat

Supplementary material 5 – List S1 – FASTA sequences for promoters of detected *lexA* orthologs. Predicted Verrucomicrobia LexA-binding sites are highlighted in yellow (primary) and green (secondary). Predicted translation start sites are shown in red and underlined. Complete sequence information is available on each FASTA header.

>646713453 transcriptional repressor, LexA family [Coraliomargarita akajimensis DSM 45221 chromosome: NC_014008] (+)strand

AGTAGTTCCCAGCAAGCTAGATCCGCCCTTGACACTGTTCAGTTGTTCACCTAAACAAGTGAACACTATGCGAGACCTG

>641693724 SOS-response transcriptional repressor, LexA [Opitutus terrae PB90-1: NC_010571] (-)strand

CGGCGGAATCGTCAGCCGGAGCTTGCCAAGCGGGCGGCCCGGCTGTTCGCTTGAACATGTGTTCAAGAGAACACATCCGCCATGCTCACCGAA

>642329995 SOS-response transcriptional repressor, LexA [Verrucomicrobium spinosum DSM 4136, unfinished sequence: NZ_ABIZ01000001] (-)strand

CCGGCTCCAAGCAGAAAACACGAATGGCCAAAAAACTCTTGACTGCACAACCGAACAGTGTTCACATCTAACCATGCTTACGGAG

>2519011209 SOS-response transcriptional repressor, LexA [Rubritalea marina DSM 17716 : F454DRAFT_scaffold00008.8] (+)strand

CCTTTAGAATTTAATGTTGCATCATCATGATGATAAACTACTATGTTCTTAAGAACAGTGTTCAAATTATGAGTGATGCATTAACAAAGCGCCAACAAGAG

>2585418854 SOS-response transcriptional repressor, LexA [Rubritalea squalenifaciens DSM 18772 : EJ93DRAFT_scaffold00003.3] (+)strand

AAGCTGAAATAATCCTTGCCTGAATGGGGATATCAAGGCATTGTGTTCTTAAGAACAGTGTTCAATTAGGTCATGGCAAAAGGA

>2524330809 SOS-response transcriptional repressor, LexA [Verrucomicrobia bacterium SCGC AAA027-I19 : D412DRAFT_2518285663.28] (-)strand

ATTTACTTATTTTTTTCTTGTCAGACTGCGTATTATTTTGTTTTGTTCTGTCGAACACTGTTCATCAGATGAGCTCAAAC

>2582639043 SOS-response transcriptional repressor, LexA [Composite genome from Lake Mendota Epilimnion pan-assembly MEint.metabat.12173 : ME12173DRAFT_MEint_metabat_12173_383000059.100] (+)strand

ATTTACTTATTTTTTTCTTGTCAGACTGCGTATTATTTTGTTTTGTTCTGTCGAACACTGTTCATCAGATGAGCTCAAAC

>2582643281 SOS-response transcriptional repressor, LexA [Composite genome from Lake Mendota Epilimnion pan-assembly MEint.metabat.12612 : ME12612DRAFT_MEint_metabat_12612_36000558.11] (+)strand

CGCTGAAAAAGTTTCTTTACTCCGGTGCGCACATCGGTGAATTTGTTCTCAAGAACACTGTTCAACGAACCATGGCACACAAC

>2595246978 repressor LexA [Composite genome from Trout Bog Hypolimnion pan-assembly TBhypo.metabat.2519.v2 : TH02519DRAFT_TH02519_TBL_comb47_HYPODRAFT_10003679.37] (-)strand

ATTTTGAGTTGTGAGTTTAGTCTTGTGCCTAGGCAATAATTAGTGTTCAAAAGAACACTGTTCATATGAGTAAGCAA

>2595259627 repressor LexA [Composite genome from Trout Bog Epilimnion pan-assembly TBepi.metabat.1800.v2 : TE01800DRAFT_TE01800_TBL_comb48_EPIDRAFT_1000765.31] (+)strand

ATTTTGAGTTGTGAGTTTAGTCTTGTGCCTAGGCAATAATTAGTGTTCAAAAGAACACTGTTCATATGAGTAAGCAA

>2612357500 repressor LexA [Verrucomicrobium sp. BvORR106 : Ga0056857_1061] (-)strand

CCGGCTCCAAGTGGAAAACACAAATGGCCAAAAAACTCTTGACTGCACAATCGAACAGTGTTCACATCTAACCATGCTTACGGAG

>2612380763 repressor LexA [Verrucomicrobium sp. BvORR034 : Ga0056855_1024] (-)strand

CCGGCTCCAAGCGGAAAACACGAATGACCAAAAAACTCTTGACTGCACAATCGAACAGTGTTCACATCTAACCATGCTTACAGAG

>2612405553 repressor LexA [Haloferula sp. BvORR071 : Ga0056856_103] (+)strand

AAATCCACGAATTTTCGCTTGCCGTAATTTCACGACCATGGTTTGTTCCTAAGAACACTGTTCACTAAGCCATGCACGAAGGG

>2619624192 repressor LexA [Verrucomicrobia bacterium SCGC AAA027-I19 (contamination screened) : Ga0073400_111] (-)strand

ATTTACTTATTTTTTTCTTGTCAGACTGCGTATTATTTTGTTTTGTTCTGTCGAACACTGTTCATCAGATGAGCTCAAAC

>2620029940 repressor LexA [Verrucomicrobiaceae bacterium EBPR_Bin_208 : Ga0073653_1171] (+)strand

ATAAAATTCGGATTTCCCCTTGCGCACGATGAAAATCGGAGAATGTTCTCTCAAACAGTGTTCAGAATGAAGTTAACCTCACGCCAACAAGAA

>2620032610 repressor LexA [Verrucomicrobiaceae bacterium EBPR_Bin_287 : Ga0073654_1048] (-)strand

CTAAAAGGCCGATTTTTGCTTGCGCACGGTGAAAATTATCTGATGTTCTGTGGAACAGTGTTCATAATGAAATTAACC

>2632217020 repressor LexA [Verrucomicrobia bacterium IMCC26134 : Ga0077870_11] (-)strand

ACTGCGGGGCAAAATAAGAAAGCTGGAACCGCTGCCGGTCATGTGTTCACTTGAACACGGTTCACAAGAACACACCGCCATGCAACTCACC

>2634851374 repressor LexA [Opitutae-129 (UID2982) : Ga0081615_1035] (-)strand

AGAAATTGCAGCTATAGACGACACTTGATTTTTCTAAATAAGGTGTTCATTTGAACCGTGTTCAATTGAACCCTATGTTAACCGAA

>2582752176 SOS-response transcriptional repressor, LexA [Composite genome from Lake Mendota Epilimnion pan-assembly MEint.metabat.3880 : ME3880DRAFT_MEint_metabat_3880_82002817.45] (-)strand

TTGATCCGAAATTTCCTCTTGATCGGGTCCGCATCGCATGCTTTGTTCCCTCGAACACTGTTCAAAATCATGCACACTAAC

>2617266857 repressor LexA [Dpulchra_bleached_metagenome_bin377 Ga0073125 : Ga0073125_1201] (+)strand

GCTGGTCGAATTCGACTTGCGCAAAAGCTTAAAATAGATACTATGTTCTATAGAACACTACTCTTATGCATAGTATT

>2236430230 repressor LexA [Verrucomicrobia SCGC AAA164-E04 : A164E4DRAFT_NODE-unique_4_len_173494.4] (+)strand

TCCTTTTTATTTCTGGGATTGACACGATCAGGTGTCAATATACTGTTCAAATGAACAGTATGAGTGAAGGTTTAACAAA

>642912055 SOS-response transcriptional repressor, LexA [Chthoniobacter flavus Ellin428, unfinished sequence: NZ_ABVL01000017] (-)strand

TCCATGCAAAATGCTGCTTGCGTTGACTCTTCGAAAGACGCAGTGTTCTGGCGAACATGATTCTTACGCAGCGCCAACAAG

>2236434052 SOS regulatory protein LexA [Verrucomicrobia SCGC AAA168-E21 : A168E21DRAFT_NODE-unique_18_len_31805.18] (+)strand

TTAGATGAGGGGGGTAAGCTGATATTTCCGACTGGACAAATAGTGTTCATTGAAACAATAGTCTTACATGGCTCACAAA

>2236454024 SOS regulatory protein LexA [Verrucomicrobia SCGC AAA168-F10 : A168F10DRAFT_NODE-unique_23_len_53784.23] (-)strand

TTAGATGAGGGGGGTAAGCTGATATTTCCGACTGGACAAATAGTGTTCATTGAAACAATAGTCTTACATGGCTCACAAA

>2609753506 repressor LexA [Chthoniobacterales bacterium JGI 000193CP-H04 : Ga0069920_122] (+)strand

CCGAGGCGGCGGTGAGGGCCATGAAGGGGAAACGCAACGTGTGTGTTCATTTGAACTTGCGCCACCGTTATTTTTCCGCATGATT

>2510268627 repressor LexA [Opitutaceae sp. TAV5 : Opit5_Contig145.1] (+)strand

ATTGAATAAGCACATTTTGAGCATTGGAAAATATTCAGATATGTGTTCATTCGAACACTTATTGATAGTTACGCA

>2510269383 repressor LexA [Opitutaceae sp. TAV5 : Opit5_Contig145.1] (+)strand

TCTAAAATATTGCATTTCGGGTGTTGGAATGTCTGGCTATATGTGTTCACTTGAACACTAGGAGTATCCAGCCCTATCGAACAATCCAACGCCATGCTCACCGAA

>2517879136 repressor LexA [Verrucomicrobia bacterium SCGC AAA164-O14 (genbank_version) : VerrucomO14_gi399212144.708] (-)strand

TTAGATGAGGGGGGTAAGCTGATATTTCCGACTGGACAAATAGTGTTCATTGAAACAATAGTCTTACATGGCTCACAAA

>2517882839 repressor LexA [Verrucomicrobia bacterium SCGC AAA168-E21 (genbank_version) : VerrucomE21_gi399210541.702] (+)strand

TTAGATGAGGGGGGTAAGCTGATATTTCCGACTGGACAAATAGTGTTCATTGAAACAATAGTCTTACATGGCTCACAAA

>2517885782 repressor LexA [Verrucomicrobia bacterium SCGC AAA168-F10 (genbank_version) : VerrucomF10_gi399210541.702] (+)strand

TTAGATGAGGGGGGTAAGCTGATATTTCCGACTGGACAAATAGTGTTCATTGAAACAATAGTCTTACATGGCTCACAAA

>2582880774 SOS-response transcriptional repressor, LexA [Composite genome from Trout Bog Epilimnion pan-assembly TBepi.metabat.4605 : TE4605DRAFT_TBepi_metabat_4605_1003476.185] (-)strand

ACCGGCTCAGGAAAACATCTTGCGTAAAGGGCATGAGAGGGAATGTTCGCCCGAACAGCCCTTATTCCTCATGCTCACCGAC

>2583004831 repressor LexA [Composite genome from Trout Bog Hypolimnion pan-assembly TBhypo.metabat.4590 : TH4590DRAFT_TBhypo_metabat_4590_10019306.101] (-)strand

TACGCAGCTGGCCGCAATTGCGGCTTGCGGCGGCGGGATCGCGTGTTCAATTGAACACATCGTGGCCGAAGCT

>2518030603 repressor LexA [Verrucomicrobium sp. 3C : A37ADRAFT_scaffold1.1] (-)strand

CATGCCGATTTTGGCAGGCGAGGCGGCTGTCCACTCGGGAAAACATTTGACAAGCTTTCTTTACGCGATCACTGATTCGTTATGCTCGTGGAT

>2582646426 repressor LexA [Composite genome from Lake Mendota Epilimnion pan-assembly MEint.metabat.12657 : ME12657DRAFT_MEint_metabat_12657_66000142.74] (+)strand

CCGCACCTGAGGCCCTAGTCTTGACAGTGAACAAACATTCATAGAACATCCGTTCACCATGGAAGCACTC

>2582697965 repressor LexA [Composite genome from Lake Mendota Epilimnion pan-assembly MEint.metabat.2014 : ME2014DRAFT_MEint_metabat_2014_464002082.160] (-)strand

TGGCCTTTTGGCCTGTCGGCTTGACAGTGAACAAGTTTTCATCGAACATGCGTTCACTATGGAAGAACTG

>2582726124 repressor LexA [Composite genome from Lake Mendota Epilimnion pan-assembly MEint.metabat.30509 : ME30509DRAFT_MEint_metabat_30509_2300002348.59] (+)strand

CGCAAAGGATAGCCGATTTTTGTTGCGAAGCCTAGAACAAACGTTCTATACTGCCCGCATGTCACCTCGA

>2582844276 repressor LexA [Composite genome from Trout Bog Epilimnion pan-assembly TBepi.metabat.1301 : TE1301DRAFT_TBepi_metabat_1301_1000095.97] (-)strand

CGGACTAAAACAGCCGAGGCTTGACAGTGAGCAAAATTTCATTGAACATCCGTTCACTATGCAAGGTCTC

>2541015653 repressor LexA [Verrucomicrobium sp. LP2A : G346DRAFT_scf7180000000012_quiver.2] (+)strand

GCTCCGTCGCCCCGGGAAAACGTTTGACAAGTCTTTTCCGGCCTTCCACTGATGTCTTATGCTCGTGGAT

>2582948466 SOS-response transcriptional repressor, LexA [Composite genome from Trout Bog Hypolimnion pan-assembly TBhypo.metabat.2747 : TH2747DRAFT_TBhypo_metabat_2747_10002879.165] (-)strand

TTGTGAACAATTCCGGGTTGACAAGGGGACGTGGTGAAATAGTGTCTAAACAGACACTATGAAAGCATTG

>2582960083 repressor LexA [Composite genome from Trout Bog Hypolimnion pan-assembly TBhypo.metabat.3004 : TH3004DRAFT_TBhypo_metabat_3004_10005036.223] (+)strand

CAAGCACAAACCGCGCCTTGACAGGCCAGCCCCCTAATAGTATCCATGTGGATACTATATGACCGGATTG

>2582988720 SOS-response transcriptional repressor, LexA [Composite genome from Trout Bog Hypolimnion pan-assembly TBhypo.metabat.4093 : TH4093DRAFT_TBhypo_metabat_4093_10001519.100] (+)strand

CGTTAATATTCGAAAAAACTATGTTTGCCGGAATCTCTGCCAATAAAGGAGACGACTAATGAAAGCTCTT

>641691955 SOS-response transcriptional repressor, LexA [Opitutus terrae PB90-1: NC_010571] (-)strand

ACAACATGGCACATGAGTGTTCATCGATTTACCTTGCAAACAACACGATCCTGCACAAATGTGCAGCATG
