# Supplementary figures and images for: The Verrucomicrobia LexA-Binding Motif: Insights into the Evolutionary Dynamics of the SOS Response
Source: Front Mol Biosci. 2016 Jul 20;3:33. doi: 10.3389/fmolb.2016.00033 (PMC4951493; doi:10.3389/fmolb.2016.00033)

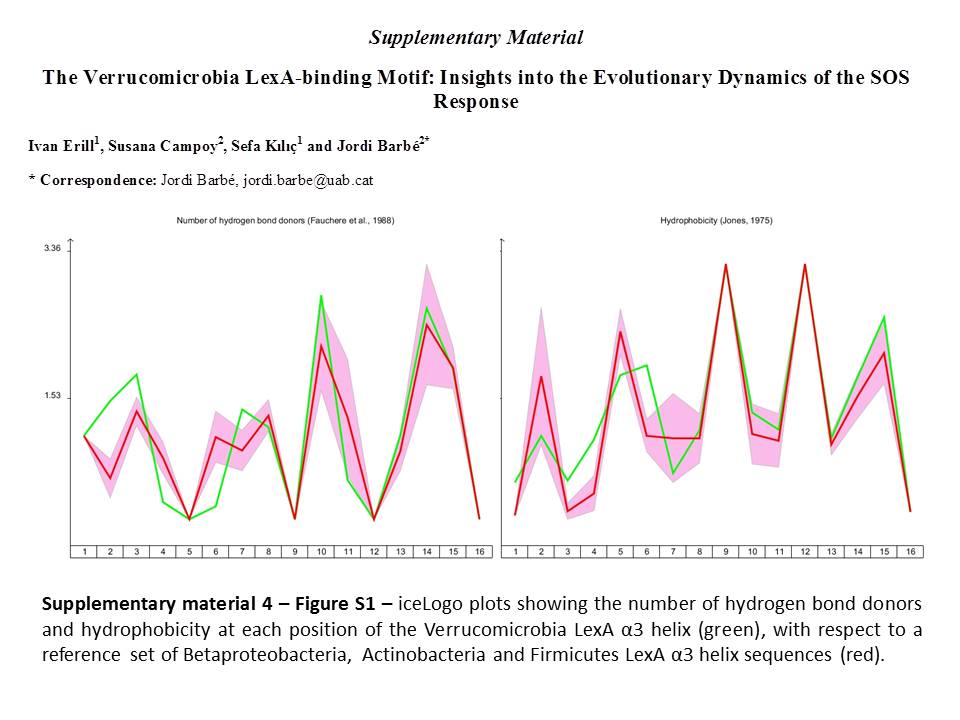

Supplement: Supplementary file 6 [file Image1.JPEG]

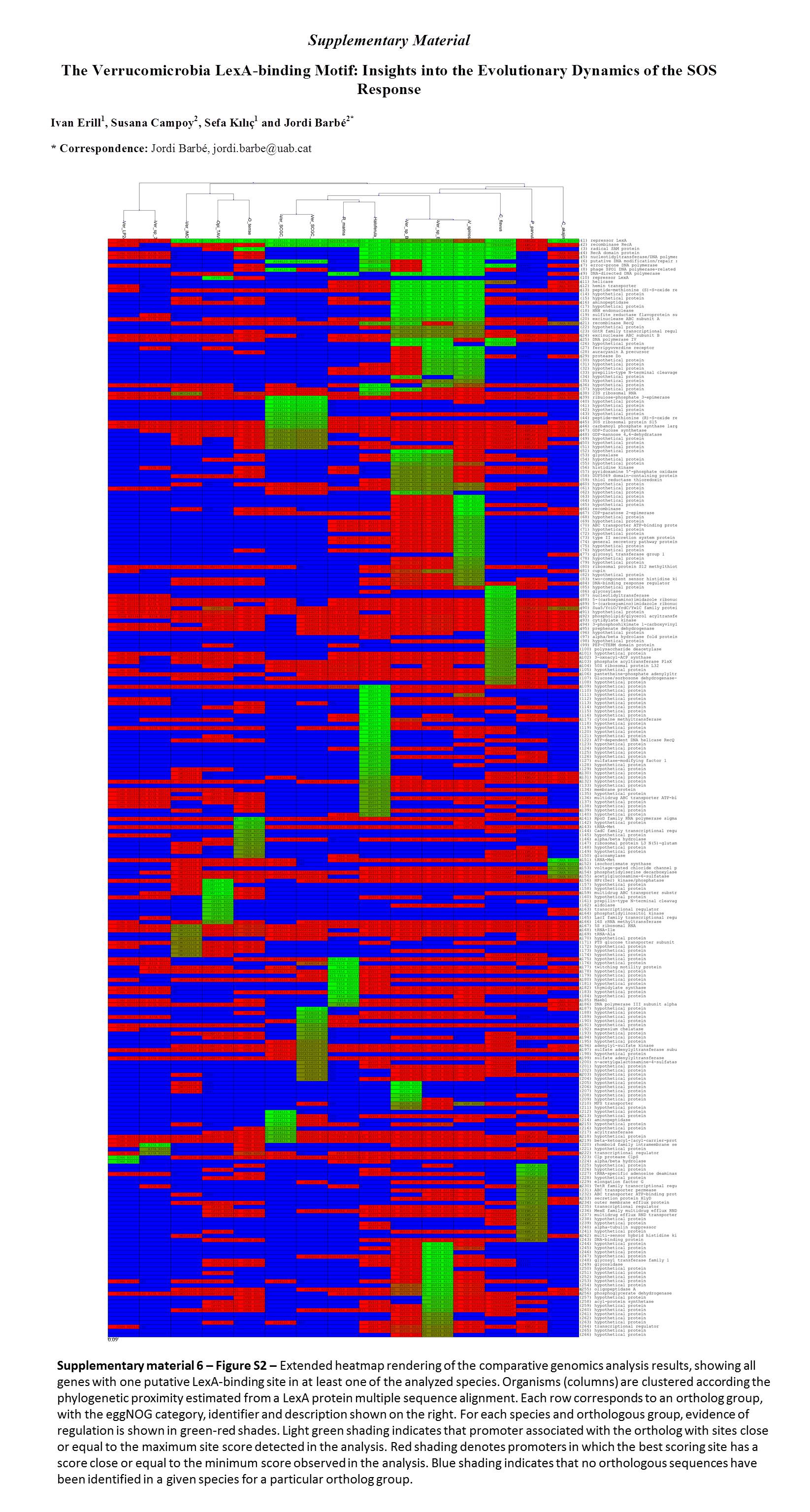

Supplement: Supplementary file 7 [file Image2.JPEG]
